# High Diversity in Cretaceous Ichthyosaurs from Europe Prior to Their Extinction

**DOI:** 10.1371/journal.pone.0084709

**Published:** 2014-01-21

**Authors:** Valentin Fischer, Nathalie Bardet, Myette Guiomar, Pascal Godefroit

**Affiliations:** 1 Department of Geology, University of Liège, Liège, Belgium; 2 Operational Directory ‘Earth and History of Life’, Royal Belgian Institute of Natural Sciences, Brussels, Belgium; 3 CNRS UMR 7207, Département Histoire de la Terre, Muséum National d'Histoire Naturelle, Paris, France; 4 Réserve naturelle géologique de Haute Provence, Digne-les-bains, France; Raymond M. Alf Museum of Paleontology, United States of America

## Abstract

**Background:**

Ichthyosaurs are reptiles that inhabited the marine realm during most of the Mesozoic. Their Cretaceous representatives have traditionally been considered as the last survivors of a group declining since the Jurassic. Recently, however, an unexpected diversity has been described in Upper Jurassic–Lower Cretaceous deposits, but is widely spread across time and space, giving small clues on the adaptive potential and ecosystem control of the last ichthyosaurs. The famous but little studied English Gault Formation and ‘greensands’ deposits (the Upper Greensand Formation and the Cambridge Greensand Member of the Lower Chalk Formation) offer an unprecedented opportunity to investigate this topic, containing thousands of ichthyosaur remains spanning the Early–Late Cretaceous boundary.

**Methodology/Principal Findings:**

To assess the diversity of the ichthyosaur assemblage from these sedimentary bodies, we recognized morphotypes within each type of bones. We grouped these morphotypes together, when possible, by using articulated specimens from the same formations and from new localities in the Vocontian Basin (France); a revised taxonomic scheme is proposed. We recognize the following taxa in the ‘greensands’: the platypterygiines ‘*Platypterygius*’ sp. and *Sisteronia seeleyi* gen. et sp. nov., indeterminate ophthalmosaurines and the rare incertae sedis *Cetarthrosaurus walkeri*. The taxonomic diversity of late Albian ichthyosaurs now matches that of older, well-known intervals such as the Toarcian or the Tithonian. Contrasting tooth shapes and wear patterns suggest that these ichthyosaurs colonized three distinct feeding guilds, despite the presence of numerous plesiosaur taxa.

**Conclusion/Significance:**

Western Europe was a diversity hot-spot for ichthyosaurs a few million years prior to their final extinction. By contrast, the low diversity in Australia and U.S.A. suggests strong geographical disparities in the diversity pattern of Albian–early Cenomanian ichthyosaurs. This provides a whole new context to investigate the extinction of these successful marine reptiles, at the end of the Cenomanian.

## Introduction

Ichthyosauria was a successful clade of marine sauropsids that spanned most of the Mesozoic, from the Olenekian (Early Triassic) to the end of the Cenomanian (Late Cretaceous). When compared to the Triassic and the Jurassic, the Cretaceous record of ichthyosaurs is generally poor [1]. As a result, only minimal attention has been drawn to the Cretaceous representatives of Ichthyosauria in the past. The last in-depth taxonomic reviews of Cretaceous ichthyosaurs are those of McGowan [2], focusing on North American material, and Bardet [3], mainly reviewing Late Cretaceous ichthyosaur occurrences. McGowan [2] merged all valid species within a single genus, *Platypterygius*. Cretaceous ichthyosaurs were then considered as undiversified, despite their worldwide distribution (e.g. [4]). Their extinction, at the Cenomanian–Turonian boundary [3], was therefore considered as inconsequential because the group was already on the decline since the Jurassic [5]. This vision of ichthyosaur evolution has been substantiated by recent reassessments of the abundant Australian and American material, which regarded both these assemblages as monospecific: ‘*Platypterygius*’ *australis* in Australia [6]–[13] and ‘*Platypterygius*’ *americanus* in U.S.A. [14]. Yet, numerous new forms have recently been described in Canada and western Eurasia, profoundly modifying the traditional view of ichthyosaur's protracted decline in the Cretaceous [1], [15]–[23].

However, these recent findings are widely spread across time (Berriasian–Albian, around 46 Myr) and space (Canada, Argentina, England, Germany, and Russia), and evidence of co-occurring taxa is extremely scarce. Indeed, only three Cretaceous formations have yielded more than one ichthyosaur taxon: the Wabiskaw Member of the Clearwater Formation (early Albian of Canada; two taxa [22], [23]), the Loon River Formation (middle Albian of Canada; two taxa [15], [16]), and an unnamed formation from the Barremian of Russia (likely two taxa [21]). Therefore, although recent data indicates ichthyosaurs were not a ‘dying group’ as previously supposed, this new data gives little clues on the ecological diversity and ecosystem control of the Cretaceous ichthyosaurs: were Cretaceous ichthyosaurs a frequent but minor component of marine trophic webs or did they occupy several ecological niches within marine ecosystems as they did in the past (e.g. Early Jurassic Europe [24], [25])? Answering this question requires geological formations containing numerous marine tetrapods – a rare resource in the Early Cretaceous strata – but does not necessarily require articulated specimens.

Here, we analyze the diversity of Albian–basal Cenomanian ichthyosaur assemblages of western Europe, by focusing on the Albian Gault Formation (UK), the Albian–Cenomanian Upper Greensand Formation (UK), the basal Cenomanian Cambridge Greensand Member (base of the Lower Chalk Formation, UK), and the Albian part of the Marnes Bleues Formation (France). The abundant material (several thousands specimens in total) from these localities provides precious data on the taxonomic and ecological diversity of some of the last representatives of Ichthyosauria. In order to evaluate this diversity, we (1) thoroughly reassess the taxonomy of the ichthyosaur assemblages from these formations and (2) evaluate the ecological diversity of these taxa by analyzing their tooth shape, tooth wear, and their relative abundances. Then, these western European assemblages are discussed within the worldwide context of ichthyosaur diversity during the Cretaceous by (3) plotting taxonomic richness curves and (4) evaluating geographical disparity of diversity, providing a background for future analyses of their final extinction.

## Materials and Methods

### Institutional abbreviations

CAMSM: Sedgwick Museum of Earth Sciences, Cambridge University, Cambridge, UK; CM: Carnegie Museum of Natural History, Pittsburg, PA, USA; IRSNB: Royal Belgian Institute of Natural Sciences, Brussels, Belgium; GLAHM: The Hunterian Museum, University of Glasgow, Glasgow, UK; LEICT: New Walk Museum & Art Gallery, Leicester, UK; MJML: Museum of Jurassic Marine Life, Wareham St Martin, UK; NHMUK: Natural History Museum, London, UK; RGHP: Réserve naturelle Géologique de Haute-Provence, Digne-les-bains, France; SSU: Saratov State University, Saratov, Saratov Oblast, Russia.

No permits were required for the described study, which complied with all relevant regulations.

### Nomenclatural acts

The electronic edition of this article conforms to the requirements of the amended International Code of Zoological Nomenclature, and hence the new names contained herein are available under that Code from the electronic edition of this article. This published work and the nomenclatural acts it contains have been registered in ZooBank, the online registration system for the ICZN. The ZooBank LSIDs (Life Science Identifiers) can be resolved and the associated information viewed through any standard web browser by appending the LSID to the prefix “http://zoobank.org/”. The LSID for this publication is: urn:lsid:zoobank.org:pub:C9E8AE62-3686-4483-8EEB-861B2DCB102C. The electronic edition of this work was published in a journal with an ISSN, and has been archived and is available from the following digital repositories: PubMed Central, LOCKSS, and ORBi.

### Assessment of the taxonomic diversity in bone-bed like deposits

#### Taxonomic diversity

Two bone-bed-like deposits have been investigated during this research: the Upper Greensand Formation and the Cambridge Greensand Member. Their faunal diversity must be cautiously assessed, because most of the material is disarticulated. In the sections below, we detail the methodology used to evaluate the taxonomic diversity of these remains and the relative abundances of each recognized taxon.

More than one thousand ichthyosaur specimens (without counting the isolated teeth) are held in the Cambridge Greensand Member collections of the CAMSM, IRSNB, GLAHM, LEICT, and NHMUK. Most of them are disarticulated and consist of isolated bones that were either purchased by or donated to these institutions. We accessed and analyzed all these collections; we used a simple, three-step process to assess the taxonomic diversity of these remains. First, we established morphotypes within each series of abundant and usually diagnostic bones (skull roof bones, teeth, humeri, and femora; see Table 1 for a list of the morphotype recognized and Text S5 for a determination key); however, all specimens and all kinds of fragments, including rostra, centra, ribs, gastralia, phalanges, etc. have been investigated. Then, we used articulated specimens from the upper (unreworked) part of the Cambridge Greensand Member and from coeval deposits of the Vocontian Basin (France) to group some of these morphotypes together. Finally, we compared these morphotypes or groups of morphotypes to known taxa in the literature in order to ‘translate’ these entities into taxa, when possible. However, we refrained from assessing the diversity at the specific level, especially because of the numerous problems related to the species currently referred to as ‘*Platypterygius*’ [18]. Moreover, the taxonomic value of the numerous small morphological variations observed in the sample is difficult to assess. Nevertheless, some bones, such as humeri and femora contain more distinct morphotypes than the number of taxa (genera) recognized, suggesting a higher diversity at a lower taxonomic level, probably reflecting the specific level. On the other hand, some of these morphotypes contain only a few specimens, so intraspecific variation should also be considered as a possible explanation for the high number of humeral and femoral morphotypes. Indeed, slight inter-adult and ontogenetic variability of humeral distal facets has been recognized in the platypterygiine ophthalmosaurid ‘*P.*’ *australis* [26], [27].

**Table 1:** Bone morphotypes recognized here and their assignation.

Bone	Morphotype	Assignation
Basioccipital	BM1	‘*Platypterygius*’ sp.
Basioccipital	BM2	*Sisteronia seeleyi*
Basioccipital	BM3	*Acamptonectes* sp.
Tooth	TM1	‘*Platypterygius*’ sp.
Tooth	TM2	*Sisteronia seeleyi*
Tooth	TM3	Ophthalmosaurinae indet.
Humerus	HM1	‘*Platypterygius*’ sp.
Humerus	HM2	*Sisteronia seeleyi*
Humerus	HM3	Ophthalmosaurinae indet.
Humerus	HM4	‘*Platypterygius*’ sp.
Femur	FM1	‘*Platypterygius*’ sp.
Femur	FM2	Ophthalmosauridae indet.
Femur	FM3	Ophthalmosauridae indet.
Femur	FM4	Ophthalmosauridae indet.
Femur	FM5	*Cetarthrosaurus walkeri*

The morphotype belong to *Cetarthrosaurus walkeri* is placed within the “Femur” category, as suggested by Seeley [106]. In the text, however, we opted for a more conservative position, considering this morphotype as a propodial, because of its unusual morphology.

All the specimens from these deposits cannot be determined, because isolated elements from the rostrum, mandible and axial skeleton are not diagnostic and because of the presence of small, probably juvenile specimens lacking distinguishing features, in addition to damaged specimens. In total, only 124 specimens of the Cambridge Greensand Member (without counting teeth and the three femur morphotypes belonging to Ophthalmosauridae indet. which are described in Text S6) have been assigned to one of the five infrafamilial taxa that we could recognize. Whatever these taxa might be, the Cambridge Greensand Member provides one of the largest samples of a Cretaceous ichthyosaur assemblage, worldwide.

#### Relative abundances

We counted all diagnosable isolated bones and articulated specimens to estimate the relative abundance of each taxon in the Cambridge Greensand Member. Articulated specimens were counted only once in the total count. Despite their diagnostic features, we did not consider teeth as reliable bones for abundance counts because reptiles shed their teeth; therefore, the relative abundance of tooth morphotypes partly reflects ethological habits and/or physiological features, polluting the signal.

#### Ecological diversity

We used absolute tooth size, tooth shape, and tooth wear qualitatively to assess the ecological diversity of the ichthyosaurs from the Cambridge Greensand Member and the Marnes Bleues Formation. Intrinsic properties of teeth (size, shape) give an idea of the optimal range of preys that could be processed (e.g. [25], [28]), whereas wear gives indications on the actual use of teeth by a single individual (e.g. [29], [30]). A more detailed and quantitative analysis, encompassing numerous craniodental features of Jurassic and Cretaceous taxa is currently in preparation and will be published elsewhere.

### Diversity curves

The temporal evolution of two variables is analysed here: the taxonomic diversity at the specific and the generic levels. Both are simple counts of the parvipelvian taxonomic richness for each time interval (the stage level), from the Hettangian (Early Jurassic) to the Turonian (Late Cretaceous). The dataset compiled is available in Text S7. Stages characterize periods of Earth's history with supposed rather constant climate, ocean dynamics, etc., but sometimes greatly differ in duration. Stage duration influences the number of specimens and thus the biodiversity. Rarefaction methods (e.g. [31]) cannot be employed here because numerous stages of Cretaceous record a very small number of specimens and should therefore be omitted from the analysis using this method. We divided the largest stages (Aptian and Albian) into their usual substages (lower and upper Aptian; lower, middle, and upper Albian), based on ammonite stratigraphy [32]–[38], rather than using temporal bins. The lower Aptian encompasses the ammonite zones from the *oglanlensis* Zone to the *furcata* Zone; the upper Aptian from *subdonosocostatum* Zone to the *Jacobi* Zone; the lower Albian from the *schrammeni*/*tardefurcata* Zone to the *mammlilatum*/*auritiformis* Zone; the middle Albian to the *dentatus* Zone to the *lautus* Zone; the upper Albian from the *cristatum* Zone to the *dispar*/*briacensis* Zone. Using numerical ages from Kuhnt & Moullade [39], Ogg et al. [40], Scott [35] and Gradstein et al. [41], time bins for the stages/substages from the Hettangian to the Turonian have a mean duration 5.06 My, but the standard deviation remains quite high (±2.25 My). At any rate, these durations should not be considered too strictly as the error margin for many stage boundaries can reach ±1 My, and the numerical age for the substages of the Aptian and Albian are extrapolations based on the calculations of sedimentations rates between dated horizons [35], [39]. Nevertheless, this permits to recover stage durations that are comparable. Moreover, this method of splitting the Aptian and the Albian is also useful for better understanding of the extinction of ichthyosaurs by providing a more precise evolution of ichthyosaur diversity near their extinction. But this approach does not mitigate other biases, such as collecting or environmental biases. Corrections exist for some of these factors [42]–[46] but this would move the results away from the ichthyosaur fossil record itself, an approach we are reluctant to undertake. This has the advantage of being intuitive and plotting ‘raw’ values, which are directly related to the fossil record itself and how we interpret it.

The specific and generic curves are simple counts of the taxa that we (or the scientific community) recognize as valid for each time bin and the stratigraphic range of each taxa is based on oldest and youngest unambiguous fossil evidences, thus regardless of any phylogenetic ghost lineages. Lazarus ranges are, however, taken into account: for example, if taxon A occurs during the early Hauterivian and the late Aptian, then we consider taxon A as a valid Barremian and early Aptian taxon as well. The problematic genus *Platypterygius* was considered as a single taxon in the generic curves, grouping all species currently referred to it. The generic and specific diversity curves for the Jurassic are added to provide a point of comparison.

### Geological setting

The specimens that we have examined are classified by country, and then by formation. Geographic, stratigraphic (encompassing bio- and lithostratigraphic data) and paleoecological data (focusing on the vertebrate content) are given for each formation, when available. These data were taken from the literature and from collaborative investigations and/or personal field observations.

#### Gault Formation, UK

**Figure 1:**
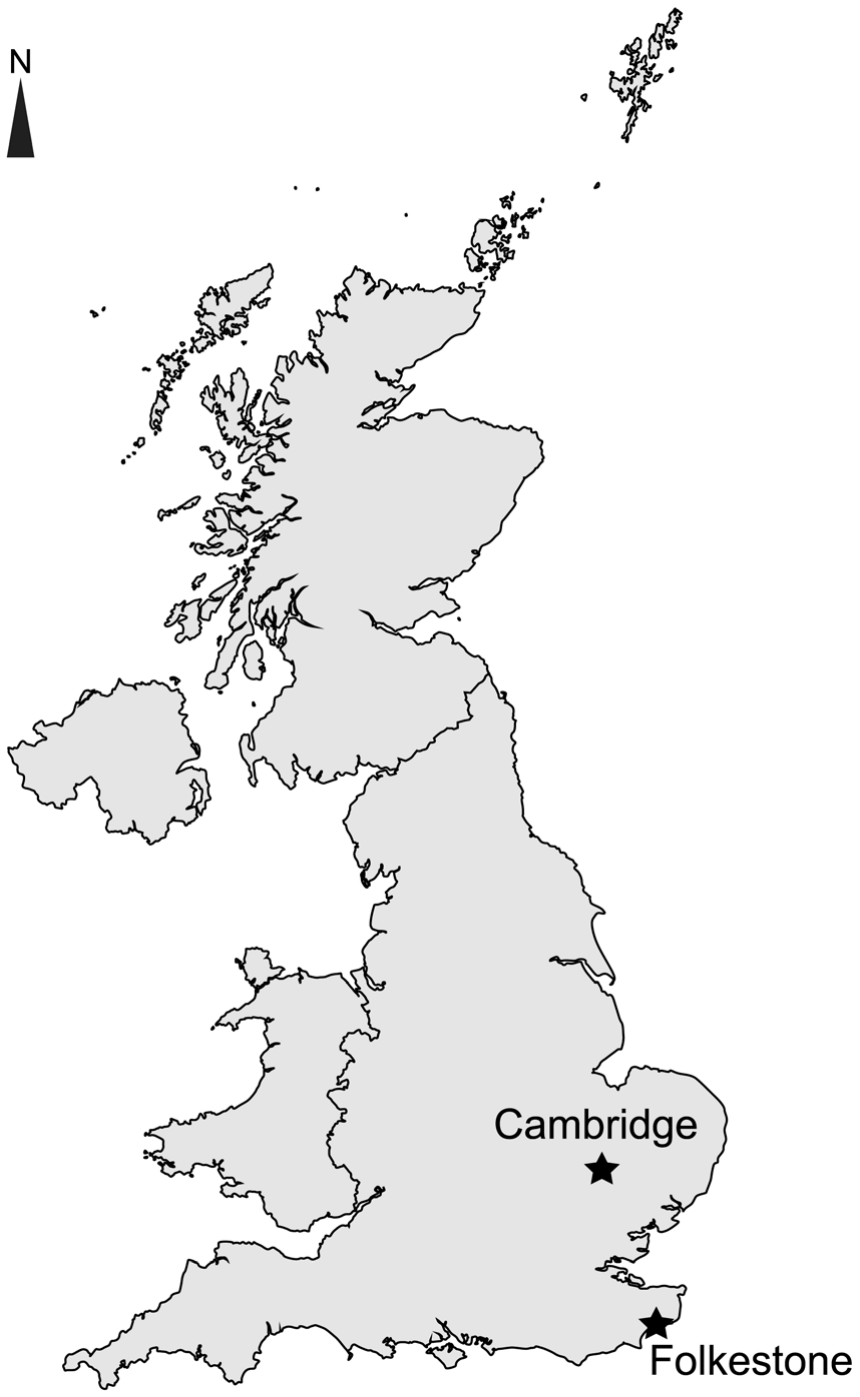
General location of the most important late Early Cretaceous ichthyosaur-bearing localities of England: Cambridge and Folkestone.

The Gault is a marl formation occurring in several basins of England, occurring in the East Midland Shelf, the Bedforshire ‘Straits’, the Wessex Basin, the Wealden Basin, the Vectian Basin [47]; i.e. the whole eastern, southeastern and southern margins of England. The ‘Gault’ is also recognized as a facies in adjacent basins; for instance, it possibly occurs in the French Paris Basin [33], [48], [49]. The data presented below is restricted to the Gault Formation, cropping out in the UK, notably in Folkestone (Figure 1).

The Gault Formation encompasses most of the Albian, and passes laterally to the Cambridge Greensand Member/Upper Greensand Formation towards the east [32], [47]. In the Cambridgeshire area, the Gault Formation is middle to late Albian in age, whereas its base extends up to the early Albian (*Tardefurcata* Zone) in the Wealden Basin [32], [47]. The fossil-rich locality of Folkestone lies within the Wealden Basin. Most of the Aptian–early Cenomanian English ichthyosaurs fossils studied here were collected during the 19^th^ century as ‘coprolites’ and subsequently acquired by museums [50]; accordingly, there is no precise stratigraphic data linked to these specimens.

The studied specimens from this formation are from the NHMUK collection (19 specimens; see Text S1). Note that the few Gault Formation ichthyosaurs held at CAMSM appear to be lost; we have been unable to locate them in Sedgwick Museum or in the ‘stores’ at Cambridge University.

#### Upper Greensand Formation, UK

The Upper Greensand Formation is a glauconitic sandstone reworked from the Gault Formation [47], [51]. The Upper Greensand Formation is distinct from the Cambridge Greensand Member. Both these deposits rework the Gault Formation, but they mostly occur in different basins (part of the Vectian and Wealden basins and part of the Bedforshire ‘Straits’ for the Upper Greensand Formation VS Southern and transitional Provinces for the Cambridge Greensand Member). When the two deposits co-exist (the Bedforshire ‘Straits’/Transitional Province, i.e. the Cambridgeshire area, Figure 1), they are separated by an unconformity with the time-gap of slightly variable duration (Hopson, pers. com. to V.F. June 2012). The onset of the Upper Greensand Formation appears diachronic; its total stratigraphic range is lower Albian to lower Cenomanian [47], whereas the Cambridge Greensand Member is strictly early Cenomanian in age [32].

Because both the Upper Greensand Formation and Cambridge Greensand Member can occur together and all specimens were collected without precise stratigraphic data, it is possible that some specimens were listed as belonging to the wrong ‘greensand’ deposit in the collection database. Text S2 lists all ichthyosaur specimens from the Upper Greensand Formation.

#### Cambridge Greensand member, UK

The Cambridge Greensand Member is a glauconitic and phosphatic sandstone forming the basal part of the Lower Chalk Formation in the Bedfordshire ‘Straits’ area/Transitional Zone (i.e. central England, East Anglia Massif) [47], [51], [52]. Hopson et al. [52] revised the stratigraphy of the English Upper Cretaceous. The ‘Lower Chalk’ of previous authors is called the Grey Chalk Subgroup, containing two formations in the central England zone: the West Melbury Marly Chalk Formation at the base, overlapped by the Zig Zag Chalk Formation. The Grey Chalk Group is strictly Cenomanian in age (*Mantelliceras mantelli* to *Calycoceras guerangeri* zones; [52]). The Cambridge Greensand Member constitutes the base of the West Melbury Marly Chalk Formation. Glauconitic chalk (the Glauconitic Chalk Member) lies over the Cambridge Greensand Member or the Upper Greensand Formation in some places [52]. Some important articulated specimens (e.g. CAMSM B58257_67, holotype of *Sisteronia seeleyi*) were deposited in this member, as testified by their mode of preservation.

The Cambridge Greensand Member was deposited during the early Cenomanian [53], but reworks the top of the Gault Formation [47], [51], [54]. The reworked fossils are phosphatized and late Albian in age ([51] and references therein). However, the uppermost part of this deposit contains unreworked, non-phosphatized early Cenomanian specimens embedded in a glauconitic chalk, possibly at the boundary or within the overlying Glauconitic Marl Member ([52], [55]; V.F. & N.B., pers. obs., contra Unwin [56]). This permits one to differentiate both assemblages, if needed. Martill & Unwin ([51] and references therein) indicated that the reworked specimen are not older than the *Calihoplites auritus* Subzone, and were therefore probably contemporaneous (i.e. ‘Vraconian’, see [35], [57]) with the large *Platypterygius hercynicus* of northwestern France (MHNH 2010.4; [18]). Microfossil evidence suggests that the time break between the reworked specimens from the Gault Formation and the ‘in-place’ early Cenomanian ones is probably small [53], although the base of this member is diachronous – as could be expected from such a transgressive/erosive deposit – becoming younger eastwards [58].

The Cambridge Greensand Member ichthyosaur material consists of several thousands specimens – mostly isolated teeth – and has never been reassessed thoroughly since Seeley's catalogue, published in 1869 [50]. Specimens are housed in the CAMSM, GLAHM, IRSNB, LEICT, and NHMUK collections; see Text S3.

#### The Marnes Bleues Formation, France

**Figure 2:**
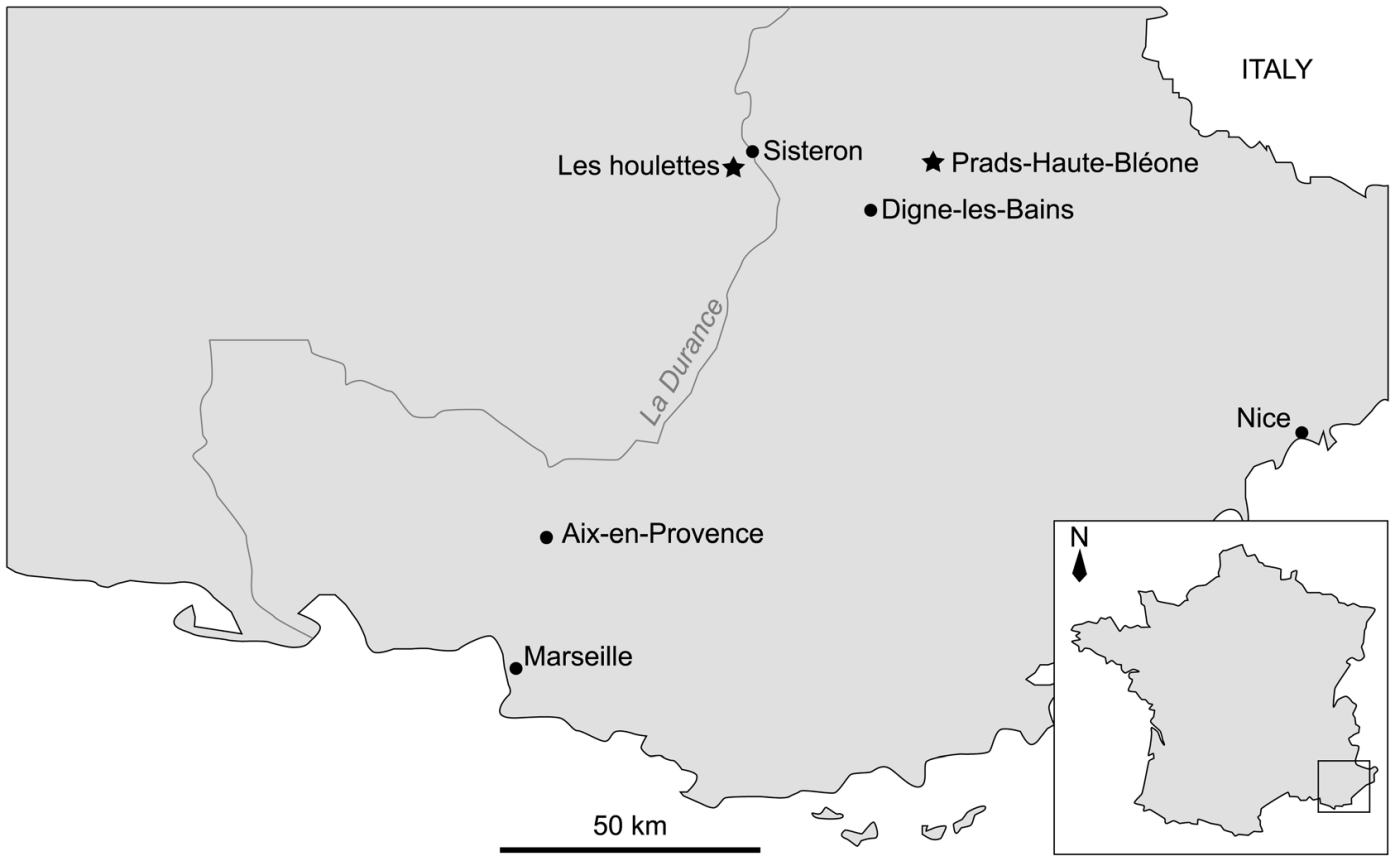
Location of the most important late Early Cretaceous ichthyosaur-bearing localities of the Vocontian Basin in Southeastern France. Stars indicate fossil-localities and plain circles indicate major cities.

The Marnes Bleues Formation was deposited during the Aptian and Albian in the Vocontian Basin [59]. The Vocontian Basin or Vocontian Trough was a deep, highly subsident Mesozoic basin located at the northwestern border of the Tethys, now southeastern France (Figure 2). It represents the deepest structural unit of the Dauphinois Basin, the Vercors carbonate platform representing its shallow part [60]. All southeastern France Albian ichthyosaur remains known so far were found in the Marnes Bleues Formation.

**Figure 3:**
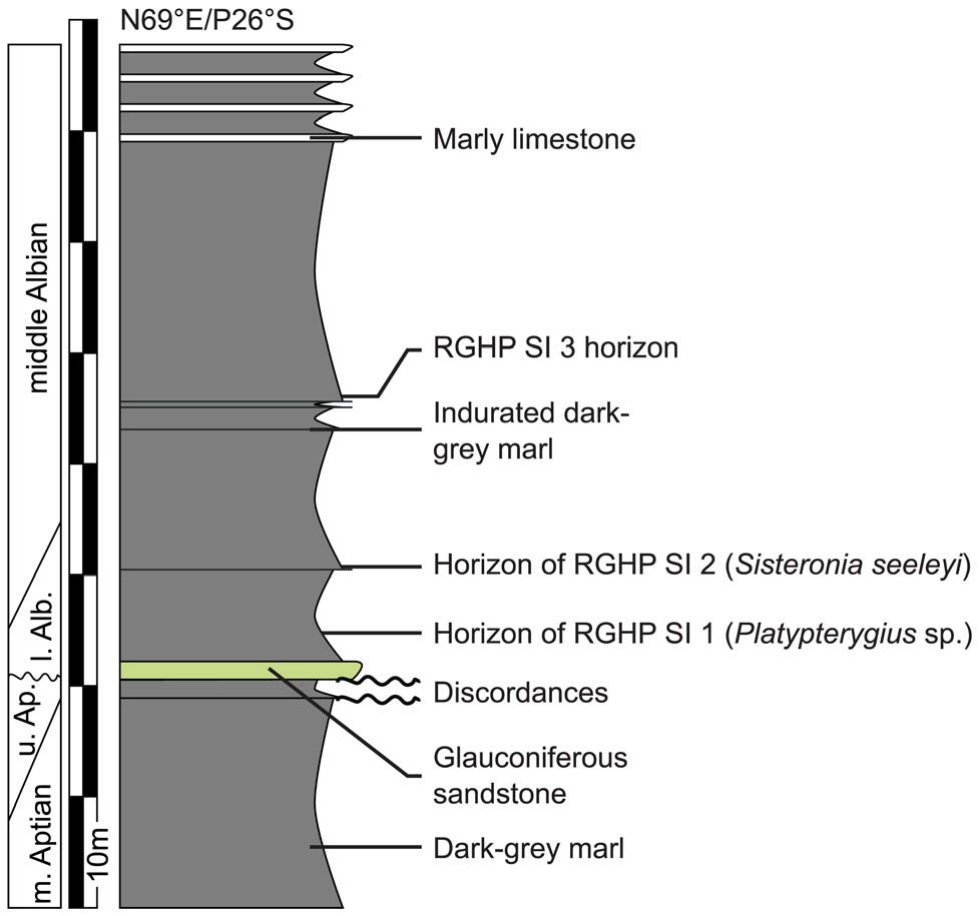
Stratigraphic log of Les Houlettes locality, Sisteron, Alpes de Haute-Provence, France. The position of the stratigraphic boundaries is taken from Bréhéret [59] and personal fieldwork by V.F. and M.G. Abbreviations: Alb, Albian; Ap, Aptian; m., middle; l., lower; u., upper.

**Figure 4:**
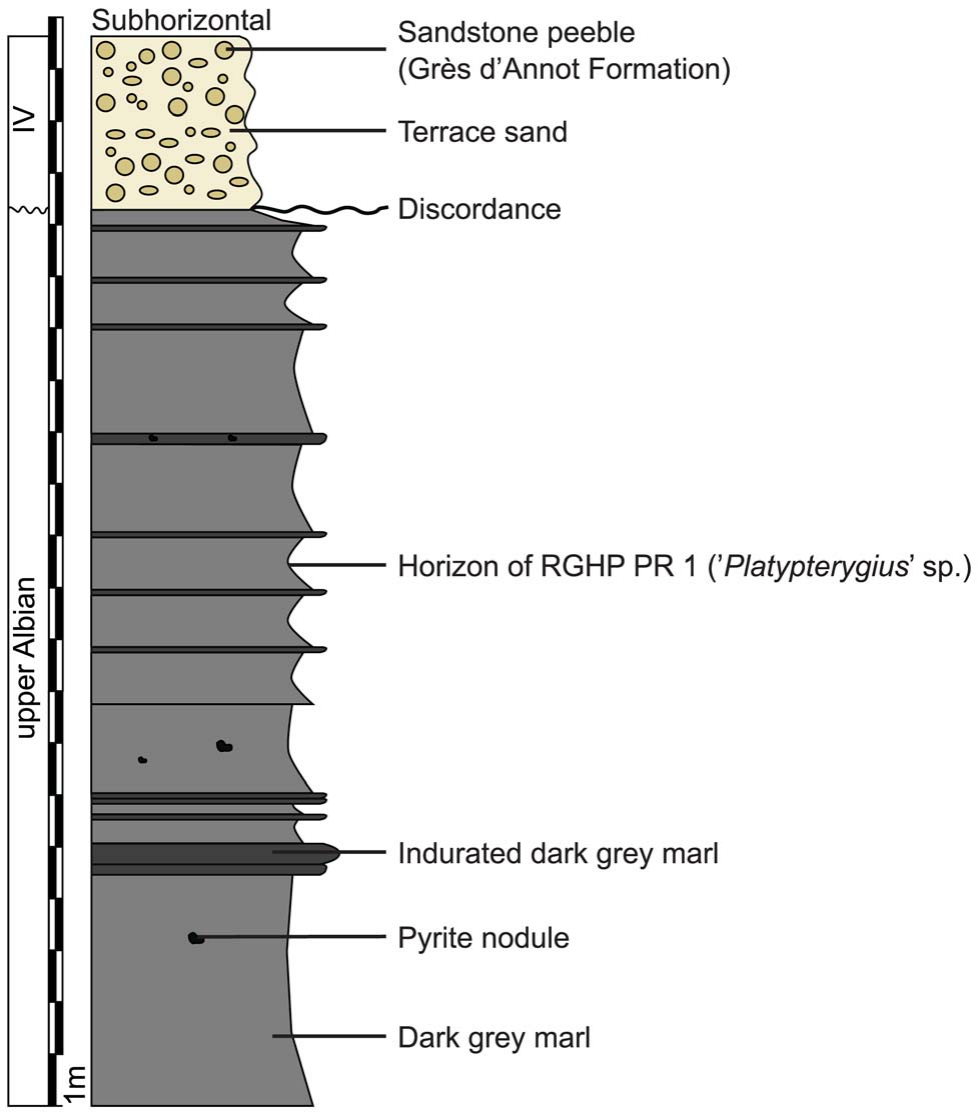
Stratigraphic log at RGHP PR 1's discovery site, Prads-Haute-Bléonne, Alpes de Haute-Provence, France.

The Marnes Bleues Formation is a monotonous succession of grey marls with a significant lateral variation in thickness and local unconformities ([61], [62]; V.F. & M. G., pers. obs.). Several local sandstone and limestone beds interrupt the sequence (e.g. [59], [63]; V.F. & M. G., pers. obs.). Cephalopods are rare in this formation, and the age of the horizon of some specimens is only loosely constrained. In the Sisteron locality, two unconformities disturb the sequence: the upper Aptian lies on the truncated middle Aptian, and the last few meters of lower Albian (or the middle Albian) lie on the truncated upper Aptian via a 20 cm-thick glauconitic sandstone layer [59]; Figure 3). The specimens RGHP SI 1, RGHP SI 2, and RGHP SI 3 were found 2, 8, and 25 meters above the Aptian–Albian discordance, respectively, and are late early to middle Albian in age (Figure 3). In the Prads locality, the upper part of the Marnes Bleues Formation crops out, but a Quaternary terrace reworking sandstone clasts of the Oligocene Grès d'Annot Formation truncates the top of the Marnes Bleues Formation. The specimen RGHP PR 1 was found 6.5 m below the base of the Quaternary terrace and is late Albian in age [64] (Figure 4). Text S4 lists all ichthyosaurs from the Marnes Bleues Formation studied in the present paper.

## Results

### Systematic Paleontology

The asterisk (*) next to referred specimens indicates articulated specimens, others are isolated elements.

Ichthyosauria Blainville, 1835 [65]

Ophthalmosauridae Baur 1887 [66]

Platypterygiinae Arkhangelsky 2001 [67] sensu Fischer et al. [20]

*Sisteronia seeleyi* gen. et sp. nov. urn:lsid:zoobank.org:act:1B87EED5-6C16-49EE-ADC2-67FEB04819F0

Figures 5, 6, 7

**Figure 5:**
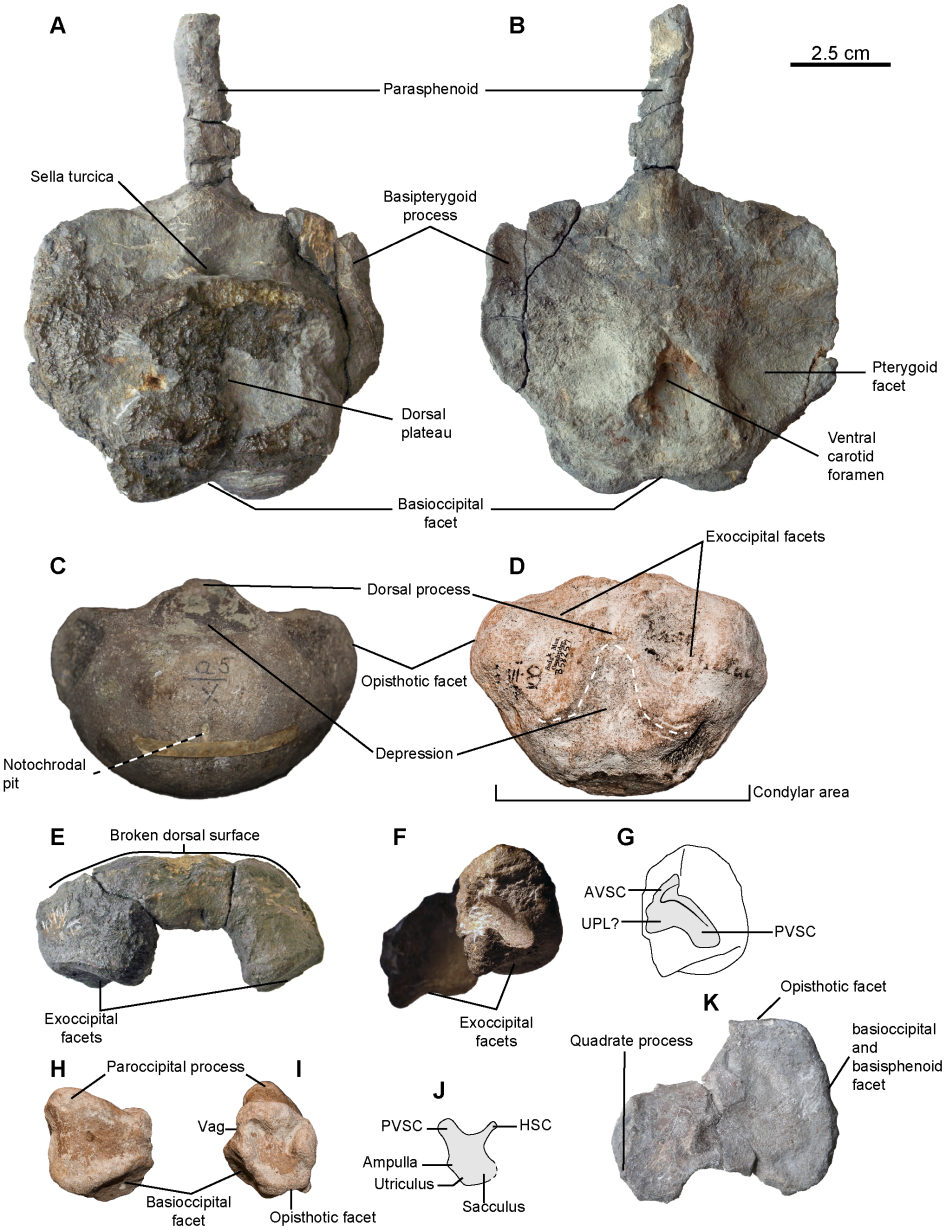
*Sisteronia seeleyi*, basicranium. A, B: basisphenoid (RGHP SI 2) in dorsal (A) and ventral (B) views. C: basioccipital (CAMSM B57943) in posterior view. D: holotype basioccipital (CAMSM B58257_67) in dorsal view. E–G: supraoccipital (RGHP SI 2) in posterior (E) and anterolateral (otic) (F, G) views. H–J: left opisthotic (CAMSM B58257_67) in posterior (H) and anterior (otic) (I, J) views. K: left stapes (RGHP SI 2) in posterior view. Note the extremely reduced (nearly absent) extracondylar area of the basioccipital, a platypterygiine synapomorphy, and the dorsal process posterior to a triangular depression (delineated by the thick dotted line) on the basioccipital, an autapomorphy of *Sisteronia seeleyi*. Abbreviations: AVSC: impression of the anterior vertical semicircular canal of the otic labyrinth; HSC: impression of the horizontal semicircular canal of the otic labyrinth; PVSC: impression of the posterior vertical semicircular canal of the otic labyrinth; UPL: impression of the utricular portion of the otic labyrinth; Vag: vagus foramen.

**Figure 6:**
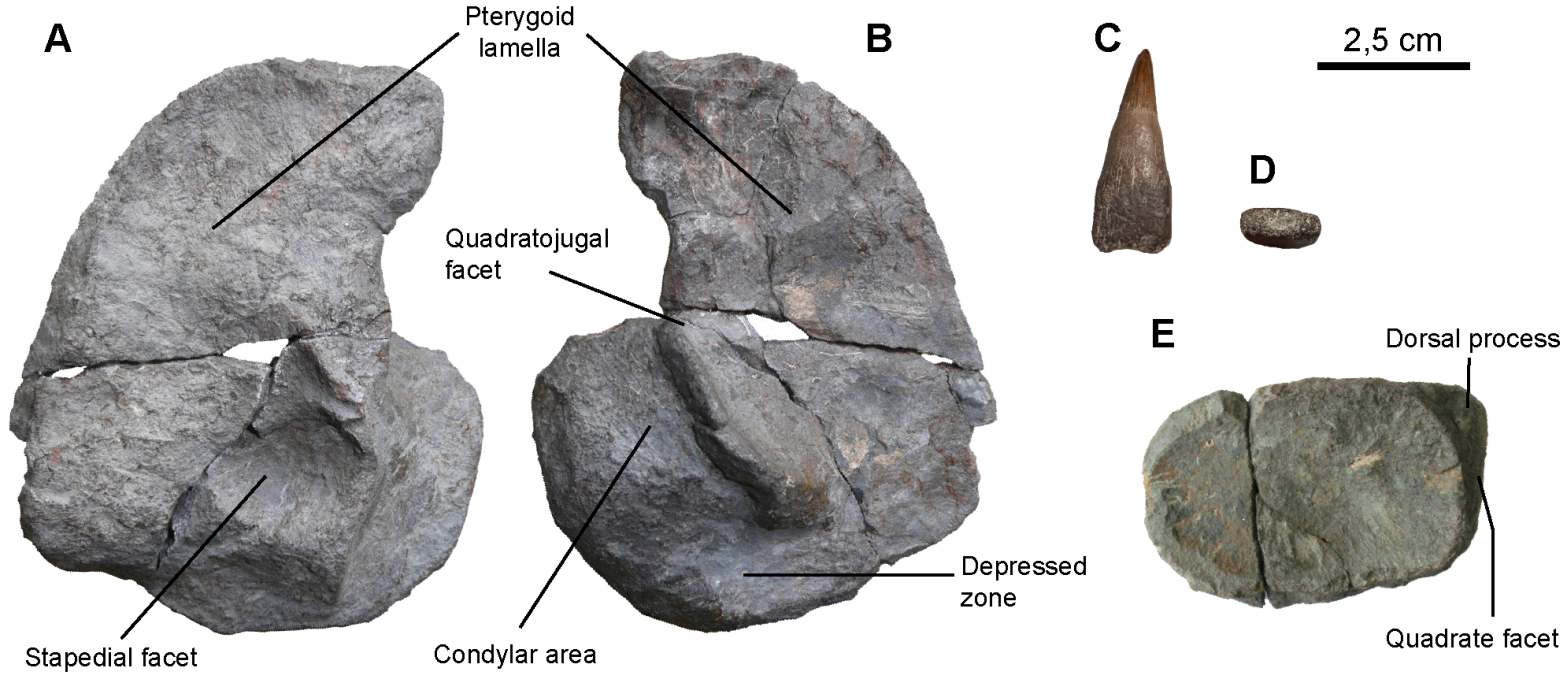
*Sisteronia seeleyi*, quadrate, tooth and articular. A, B: right quadrate (RGHP SI 2) in medial (A) and lateral (B) views. C, D: typical mid-rostrum tooth of *Sisteronia seeleyi* (CAMSM TN1779 partim) in labial view (C) and basal (D) views, showing the markedly rectangular cross-section of the root. E: right articular (RGHP SI 2) in lateral view.

**Figure 7:**
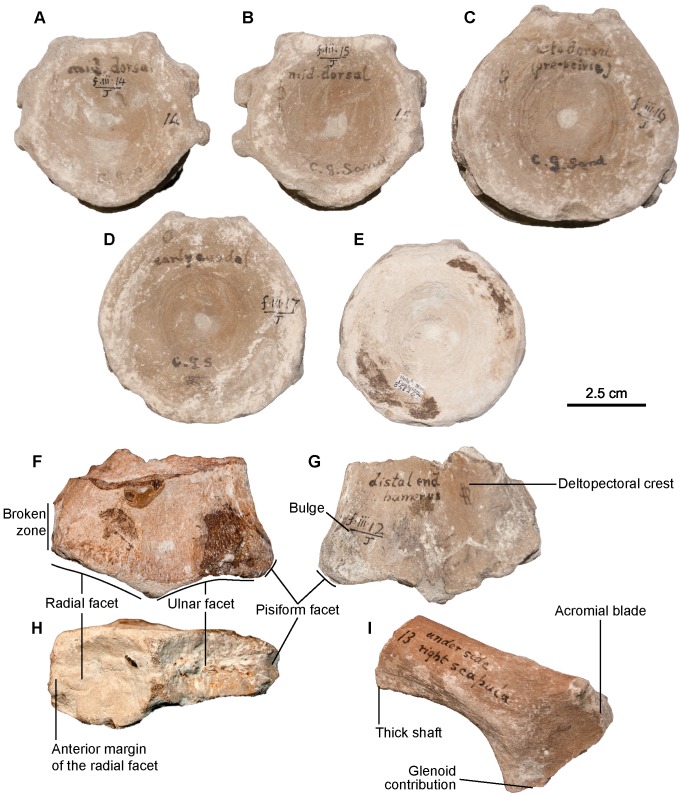
*Sisteronia seeleyi*, axial and shoulder girdle elements of holotype specimen (CAMSM B58257_67). A–E: centra in anterior view. A: cervical centrum. B: anterior thoracic centrum. C: posterior thoracic centrum, close to the sacral region. D, E: anterior caudal centra. F–H: left humerus (CAMSM B58257_67) in dorsal (F), ventral (G), and distal (H) views. Note the presence of a facet for a posterior accessory epipodial element, a feature only found in some platypterygiine ichthyosaurs. I: right scapula in anterior view.

1889 *Ichthyosaurus campylodon* Lydekker [68]: 19 (NHMUK R16)

1889 *I. campylodon*/*Ophthalmosaurus?* Lydekker [68]: 20 (NHMUK 44159)

1889 *I. campylodon*/*Ophthalmosaurus?* Lydekker [68]: 20 (NHMUK 44159a)

2003 Ichthyosauria indet. McGowan & Motani [69]: 27: Figure 37

#### Holotype

CAMSM B58257_67, an incomplete specimen, including partial basicranium, scapula, humerus, and 5 centra from unreworked (chalky) part of the Cambridge Greensand member (early Cenomanian, Late Cretaceous). The basioccipital is fully ossified and the humerus lacks a rugose texture on its shaft, suggesting a mature specimen [70].

#### Referred material from the Cambridge Greensand

CAMSM B57943 (basioccipital); CAMSM B57945 (basioccipital); CAMSM B57948 (basioccipital); CAMSM B57950 (basioccipital); CAMSM B57947 (basioccipital); CAMSM B57941 (basioccipital); CAMSM B57951 (basioccipital); CAMSM B57946 (basioccipital); CAMSM B57956 (basioccipital); CAMSM B57954 (basioccipital); CAMSM B58314 (basioccipital); CAMSM TN1727 partim (basioccipital); CAMSM TN1735 partim (6 basioccipitals); CAMSM TN1739 partim (basioccipital); CAMSM TN1751 partim (6 basioccipitals); CAMSM TN1753 partim (basioccipital); IRSNB GS54 (basioccipital); IRSNB GS61 (basioccipital); LEICT G107.1991 (basioccipital); NHMUK 44159 (basioccipital); NHMUK 44159a (basioccipital); CAMSM B57908 (opisthotic); CAMSM B58077_78 (2 opisthotics); CAMSM TN1753 partim (opisthotic); NHMUK R2348 (opisthotic); IRSNB GS10 (opisthotic); CAMSM B58091 (tooth); CAMSM B58092 (tooth); CAMSM TN1716 partim (numerous teeth); CAMSM TN1778 partim (numerous teeth); CAMSM TN1779 partim (numerous teeth); CAMSM B58390 (tooth); NHMUK R1923 (tooth); IRSNB GS23 (tooth); IRSNB GS24 (tooth); IRSNB GS55 to GS58 (teeth); CAMSM TN1755 partim (humerus); CAMSM TN1757 partim (humerus).

#### Referred material from other deposits

NHMUK R16 partim (teeth, Gault Formation); NHMUK R17 partim (teeth, Gault Formation); NHMUK R2890 partim (opisthotic, Gault Formation); NHMUK 47232 partim (teeth, Gault Formation); RGHP SI 2*, an incomplete skull, containing fragmentary snout and nasals, basioccipital, quadrate, opisthotic, supraoccipital, stapes, teeth from the middle Albian of Sisteron. At least three additional articulated specimens from the middle–late Albian of the Marnes Bleues Formation of the Vocontian Basin are present in the private collection of L. Ebbo [71].

#### Diagnosis

Platypterygiine ophthalmosaurid characterized by the following autapomorphies: basioccipital with raised process on the floor of foramen magnum; opisthotic with nearly absent paroccipital process (as in juvenile ‘*P.*’ *australis* [26]); tooth with gracile crown and root with rectangular cross-section, the labio-lingual length being usually equal to one half of the anteroposterior length (less conspicuous in anterior- and posterior-most teeth).

*Sisteronia seeleyi* is also characterized by the following unique combination of features: elongated anterior process of the maxilla, reaching anteriorly the level of the nasal (unlike in *Aegirosaurus* [72]; *Sveltonectes insolitus* [21]); prominent opisthotic facets on basioccipital (shared with *S. insolitus* [21]); expanded sacculus impression on opisthotic (shared with adult ‘*P.*’ *australis* [11] and *A. densus* [20]); anteroposteriorly shortened quadrate condyle (shared with *O. icenicus* [73] and *S. insolitus* [21]); U-shaped supraoccipital (shared with ‘*P.*’ *australis* [11]; ‘*P.*’ *hercynicus* [18], [74] and *O. natans* [75]); humerus with a facet for a posterior accessory element (shared with ‘*P.*’ *hercynicus* [74], [76]; ‘*P.*’ *americanus* [14]; ‘*P.*’ sp. [16], [77]; ‘*Ophthalmosaurus monocharactus*’ [78]).

#### Stratigraphic range

Early Albian–early Cenomanian (stratum typicum).

#### Geographic range

Eastern England basins (locus typicus), Vocontian Basin, France.

#### Note

As mentioned in the ‘Referred material from other deposits’ section, above, additional articulated specimens from the Albian of the Vocontian Basin are currently held in a private collection. These specimens were studied in the course of V.F.'s PhD thesis [71] and this information is crucial to establish the phylogenetic relationships of *Sisteronia*. Because this material cannot be used for the time being, we refrain from assessing the phylogenetic position of *Sisteronia* in this paper. These data and the phylogenetic placement of *Sisteronia* can be found in V.F.'s thesis [71]. However, as *Sisteronia* possesses numerous synapomorphies of platypterygiine ophthalmosaurids and lacks the synapomorphies of ophthalmosaurine ophthalmosaurids (see Anatomical Descriptions, below), we confidently place this taxon within Platypterygiinae.

### Description

Measurements taken on CAMSM B58257_67 can be found in Table 2.

**Table 2:** Selected measurements on CAMSM B58257_67, holotype of *Sisteronia seeleyi*.

Measurement (mm)	CAMSM B58257_67
Basioccipital height	36.95
Basioccipital width	70.83
Basioccipital length	49.61
humerus distal diameter	68.1
Radial facet length	32.53
Ulnar facet length	31.15
First preserved dorsal centrum height	52.13
First preserved dorsal centrum width	53.03
First preserved dorsal centrum depth	24.24
Last preserved dorsal centrum height	52.65
Last preserved dorsal centrum width	53.34
Last preserved dorsal centrum depth	24.41
First preserved caudal centrum height	63.15
First preserved caudal centrum width	64.17
First preserved caudal centrum depth	23.31
Last preserved caudal centrum height	55.96
Last preserved caudal centrum width	57.26
Last preserved caudal centrum depth	20.99

Measurements are recorded up to the nearest 0.01 mm using a digital caliper.

#### Basioccipital (morphotype 2, see Systematic Paleontology above for a list of all specimens; Figure 5)

The basioccipital is roughly semi-circular in posterior view. As in *Sveltonectes* [21], the basioccipital is wider than high because of the prominence of the bulge-like opisthotic facet, the complete reduction of the extracondylar area ventrally, and the deep exoccipital facets. The extracondylar area is extremely reduced laterally (condyle width = 84.69% of the total width in CAMSM B57943) and invisible ventrally in posterior view, a synapomorphy of platypterygiine ophthalmosaurids [20]. The condyle is oval and not flattened, and the notochordal pit is located ventral to the central point in most specimens. There is no ventral notch, but the ventral surface is flattened. The stapedial facet is not visible. The exoccipital facets are prominent and bordered medially and posteromedially by a prominent ridge. Both ridges meet medially and form a prominent process dividing the floor of foramen magnum in two in the transverse plane. In dorsal view, this ridge is wave-like and W-shaped. This structure appears ontogenetic, because the smaller basioccipitals have a reduced ridge. The anterior surface is flat and vertical, and the notochordal groove is shallow or absent. Two specimens (CAMSM B57948 and CAMSM B57954) have reduced opisthotic facets, a reduced exoccipital ridge, and deep dorsoventral grooves separating the basisphenoid facet from the opisthotic facet, as in ‘*P.*’ *australis* [11]. They are nevertheless closer to the *Sisteronia* morphotype in general shape and are therefore included in this group.

#### Opisthotic (CAMSM B57908; CAMSM B58077_78; CAMSM B58257_67* (holotype); CAMSM TN1753 partim; NHMUK R2348; IRSNB GS10; NHMUK R2890 partim; CAMSM ‘Saxon Cement works Cambridge 1912’; RGHP SI 2*; Figure 5)

The paroccipital process is robust and extremely shortened, unlike that of ophthalmosaurine ichthyosaurs [20], [73], and even shorter than in adult ‘*P.*’ *australis* [11] and ‘*P.*’ *hercynicus* [74], [76] and resembles that of juvenile ‘*P.*’ *australis* [26]. There is no lateral ridge, unlike in *O. icenicus* and *A. densus* [20], [73]. The opisthotic forms two facets medioventrally: a large, rugose, triangular facet facing posteroventrally for the basioccipital and a smaller, roughly triangular facet for the stapes. The stapedial facet is frequently subdivided by a deep anterolateral groove. This deep and narrow groove probably housed the hyomandibular branch of facial (VII) nerve or the glossopharyngeal (IX) nerve [73] and can be extremely complex in some specimens, such as NHMUK R2890, forming lateral spirals. The otic capsule impression has a deep and elongated impression for the horizontal semicircular canal, a wider and shorter impression for the posterior vertical semicircular canal, and a markedly expanded sacculus, as in adult ‘*P.*’ *australis* [11] and the holotype (adult) specimen of *A. densus* [20].

#### Stapes (RGHP SI 2*; Figure 5)

Both stapes are preserved in RGHP SI 2 but crushed along different planes. The shaft is short and robust unlike in *A. densus* [20]. The opisthotic surface forms a marked angle with the basioccipital/basisphenoid facet. There is no evidence for a hyoid process.

#### Supraoccipital (RGHP SI 2*; Figure 5)

The supraoccipital is U-shaped with a ‘squared’ opening for the foramen magnum, similar to the condition in ‘*P.*’ *hercynicus* [18], [74]. The exoccipital facets are trapezoidal, tapering posteriorly, and are markedly concave. Partial otic impressions are preserved in RGHP SI 2; the impression for the posterior vertical semicircular canal is extremely deep. The utriculus (‘utricular portion of labyrinth’ of McGowan [79]) impression is a broad semicircular depression that is confluent with the impression for the posterior vertical semicircular canal dorsolaterally. Unlike in ‘*P.*’ *australis* and *A. densus* [11], [20], the impression for the anterior vertical semicircular canal is markedly reduced in length and depth and is separated from the rest of the otic impression by a lateral ridge.

#### Parabasisphenoid (RGHP SI 2*; Figure 5)

The basipterygoid process is markedly reduced and forms an elongated bulge on the lateral surface of the basisphenoid. It is even more reduced than in *Sveltonectes*, where it forms a small protruding rod-like process [21], but it may be partly due of the strong diagenetic compaction of this bone in RGPH SI 2. The dorsal plateau appears kidney-shaped, as in *S. insolitus* [21] and unlike those of ‘*P.*’ *australis* (hexagonal [11]), *Brachypterygius* (squared [69]), and *O. icenicus* (rounded [73]). The ventral surface of the basisphenoid bears a wide depression for the medial lamella of the pterygoid. The ventral carotid opening is set in the posterior half of the ventral surface. The posterior surface is divided by a deep median cleft, as in many post-Triassic ichthyosaurs (V.F., pers. obs. on NHMUK and CAMSM material). The parasphenoid is completely fused to the basisphenoid in RGHP SI 2, suggesting a mature age [11], although the ontogenetic significance of this feature has been debated recently [26].

#### Quadrate (CAMSM B58257_67*; RGHP SI 2*; Figure 6)

The quadrate is ear-shaped as in most ophthalmosaurids. The medial surface is flat, and the stapedial articular facet is a deep depression bordered posteriorly and ventrally by a bony ridge. There is no evidence for a marked occipital lamella, unlike in *O. icenicus*, ‘*P.*’ *australis* or *S. insolitus* ([11], [21], [73], respectively). The lateral surface is smooth and markedly concave. The short condyle is thick along its whole length, and rapidly tapers anteriorly, as in *O. icenicus* and *S. insolitus* [21], [73]. The ventral surface of the condyle is concave anteriorly and becomes progressively flat posteriorly. The condyle is separated from the pterygoid lamella by a concave area. Similar quadrates occur in the Cambridge Greensand Member (e.g. CAMSM B57988; CAMSM B57989; NHMUK 35272 [two specimens]; IRSNB GS1; IRSNB GS6; IRSNB GS8), but the lack of clear-cut diagnostic feature prevents confident referral of these isolated bones to *Sisteronia seeleyi*; only the quadrates found in articulation with diagnostic elements are referred to the relevant taxa.

#### Pterygoid (RGHP SI 2*)

A fragmentary pterygoid is preserved in RGHP SI 2. The dorsal lamella has a thick base, and the reception pits for the basipterygoid process are unremarkable, unlike in *A. densus* [20].

#### Articular (CAMSM B58257_67*; RGHP SI 2*; Figure 6)

The left articular is preserved. It appears distinct from that of other ichthyosaurs (e.g. *Ichthyosaurus communis* [79], ‘*P.*’ *australis* [11], [26], *O. icenicus* [80]) in being anteroposteriorly elongated (as in *Arthropterygius chrisorum* [81]) and rectangular. It lacks the muscle attachment bulge seen ‘*P.*’ *australis* and *Sveltonectes insolitus* [11], [21].

#### Dentition (morphotype 2; RGPH SI 2*; see Systematic Paleontology above for a complete list of specimens; Figure 6)

The teeth are straight generally much smaller than in other coeval taxa; the crown accounts for half of the total height in most teeth. Anterior and median teeth have a slender, straight, a conical crown with well-expressed apicobasal ridges and a markedly laterally compressed, yet quadrangular root. This is not a diagenetic artifact, because a large number of roots have resorption pits that remain perfectly circular and dozens of similar teeth are found in the Gault Formation and Cambridge Greensand Member. Posterior teeth have smaller and more robust crowns, and squarer root cross section. A smooth acellular cementum ring is present, and the root is smooth and lacks a thick layer of cement, unlike in ‘*Platypterygius*’ [82]. It is worth noting that quite similar teeth are found in a juvenile specimen of ‘*P.*’ *australis* (NHMUK unnumbered). This may indicate close relationship between these two taxa and/or potential heterochronial processes related the tooth development.

#### Centra (CAMSM B58257_67*; Figure 7)

A subtle ventral keel occurs on anterior thoracic centra, giving them a pentagonal shape. These centra have prominent diapophyses and parapophyses; horizontal bony ridges follow these apophyses posteriorly. Sacral and anterior caudal centra are weakly amphicœlous and have a circular outline.

#### Scapula (CAMSM B58257_67*; Figure 7)

The medioventral part of the scapula is dorsoventrally compressed and widely expanded anteroposteriorly, to form the articulation area for the coracoid and the glenoid ventrally, and the acromion process anteriorly. Most of the medial part of the proximal surface is missing, so it is impossible to know if the scapular facet and the acromion process were continuous, as in *Ophthalmosaurus icenicus* [83], *Acamptonectes densus* [20], and *Platypterygius americanus* [84], or separated by a deep notch as in *Sveltonectes* [21]. The dorsal surface of the medial part of the scapula is concave, whereas its ventral surface is flat. The posterior margin of the scapula is markedly curved. Distally, the scapula is thick and rod-like, as in ‘*P.*’ *hercynicus* [74], [76] and unlike *O. icenicus* [80], [85] and *A. densus* [20].

#### Humerus (CAMSM B58257_67*; CAMSM TN1757 partim; Figure 7)

The anterior surface of the shaft is rounded, whereas the posterior blade is acute and bordered by concave areas, giving the humerus a teardrop shape in cross-section. The deltopectoral crest nearly reaches the distal end of the humerus and merges with the ventral edge of the radial facet. Posterodistally, a bulge is present on the ventral side of the humerus, near the ulnar facet as in *Sveltonectes insolitus* [21] (but a dorsal bulge is also present in *Sveltonectes* [V.F., pers. obs.]). The humerus forms at least three distal facets: a large rounded radial facet, a longer (anteroposterior distance) but thinner (dorsoventral distance) ulnar facet, and a small triangular postaxial accessory facet. This condition has only been reported in some taxa referred to as *Platypterygius* (‘*P.*’ *hercynicus* [74]; ‘*P.*’ *americanus* [14]; ‘*P.*’ sp. [16], [77]). All facets are rugose and concave. The anterodistal extremity of the humerus is damaged. Yet, the anterior edge of the radial facet is preserved, and the shape of the anterior surface of the humerus suggests that a facet for an anterior accessory epipodial element was also present.

### Systematic Paleontology

*Platypterygius* Huene 1922 [86]

‘*Platypterygius*’ sp.

Figures 8, 9, 10, 11, 12, 13, 14, 15

**Figure 8:**
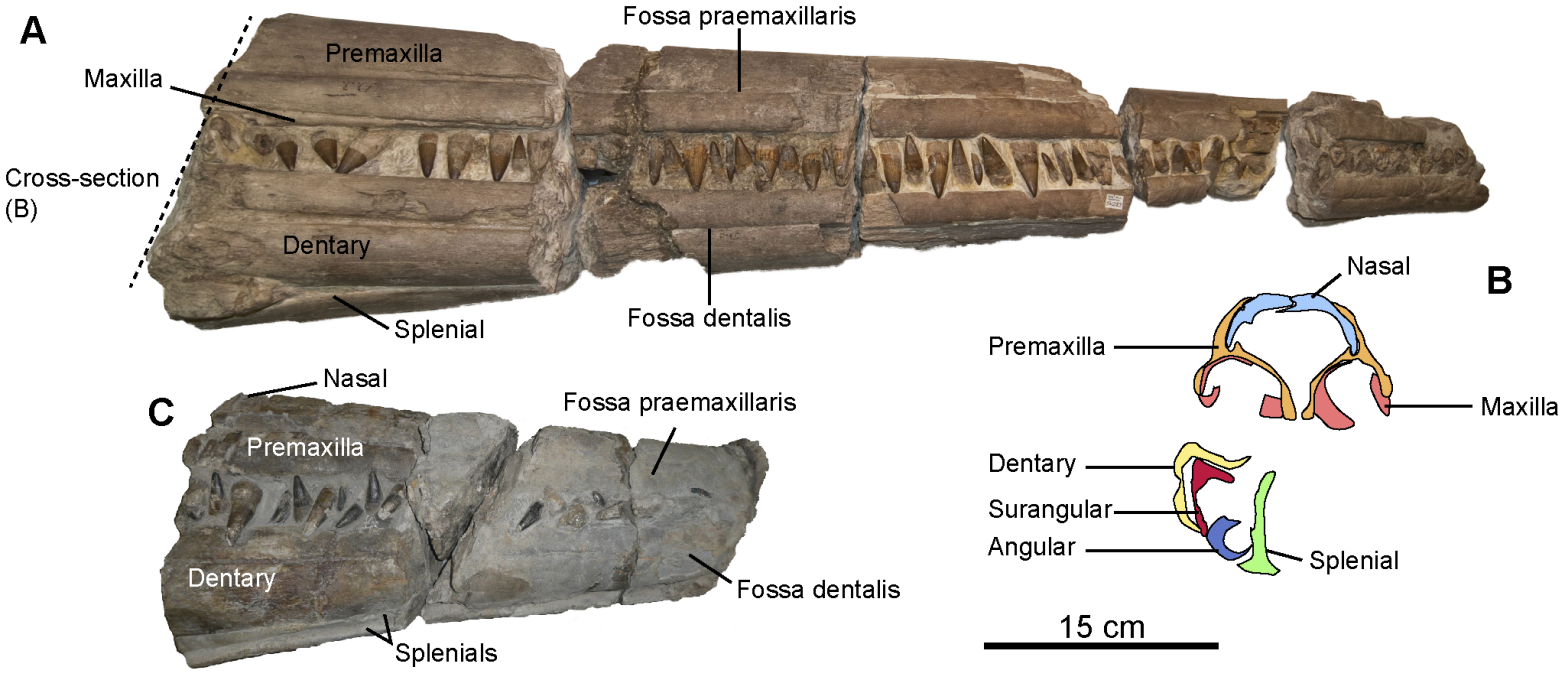
‘*Platypterygius*’ sp., rostra. A: CAMSM TN283, articulated rostrum in right lateral view. The dashed line indicates the plane and position of the cross-section in B. B: posterior-most cross-section of CAMSM TN283, set posterior to the symphysis. C: RGHP PR 1, articulated rostrum in right lateral view.

**Figure 9:**
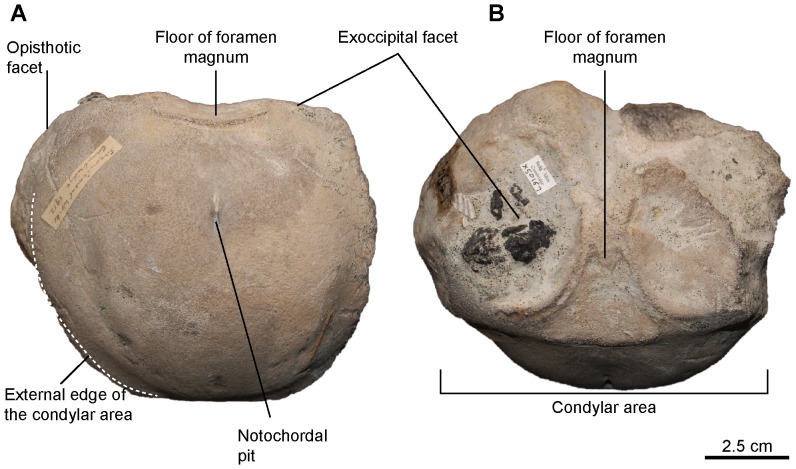
‘*Platypterygius*’ sp., basioccipital (CAMSM X50167) in posterior (A) and dorsal (B) views. Note the extremely reduced extracondylar area, a platypterygiine synapomorphy that appears exaggerated in this taxon.

**Figure 10:**
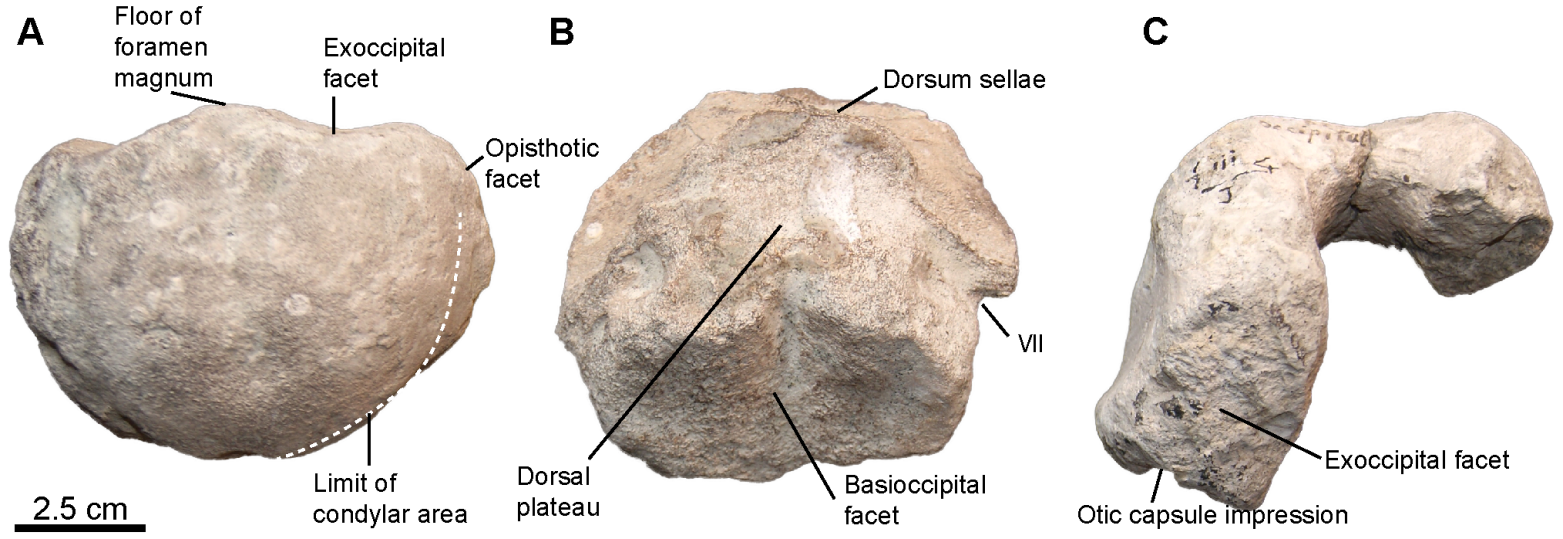
‘*Platypterygius*’ sp., associated basicranium of CAMSM B58250_56. A: basioccipital in posterior view. This basioccipital has a raised floor within the foramen magnum, as in numerous other isolated basioccipitals and ‘*Platypterygius* cf. *kiprijanoffi*’ described by Bardet [71]. B: basisphenoid in dorsal view. C: supraoccipital in posterior view. This specimen also contains a femur (femur morphotype 1). Abbreviations: VII: foramen for the facialis nerve (VII).

**Figure 11:**
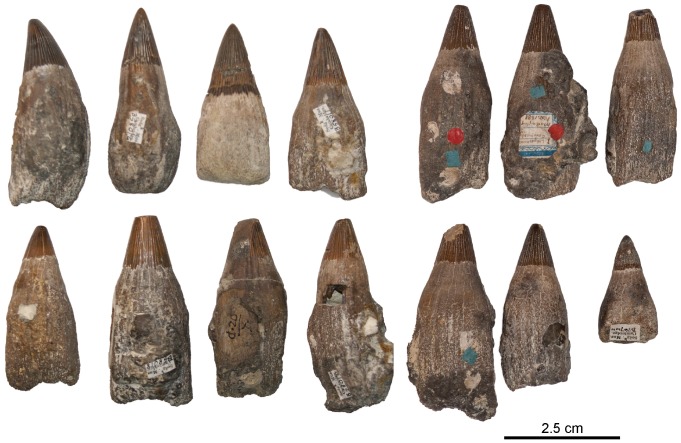
‘*Platypterygius*’ sp., teeth (morphotype 1) of medium size. The eight teeth on the left are isolated teeth grouped within the specimen CAMSM B58010 to 58019, and the six teeth on the right are said to have been found associated (specimen CAMSM B76728_45), but their mode of preservation recalls the reworked part of the Cambridge Greensand Member, making it highly unlikely. Note the bulbous and striated root.

**Figure 12:**
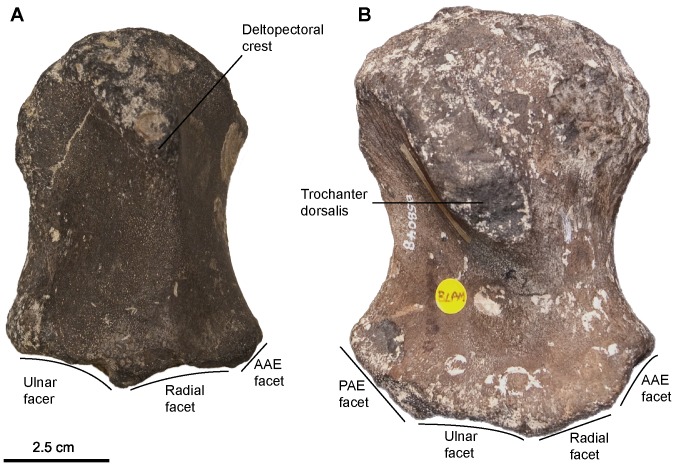
‘*Platypterygius*’ sp., humerus morphotypes. A: Left humerus (morphotype 1) in ventral view (CAMSM TN1757 partim). Note the large radial and ulnar facets set on the same plane. B: Right humerus (morphotype 4) in dorsal view (CAMSM B58048). Note the large four distal facets including one for an anterior and a posterior accessory epipodial element. Abbreviation: AAE, anterior accessory epipodial element; PAE: posterior accessory epipodial element.

**Figure 13:**
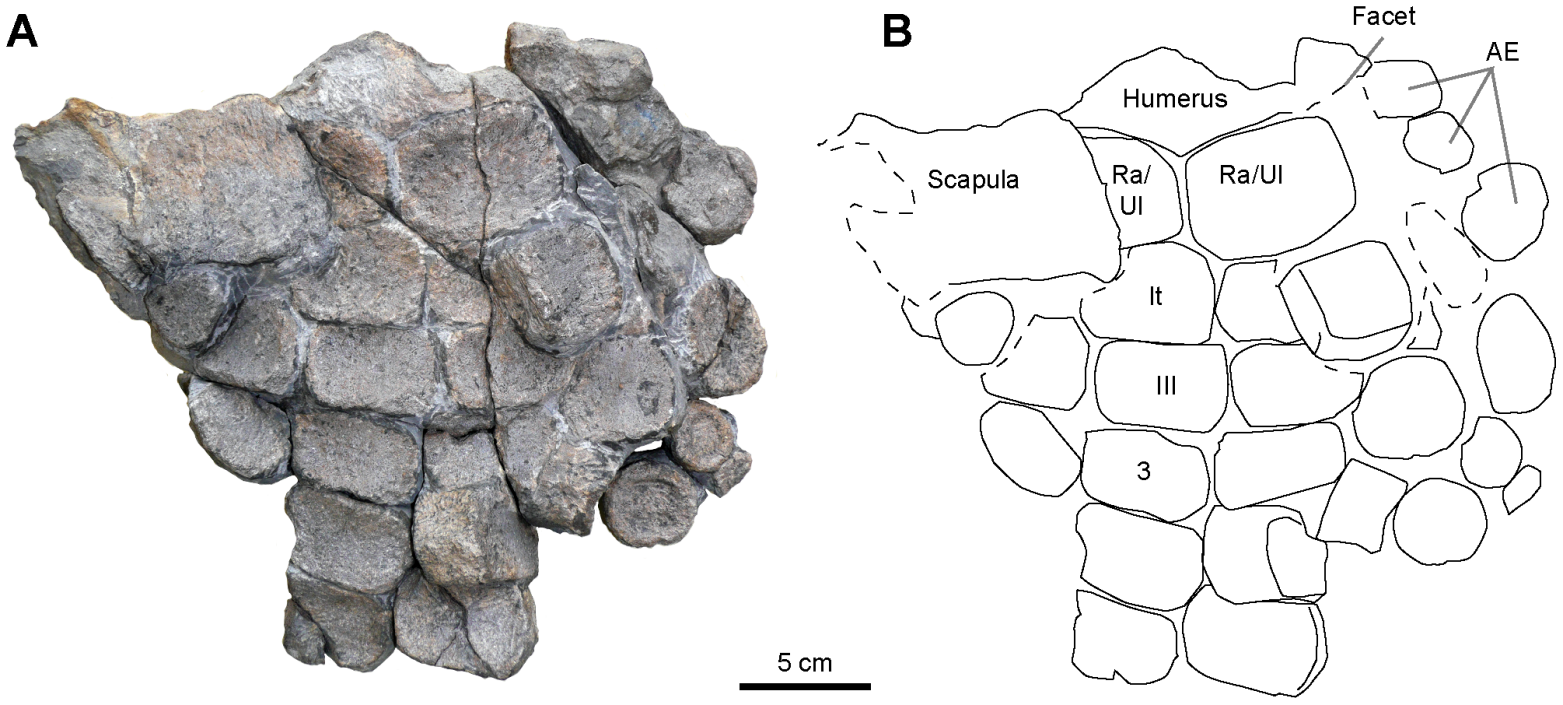
‘*Platypterygius*’ sp., articulated partial forefin (RGHP PR 1), photograph (A) and interpretation (B). The remains are insufficient to characterize which side this forefin is from. Abbreviation: AE: accessory elements; III: carpal 3; It: intermedium; Ra: radius; Ul: ulna; 3: metacarpal 3.

**Figure 14:**
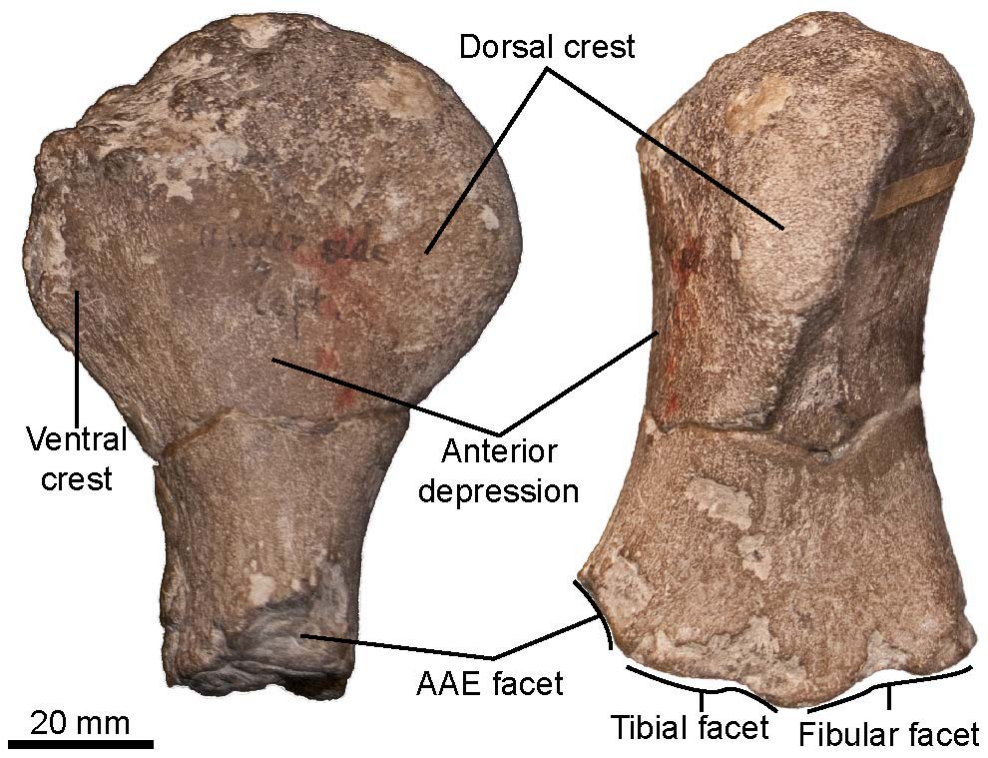
‘*Platypterygius*’ sp., left femur (CAMSM B58058) in anterior (left) and dorsal (right) views. Abbreviation: AAE: anterior accessory epipodial element.

**Figure 15:**
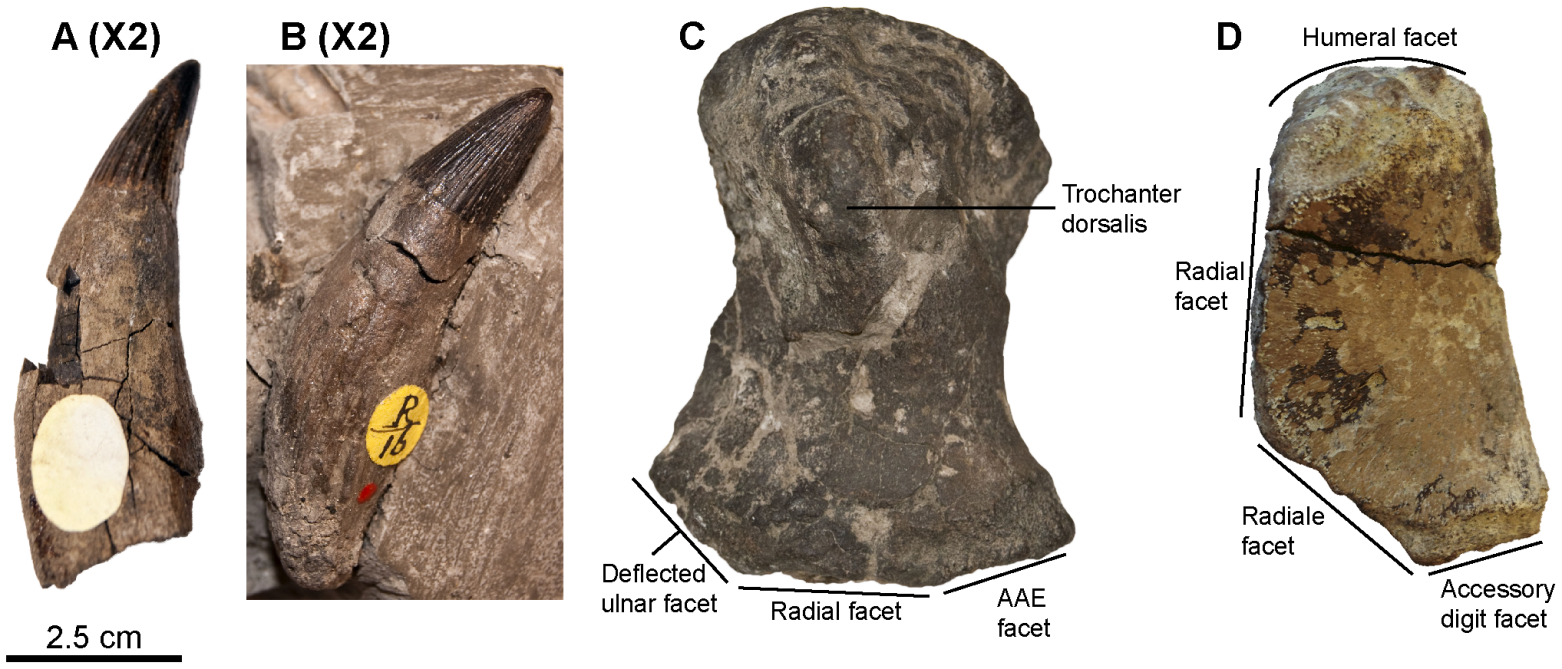
Indeterminate ophthalmosaurine ophthalmosaurids from the Gault Formation and Cambridge Greensand Member. A, B: Teeth (NHMUK R16 partim; magnified two times with respect to other elements); C: right humerus in dorsal view (CAMSM TN1755 partim), note the posterolaterally deflected ulnar facet, an ophthalmosaurine synapomorphy; D: anterior accessory epipodial element in dorsal view (IRSNB GS10). Abbreviation: AAE: anterior accessory epipodial element.

1869 *Ichthyosaurus platymerus* Seeley [50]: xvii

1869 *Ichthyosaurus bonneyi* Seeley [50]: xvii

1889 *Ophthalmosaurus* (?) *cantabrigiensis* Lydekker [68]: 9 (NHMUK 35310)

1889 *Ichthyosaurus campylodon* Lydekker [68]: 17 (NHMUK 47235)

1889 *Ichthyosaurus campylodon* Lydekker [68]: 18 (NHMUK 35254)

1889 *Ichthyosaurus campylodon* Lydekker [68]: 18 (NHMUK 47265)

1889 *Ichthyosaurus campylodon* Lydekker [68]: 18 (NHMUK 30253_4)

1889 *Ichthyosaurus campylodon* Lydekker [68]: 18 (NHMUK 32242)

1889 *Ichthyosaurus campylodon* Lydekker [68]: 18 (NHMUK 35434)

1889 *Ichthyosaurus campylodon* Lydekker [68]: 18 (NHMUK 40358)

1889 *Ichthyosaurus campylodon* Lydekker [68]: 18 (NHMUK 41896)

1889 *Ichthyosaurus campylodon* Lydekker [68]: 18 (NHMUK 32406)

1889 *Ichthyosaurus campylodon* Lydekker [68]: 18 (NHMUK 40095)

1889 *Ichthyosaurus campylodon* Lydekker [68]: 18 (NHMUK 46381)

1889 *Ichthyosaurus campylodon* Lydekker [68]: 18 (NHMUK 47269)

1889 *Ichthyosaurus campylodon* Lydekker [68]: 18 (NHMUK 47235)

1889 *Ichthyosaurus campylodon* Lydekker [68]: 19 (NHMUK R16)

1889 *Ichthyosaurus campylodon* Lydekker [68]: 19 (NHMUK 47270)

1889 *Ichthyosaurus campylodon* Lydekker [68]: 19 (NHMUK 36318)

1889 *Ichthyosaurus campylodon* Lydekker [68]: 19 (NHMUK 36384)

1889 *I. campylodon*/*Ophthalmosaurus?* Lydekker [68]: 20 (NHMUK 35323)

1960 *Myopterygius campylodon* Delair [87]: 69 (CAMSM B5839_82)

1960 *Myopterygius campylodon* Delair [87]: 70 (NHMUK 40095)

#### Referred material from the Cambridge Greensand Member

CAMSM TN283* (rostrum and associated 112 teeth); CAMSM B42404_20* (basioccipital, centra); CAMSM B57939 (basioccipital); CAMSM B57940 (basioccipital); CAMSM B57944 (basioccipital); CAMSM B57959_60* (basioccipital, atlas-axis); CAMSM B58250_56* (quadrate, basioccipital, basisphenoid, supraoccipital, femur); CAMSM B75735 (basioccipital); CAMSM X50161 (basioccipital); CAMSM X50168 (basioccipital); CAMSM X50169 (basioccipital); CAMSM TN1729 partim (basioccipital); CAMSM TN1754 partim (basioccipital); CAMSM TN1755 partim (2 basioccipitals); NHMUK 35323 (basioccipital); several dozens of teeth, including CAMSM B57996_58009, CAMSM B58010 to B58027, CAMSM B58305_13; CAMSM B58379_87, CAMSM B76728_45, CAMSM TN1716 partim, CAMSM TN1778 partim; CAMSM TN1779 partim, NHMUK R625, NHMUK R133b partim, NHMUK R2336 partim (2 teeth), NHMUK 28110 partim, NHMUK 30253 partim, NHMUK 30254 (4 teeth), NHMUK 32406 partim, NHMUK 33242, NHMUK 35254 partim, NHMUK 35432_5, NHMUK 40358, NHMUK 41896, NHMUK 46381, NHMUK 47265_66* (teeth, centra), NHMUK 47269, IRSNB GS21, IRSNB GS25 to GS28, IRSNB GS32 to GS50, IRSNB GS53, IRSNB GS62; CAMSM B97401 partim (humerus morphotype 1); CAMSM B57987 (humerus morphotype 1); CAMSM B58043 (humerus morphotype 4); CAMSM B58048 (humerus morphotype 4); CAMSM B58056 (humerus morphotype 1); CAMSM B58057 (humerus morphotype 1; holotype of *Ichthyosaurus platymerus*); CAMSM B97401 partim (humerus morphotype 1); CAMSM TN1734 partim (humerus morphotype 4); CAMSM TN1751 partim (humerus morphotype 1); CAMSM TN1753 partim (one (humerus morphotype 1and one (humerus morphotype 4); CAMSM TN1757 partim (humerus morphotype 4); NHMUK R2342 partim (two humerus morphotype 4); CAMSM B58058 (femur); CAMSM B58060 (femur); CAMSM B58062 (femur; holotype of *Ichthyosaurus bonneyi*); CAMSM B58063_4 (femur); CAMSM B58361 (femur); CAMSM TN1749 partim (femur); CAMSM TN1748 partim (femur); CAMSM TN1757 partim (2 femora); NHMUK R23412 partim (femur); NHMUK R3510 (femur).

#### Additional material from other deposits

RGHP SI 1* (basioccipital, centra); RGHP PR 1* (rostrum, teeth, scapula, humerus, forefin); NHMUK 40095 (tooth, Gault Formation); NHMUK 47235* (a dentary and 12 teeth); NHMUK R16 partim (tooth, Gault Formation); NHMUK R2890 (tooth, Gault Formation); NHMUK 36318 (teeth, Gault Formation); NHMUK 36384 (teeth, Gault Formation); NHMUK 47235 (teeth, Gault Formation); NHMUK 47270 (tooth, Gault Formation).

#### Occurrence

Late Albian of Gault Formation (UK), middle and late Albian of Marnes Bleues (France), earliest Cenomanian of the Cambridge Greensand Member (UK).

#### Note

This taxon corresponds to most of the material previously referred to as ‘*Platypterygius*’ and ‘*P. campylodon*’ from the Albian–earliest Cenomanian of Europe. *Platypterygius campylodon* was erected on material from the chalk [88], [89] and has a complex taxonomic history [2], [69], [90]; personal observations on the syntypes by V.F. suggest that this material is diagnostic, but appears distinct from the abundant material in lower stratigraphic levels (the Gault Formation, the Upper Greensand Formation and the Cambridge Greensand Member). Therefore, pending a thorough reassessment of the species nested within *Platypterygius*, the material outside the chalk cannot be referred to ‘*P.*’ *campylodon* unambiguously. Because phylogenetic and morphological analyses (e.g. [18], [20], [22]) indicate that *Platypterygius*, as currently defined, is a waste-basket, polyphyletic taxon, it cannot be used unambiguously at the moment either. Therefore, we opt here for a generic rank taxon, ‘*Platypterygius*’ sp., that groups large platypterygiine specimens that share similarities with ‘*P.*’ *hercynicus* and ‘*P.*’ *campylodon* sensu stricto. Detailed analysis of articulated material, such as the specimen described by Bardet [90], could further elucidate the anatomy, relationships and the taxonomic diversity of these large platypterygiine taxa from the Albian–Cenomanian of Eurasia. Because ‘*Platypterygius*’ sp. is based on numerous disarticulated remains, many of which are isolated bones, this taxon will not be counted as a distinct taxon in timebins where ‘*P.*’ *hercynicus* and/or ‘*P.*’ *campylodon* sensu stricto occur.

### Description

#### Premaxilla (CAMSM TN283*; RGHP PR 1*; Figure 8)

The premaxilla is elongated and is impossible to distinguish from the dentary in the anterior-most part. Fossa praemaxillaris is shallow and ends anteriorly as a series of deep foramina. A network of very shallow grooves departing from these foramina textures the lateral surface of the very tip of the snout. The dental groove is deep, and the lingual wall is higher than the labial wall. Both these walls are greatly thickened ventrally. An intraosseous channel similar to the Meckelian canal of the dentary is present anteriorly.

#### Nasal (CAMSM TN283*; RGHP PR 1*; Figure 8)

The nasal starts anteriorly as a thin plate covering the internal surface of the premaxilla, before emerging and forming the dorsomedial surface of the rostrum. Unusually, the nasals interlock in a tongue-in-a-groove fashion in CAMSM TN283.

#### Maxilla (CAMSM TN283*; Figure 8)

The maxilla is elongated and low. It emerges at the same level as the nasal in CAMSM TN283, thus differing from Kiprijanoff's ‘*P.*’ *campylodon* material [91], and more posteriorly in RGHP PR 1, where there is no trace of a maxilla even in the posterior-most section of the rostrum. The medial part of the maxilla forms a very thick lingual wall posteriorly.

#### Basioccipital (morphotype 1; see Systematic Paleontology above for a complete list of specimens; Figures 9, 10)

The basioccipital is spherical and usually of large size (except in CAMSM B57944). The condyle is large and markedly rounded and its peripheral edge is slightly flared. The median notochordal pit is teardrop-shaped and is located in the upper half of the condyle. It is sometimes accompanied by a narrow and shallow dorsoventral groove. The extracondylar area is extremely reduced, both ventrally and laterally (condyle width = 87.92% total width in CAMSM X50161). There is no ventral notch, and the extracondylar area is an oblique flat-topped ridge on the lateral surface of the basioccipital. There is no distinctive stapedial facet, and the opisthotic facet is a plateau the barely stands out (if at all) from the extracondylar area, unlike in *Sisteronia seeleyi*. The exoccipital facets are large, oval, slightly concave and lay directly on the body of the basioccipital, unlike in *Sisteronia seeleyi*, where the exoccipital facets are raised. The exoccipital facets are separated medially by a smooth and concave groove forming the base of foramen magnum. This groove is flattened in its middle part and then deepens anteriorly, forming a deep groove housing the notochordal pit anteriorly. The anterior surface is oblique and flat or slightly convex.

This basioccipital morphotype belongs to a platypterygiine ophthalmosaurid, as indicated by the extremely reduced extracondylar area and lack of a peripheral groove around the condyle [20]. Within this clade, only the basioccipital of the genus *Platypterygius* is characterized by a reduction of the opisthotic facets, giving the basioccipital a perfectly circular shape in posterior view [11], [90]. In some specimens (e.g. CAMSM B58250_56*), the floor of the foramen magnum is raised and appears very similar to that of ‘*Platypterygius* cf. *kiprijanoffi*’ described by Bardet [90]. Others (e.g. CAMSM X50167) are concave, as in ‘*P.*’ *hercynicus* [76], but not as much as in ‘*P.*’ *australis* [11].

#### Basisphenoid (CAMSM B58250_56*; Figure 10)

The only basisphenoid associated with diagnostic material is incomplete and sheared. The posterior surface is kidney-shaped and slightly concave, with a deep notochordal groove, matching that of the corresponding basioccipital. The ventral carotid foramen is set at the center point. The basipterygoid processes are not preserved.

#### Supraoccipital (CAMSM B58250_56*; Figure 10)

The supraoccipital is markedly U-shaped, as in ‘*P.*’ *australis* [11], ‘*P.*’ *hercynicus* [18], [76], and *O. natans* [83]. The dorsomedial rod is oval in cross-section. Ventrolaterally, the supraoccipital forms an anteroposteriorly-expanded, brick-like exoccipital process. The facet for the exoccipital is flat, rectangular and posteroventrally facing. The anteroventral facet is set at a right angle to the exoccipital facet and bears an impression for the otic capsule, probably the posterior vertical semi-circular canal. This condition differs from ‘*P.*’ *australis* [11], where a T-shaped impression housed the utricle as well. Unlike in ‘*P.*’ *australis* [11], the internal walls of the supraoccipital are smooth and do not bear any foramen.

#### Dentary (CAMSM TN283*; RGHP PR 1*; Figure 8)

The dentary closely resembles the premaxilla, including the shape of the lateral fossa. The lingual wall of the dental groove is also higher than the labial wall.

#### Rest of the mandible (CAMSM TN283*; RGHP PR 1*; Figure 8)

The splenial is the first bone to emerge from the rostrum. It starts anteriorly as a very thin pike of bone, before progressively forming the medial wall of the mandible posteriorly. The angular is long and crescentic in cross-section. It emerges at the level of the symphysis in CAMSM TN283. The surangular is boomerang-shaped in cross-section and emerges≈50 mm after the angular in CAMSM TN283.

#### Dentition (morphotype 1: see Systematic Paleontology above for a complete list of specimens; Figure 11)

The teeth are usually large; the height of the teeth from the middle part of the snout frequently exceeds 5 cm. The crown is conical, straight, robust, and bears numerous deep apicobasal striations. The apex possesses a pitted texture, as described in ‘*P.*’ *hercynicus* [18] and large/adult *Aegirosaurus* [1]. The angle formed by the crown is wide, usually around 30° (but can reach 37° in some teeth of CAMSM B58010_27). Wide and smooth apicobasal ridges texture the acellular cementum ring. This texture is usually restricted on its apical third, but can cover the whole surface in large teeth. The root is markedly thickened with respect to the acellular cementum ring, and its cross-section is squared. Deep apicobasal ridges occur on the root surface, especially in large teeth. As in all ichthyosaurs, there is a considerable degree of dental variation along the rostrum: anterior teeth are rather smaller, slender, and have a straighter crown whereas posterior teeth are smaller and bulkier, with relatively large recurved crown and short but wide roots with a rounded cross-section.

The squared root in cross-section indicates these teeth belong to a platypterygiine ophthalmosaurid [20]. The general morphology of this tooth morphotype, with bulbous roots, robust crowns and numerous apicobasal ridges on crown, acellular cementum ring and root is typical for the platypterygiine genus ‘*Platypterygius*’ (e.g. [18], [82], [92]; V.F., pers. obs.), commonly found in Albian-Cenomanian sediments of western Europe [18], [90], [93], [94]. Given the complex and nebulous taxonomy of that genus [18], this tooth morphotype is assigned to ‘*Platypterygius*’ sp.

#### Centra (CAMSM B4204_20*; RGHP SI 1*)

The height/length ratio is nearly invariable, and close to 2.1. CAMSM B4204_20* contains some of the biggest Cretaceous centra ever reported (up to 240 mm in height).

#### Scapula (RGHP PR 1*)

The scapula is thick proximally, unlike in *Sisteronia seeleyi* and ophthalmosaurines [20]. The acromial region is not preserved, preventing detailed comparison with other ophthalmosaurids.

#### Humerus (morphotypes 1 and 4; see Systematic Paleontology above for a complete list of specimens; Figure 12)

We refer two distinct humerus morphotypes to ‘*Platypterygius*’ sp. The first morphotype contains usually large and stout humeri with thick trochanters, unlike the slender trochanters of *Sisteronia seeleyi*. In proximal view, this gives the humerus a marked rectangular shape. Both trochanters do not vanish before mid-length. Distally, the humerus possesses two large facets for the radius and the ulna that are parallel to sagittal plane, unlike in coeval ophthalmosaurines (see below). These facets are oval, flattened (unlike *S. insolitus* [21]), equal in length, and parallel to the sagittal plane (unlike ophthalmosaurines [20]). In some specimens a small and flattened facet for an anterior accessory element occurs at the extremity of an anterodistal process of the humerus. The diminutive size of the facet and the absence of other differences within that morphotype suggest the absence/presence of this facet is variable at the intraspecific level or related to ontogeny, although the possibility that this could represent two distinct species cannot be dismissed.

Humeri belonging to the second ‘*Platypterygius*’ sp. morphotype (humerus morphotype 4) have a high, usually short, and markedly oblique trochanter dorsalis (restricted to the proximal half of the humerus), as in some specimens of the ophthalmosaurine morphotype. The deltopectoral crest is high and forms a distal shallow ridge that merge with the ventral edge of the radial facet. Both trochanters are bordered by concave areas and give the proximal surface a concave parallelogram shape. The anterior edge of the humerus is rounded, whereas the posterior edge forms a very acute trailing blade, as in *Sisteronia seeleyi*. Unusually, this posterior edge is ‘trochanter-like’, being bordered by concave areas and thickening proximally to form a bulge on posterior end of the glenoid surface. The humerus possesses four distal facets, including two facets for accessory zeugopodial elements: one anteriorly and one posteriorly. Unusually, the posterior accessory facet is large, sometimes larger than the radial facet and faces posterodistally. The anterior accessory facet is the smallest; it is concave, roughly triangular, and faces anterodistally.

The size, stoutness and distal architecture of these humeri correspond to those reported in taxa currently referred to as *Platypterygius* [14], [16], [76], [77]. The humerus morphotype 1 presents a combination of features (large trochanters; large, flat and oval radial and ulnar facet parallel to the sagittal plane; small to absent anterior accessory facet) that is only found in taxa currently referred to as *Platypterygius* from the ‘middle’ Cretaceous of Europe: ‘*P.*’ *campylodon* [91] and ‘*P.*’ *platydactylus* [95], although ‘*P*’. *australis* possesses many similarities with these forms too [13]. The large four distal facets of the humerus morphotype 4 is a feature only found in some Aptian–Albian taxa currently referred to as *Platypterygius* as well: ‘*P.*’ *hercynicus* [74], [76], and ‘*P.*’ sp. from North America [16], [77]. Accordingly, we refer both morphotypes to ‘*Platypterygius*’ sp., but these morphotypes are likely to represent two distinct species.

#### Manus (RGHP PR 1; Figure 13)

The manus is composed of tightly packed rectangular elements, as is typical for most platypterygiine ophthalmosaurids [20]. The manus architecture appears longipinnate (i.e. with a single digit arising from the intermedium) as in most species referred to as *Platypterygius* [2], [13], [14], [74], [76], [84], [95], [96], *Sisteronia* (V.F. pers. obs. on uncurated material from southeastern France), and probably *Arthropterygius* [81], [97].

#### Femur (morphotype 1; see Systematic Paleontology above for a complete list of specimens; Figure 14)

As in *Sveltonectes insolitus* [21], the dorsal and ventral trochanter of the femur are very high and their morphology matches that of the humeri of ophthalmosaurids, by having a high, plate-like, and oblique dorsal trochanter separated from the slightly thicker ventral trochanter by a flattened area anteriorly. Both trochanters vanish at mid-length. The anterior surface is large and flat, and the posterior edge is rounded, giving the capitulum a rounded triangular shape in proximal or cross-section view. Distally, the femur forms three facets, as in many platypterygiines such as *Maiaspondylus* [22], ‘*P.*’ *americanus* [14], ‘*P.*’ *australis* [13] and ‘*P.*’ *hercynicus* [76]. However, the extra facet is small, triangular and for an anterior accessory element. This condition has only been described in ‘*P.*’ *australis* [13]: the other taxa have an extra facet either for a posterior accessory epipodial element or for the astragalus. The fibular facet is triangular and faces posterodistally. The square-shaped tibial facet is the largest and faces anterodistally.

Out of the several femora morphotypes recognized in the Cambridge Greensand member, only one can be attributed to ‘*Platypterygius*’ sp. with confidence, thanks to an articulated specimen (CAMSM B58250_56) from the upper (chalky) part of the Cambridge Greensand Member. Moreover, similarly large and elongated femora with large trochanters, slightly rounded capitulum and three distal facets are only known in ‘*P.*’ *hercynicus* [76] and ‘*P.*’ *australis* [13].

### Systematic Paleontology

Platypterygiinae indet.

1869 *Ichthyosaurus angustidens* Seeley [50]: 3

1869 *Ichthyosaurus bonneyi* Seeley [50] : xvii

1869 *Ichthyosaurus platymerus* Seeley [50] : xvii

#### Note

As noted by Lydekker [98] and McGowan & Motani [69], Seeley [50] proposed the names *Ichthyosaurus bonneyi*, *I. doughtyi*, *I. platymerus* and *I. angustidens* without a formal description or figure, making these taxa nomina nuda. However, we found the holotype specimens for each of these taxa in the CAMSM. Each were placed in a single box and clearly marked as being type specimens. This allows comparison of these taxa with the rest of the Albian record. Given the uncertain future of *Platypterygius* and its species [18], these taxa may therefore have priority over more recent ones, should they be found to belong to the same taxon. Accordingly, these taxa are regarded as nomina inquirenda, even if this. The holotypes of *I. angustidens* (CAMSM B20643, a partial tooth from the Lower Chalk of Hunstanton), *Ichthyosaurus bonneyi* (CAMSM B58062, a femur from the Cambridge Greensand Member), and *I. platymerus* (CAMSM B58057, a humerus from the Cambridge Greensand Member) resemble ‘*Platypterygius*’ sp. However, given the numerous issues inherent to *Platypterygius*,, these species are considered as an indeterminate platypterygiine instead of ‘*Platypterygius*’ sp. for the moment, pending a thorough reassessment of this genus.

### Systematic Paleontology

Ophthalmosaurinae Baur 1887 [66] sensu Fischer et al. [20]

Ophthalmosaurinae indet.

Figure 15

1888 *Ophthalmosaurus cantabrigiensis* Lydekker [98]: 310

1889 *Ophthalmosaurus* (?) *cantabrigiensis* Lydekker [68]: 9 (NHMUK 35348)

1889 *Ichthyosaurus campylodon* Lydekker [68]: 19 (NHMUK R16)

2003 *Brachypterygius cantabrigiensis* McGowan & Motani [69]: 34: Figure48

#### Referred material from the Cambridge Greensand Member

NHMUK 32406 partim (tooth); NHMUK R16 partim (tooth); NHMUK 47268 (5 teeth); CAMSM B58042 (humerus); CAMSM B58045 (humerus); CAMSM B58050 (humerus); CAMSM B58053 (humerus); CAMSM B58055 (humerus); CAMSM TN1727 partim (humerus); CAMSM TN1755 partim (2 humeri); IRSNB GS3 (humerus); LEICT G65.1991 (humerus); NHMUK R2343 (3 humeri); NHMUK R4513 (2 humeri); NHMUK 35348 (humerus); NHMUK 43989 (humerus, holotype of *Brachypterygius cantabrigiensis*); IRSNB GS60 (anterior accessory epipodial element).

#### Referred material from other deposits

NHMUK R16 partim (teeth, Gault Formation); NHMUK R17 partim (teeth, Gault Formation).

#### Note

Additionally, Fischer et al. [20] referred eleven basioccipitals, five stapedes and one basisphenoid from the Cambridge Greensand Member to the ophthalmosaurine ophthalmosaurid *Acamptonectes* sp. Fischer et al. [20] misspelled the collection number of a basioccipital referred to as *Acamptonectes* sp.: in their paper, specimen CAMSM B56961 is actually CAMSM B57961. Now that additional ophthalmosaurine ophthalmosaurids have been found in Cretaceous strata of Eurasia [99], the referral of these remains to the Hauterivian genus *Acamptonectes* by Fischer et al. [20] is disputable, even if one basioccipital (CAMSM B57962) and one basisphenoid (NHMUK PV R2341) exhibited autapomorphic features of *Acamptonectes*. Accordingly, we refer all these *Acamptonectes* sp. remains (i.e. CAMSM B57955 [basioccipital], CAMSM B57949 [basioccipital], CAMSM B57942 [basioccipital], CAMSM B57952 [basioccipital], CAMSM B56961 [basioccipital], CAMSM TN1735 partim [basioccipital], CAMSM TN1751 partim [basioccipital], CAMSM TN1753 partim [basioccipital], CAMSM TN1755 partim [basioccipital], GLAHM V.1463 [basioccipital, Newmarket road pits], NHMUK 35301 [basioccipital], CAMSM B58074 [stapes], CAMSM B58075 [stapes], CAMSM B58079 [stapes], CAMSM TN1757 partim [stapes], GLAHM V.1535/1 [stapes], NHMUK R2341 [basisphenoid]) to Ophthalmosaurinae indet. The holotype of *I. cantabrigiensis* (NHMUK 43989) lacks distinguishing features from other ophthalmosaurines; accordingly, this taxon is considered here as nomen dubium.

### Description

#### Dentition (morphotype 3; see Systematic Paleontology above for a complete list of specimens; Figure 15 A, B)

The teeth are recurved medially. The crown is conical, textured by light apicobasal ridges, and appears small compared to the apicobasal height of the tooth (19% in NHMUK 47268 partim). The apex is pointed and smooth. Both the acellular cementum ring and the root are smooth (no apicobasal ridges) and their cross-section is rounded. Some teeth have slightly flattened surface of on their roots, but lack the well-defined angles seen in the other tooth morphotypes (‘*Platypterygius*’ sp. and *Sisteronia seeleyi*).

A squared root section is a synapomorphy of platypterygiine ichthyosaurs [20] (but reversed in *Aegirosaurus* [1], [100]). This tooth morphotype does not correspond to *Aegirosaurus* [1], being recurved, having a much smaller crown and a smooth apex. This tooth morphotype is however similar to that of *Ophthalmosaurus icenicus* [73]. Accordingly, we refer the tooth morphotype 3 to Ophthalmosaurinae indet.

#### Humerus (morphotype 4; see Systematic Paleontology above for a complete list of specimens; Figure 15 C)

The humerus is usually small and stout; but larger specimens (such as CAMSM TN1755 partim) have a more slender shape. The short trochanter dorsalis and the deltopectoral crest are well developed, although the latter may be reduced in some specimens. A similar variability has already been reported in the ophthalmosaurine *A. densus* [20]. The humerus forms three distal facets that are sub-equal in size. The posterior-most (ulnar) facet is markedly deflected posterolaterally and has a concave margin in dorsal view. The median (radial) facet is the largest and squared or slightly dorsoventrally elongated. The anterior-most (accessory) facet is often slightly deflected anterolaterally.

This humerus morphotype has been interpreted in various ways since Lydekker [68], [98]. He considered the three distal facets as indicative of *Ophthalmosaurus*, but the equal size of these three facets in one of these humeri, NHMUK 43989, differed from *O. icenicus*, justifying a new species, *Ophthalmosaurus cantabrigiensis*. Then, McGowan & Motani [69] considered this species to belong to *Brachypterygius*, mainly because it did not resemble *O. icenicus* enough and because they already inferred the presence of *Brachypterygius* in the Cambridge Greensand Member using basicranium evidence. Evidence for a referral of this humerus morphotype to *Brachypterygius* is, however, poor. Indeed, the largest facet on this humerus morphotype (to which the holotype of *O. cantabrigiensis* belongs) is the ‘median’ facet, a condition never observed in any ichthyosaur whose intermedium contacts the humerus: in these ichthyosaurs, the intermedium facet is less than half the size of the radial or the ulnar facets (*B. extremus* [73], [101]; pers. obs. on holotype NHMUK R3177; *Aegirosaurus* [72]; *Maiaspondylus* [15]); a similar interpretation for these morphotype 3 humeri would imply an enormous intermedium, larger than both the radius and the ulna, a condition never seen in Ichthyosauria. Moreover, the radial and ulnar facet are both invariably markedly deflected outwards in the above-mentioned taxa (ibid.), whereas only the ulnar facet is consistently deflected outwards (posteroventrally) in the humerus morphotype 3, as in ophthalmosaurine ichthyosaurs [20]. Kear & Zammit [26] recently casted doubt on the validity of this character by studying two in utero specimens that they referred to the platypterygiine taxon ‘*Platypterygius*’ *australis*, which presumably exhibited the same morphology. However, it is clear that the ossification of the humeri that they figure is far from complete ([26]:Figure 2); thus their shape cannot be assessed unambiguously; moreover, adults representatives of this taxon do not exhibit this peculiar morphology [13]. The degree of deflection of the anterior facet forms a wide spectrum in humerus morphotype 3 (ophthalmosaurine), within which only some (usually small) specimens such as NHMUK 43989 (holotype of *O. cantabrigiensis*), CAMSM B58055, and CAMSM TN1727 partim have a slightly anterolaterally deflected anterior facet. This is likely a juvenile condition that disappears with ontogeny, as in ‘*P.*’ *australis* [26]. Moreover, some specimens of adult ophthalmosaurines also show a slightly deflected anterior facet (e.g. GLAHM 132855, holotype of *A. densus*; LEICT G1.2001.016, *Ophthalmosaurus* sp.; GLAHM V1070, *Ophthalmosaurus icenicus* [20]; V.F., pers. obs. on GLAHM, NHMUK, MJML, and CAMSM material). Similarly, the relative size of the anterior facet in ophthalmosaurines also forms a wide spectrum (e.g. [80], [83]; V.F., pers. obs. on GLAHM, NHMUK, MJML, and CAMSM material) within which the holotype of *O. cantabrigiensis* falls satisfactorily. Therefore, we consider the evidence for a referral of this morphotype to *Brachypterygius* as unfounded, and that its morphology falls within the known spectrum for ophthalmosaurines ophthalmosaurids and lacks autapomorphies in the current state of our knowledge. Accordingly, we refer this morphotype to Ophthalmosaurinae indet.

#### Epipodium (IRSNB GS60; Figure 15 D)

IRSNB GS60 is an anterior accessory epipodial element of a forefin. It is elongated proximodistally. This element bears facets for humerus, radius, radiale, and the first autopodial element of the anterior accessory digit. The radial facet is the largest and the humeral and radiale facet are large and equal in size. The humeral and radial facets form a 90° angle. The anterior surface is saddle-shaped rather than convex or flat and its overall shape is not crescent-like. The dorsal half is much thicker than the ventral half.

Accessory epipodial elements are frequent in ophthalmosaurids, but they greatly differ in shape (compare [2], [21], [77], [102]). IRSNB GS60 appears strikingly similar to that of many large specimens of *Ophthalmosaurus icenicus* (V.F., pers. obs. on GLAHM, NHMUK, MJML, and CAMSM material). The lack of a crescentic shape differs from the anterior accessory epipodial element of *Sveltonectes insolitus* and the pisiform of ‘*P.*’ *americanus* [14] and the combination of a proximodistal elongation+a large humeral facet+three additional facets differs from all other platypterygiine ophthalmosaurs for which the epipodium is known [13], [16], [77], [102]. We interpret IRSNB GS60 as an ophthalmosaurinae anterior accessory epipodial element because that morphology has only been found in *O. icenicus* and in the poorly known but probably closely related ‘*Paraophthalmosaurus*’ [103] (V.F. pers. obs. on holotype in SSU) and ‘*Yasykovia*’ [104] so far. Both of these are considered as junior synonyms of *Ophthalmosaurus* by Maisch & Matzke [105] and McGowan & Motani [69].

### Systematic Paleontology

Ophthalmosauridae indet.

1869 *Ichthyosaurus doughtyi* Seeley [50] : xvii

#### Note

The holotype of *I. doughtyi* (CAMSM B58044, from the Cambridge Greensand Member) is a partial humerus, belonging to a juvenile ichthyosaur. The presence of a preaxial accessory facet allows assignment to Ophthalmosauridae, but this specimen lacks diagnostic features. It is therefore referred to Ophthalmosauridae indet. and *Ichthyosaurus doughtyi* is regarded here as a nomen dubium. Several other propodials cannot be assigned more precisely than Ophthalmosauridae indet. These morphotypes are described in Text S6.

Ichthyosauria insertae sedis

*Cetarthrosaurus walkeri* Seeley, 1873 [106] (Seeley, 1869 [50])

Figure 16

**Figure 16:**
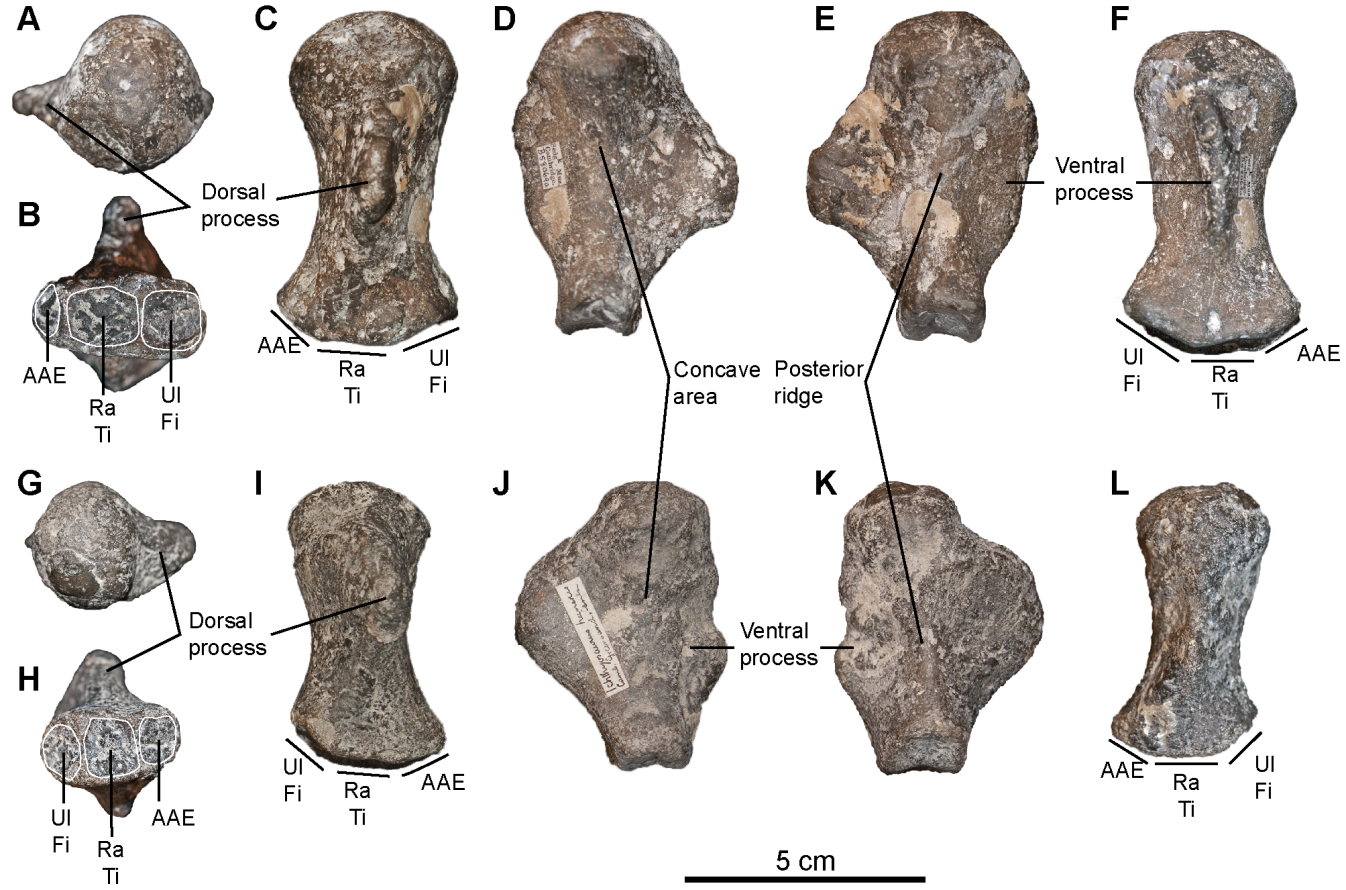
*Cetarthrosaurus walkeri*, propodials. A–F: Holotype (CAMSM B58069), in proximal (A), distal (B), dorsal (C), anterior (D), posterior (E), and ventral (F) views. G–L: referred specimen (CAMSM X50170), in proximal (G), distal (H), dorsal (I), anterior (J), posterior (K), and ventral (L) views. Note the high aspect ratio, the rounded capitulum disconnected from the shaft trochanters, and the high and lamellar dorsal trochanter. Abbreviations: AAE: anterior accessory epipodial element; Fi: fibula; Ra: radius; Ti: tibia; Ul: ulna.

#### Holotype

CAMSM B58069, a propodial from the Cambridge Greensand Member (Lower Chalk Formation), early Cenomanian, but phosphatized and reworked from the top (late Albian) of the Gault Formation.

#### Referred material

CAMSM X50170, from the same age and locality as the holotype.

#### Emended diagnosis

*Cetarthrosaurus walkeri* possesses the following autapomorphies within Ichthyosauria: propodial with hemispherical capitulum disconnected from dorsal and ventral trochanters; elongated and slender shaft (axial length/mid-shaft width ratio = 2.93 in holotype and 3.00 in referred specimen); sheet-like ventral trochanter parallel to the long axis.

Additionally, among Ichthyosauria, the combination of a three-faceted propodial, including a small facet for a preaxial accessory element and a distally-facing ulnar/fibular facet is only shared by: one femur of *Stenopterygius* quadriscissus [86], humerus and femora of some specimens of ‘*Platypterygius*’ sp. from England (this work), humerus and femora of ‘*P.*’ *australis* [13]; humerus of *Caypullisaurus* [107]; an unnamed taxon from Canada [23].

#### Occurrence

Late Albian of the Gault Formation reworked in the Cambridge Greensand Member. No evidence for presence in the upper (early Cenomanian) part of the Cambridge Greensand Member.

#### Note

The holotype of *C. walkeri* (CAMSM B58069) was described by Seeley [50], [106] as a right femur of very unusual shape. Seeley first named *walkeri* as a new species of the genus *Ichthyosaurus* [50]. But his comparison of the propodial with other ichthyosaurs and cetaceans led him to propose a new generic referral for this specimen four years later [106]. Later, this taxon was considered as a mosasaurid (Hulke *in* Lydekker [98]; [3]) and disappeared from the literature. McGowan & Motani's review [69] considered *I. walkeri* as a nomen dubium without discussion and did not mentioned *Cetarthrosaurus*.

During this study, a small right propodial (CAMSM X50170, marked as ‘*Ichthyosaurus* humerus, Cambridge Greensand, Cambridge’) and strikingly similar to CAMSM B589069, was found. It shares all the peculiar features of the holotype of *Cetarthrosaurus walkeri*, but its dorsal surface is less eroded, allowing a better description of that peculiar propodial morphotype. Despite its unusual shape, this propodial is clearly ichthyosaurian (contra Hulke *in* Lydekker [98]). The presence of three distal facets suggests relationship with Ophthalmosauridae, but at least one specimen of the basal baracromian *Stenopterygius* is known to have three distal facets on its femur as well [86]. Moreover, the hemispherical capitulum separated from dorsal and ventral trochanter is unique among post-Triassic ichthyosaurs. Yet, this propodial is diagnostic and, therefore, the taxon *Cetarthrosaurus walkeri* must be considered as a valid, albeit poorly known, late Albian ichthyosaur.

### Description

*Cetarthrosaurus* is only known from two propodials (Figure 16). Their shape is so unusual that is difficult to decipher the limb they belong to. Accordingly, we describe them as propodials and compare them to humeri and femora of neoichthyosaurians. The shaft of the propodial is constricted and elongated (axial length/anterodistal length = 64.52 mm/33.35 mm = 1.93 in the holotype and 61.19 mm/27.51 mm = 2.22 in CAMSM X50170) and the capitulum is hemispherical. Both the anterior and the posterior surfaces of the shaft are saddle-shaped, but the anterior one is flatter (whereas it is markedly flat or concave in ichthyosaurs [69]). The dorsal trochanter is extremely high: its height is more than 80% the height of the capitulum (even the femur having the largest dorsal trochanter of the CAMSM greensand material [CAMSM B58059] has a ratio of 56.7%, because the capitulum of ophthalmosaurids is usually much larger than that of *C. walkeri*). The dorsal trochanter is oblique, only slightly plate-like (i.e. the dorsal surface is not flat-topped but oblique and bordered by concave areas; this condition is therefore ‘intermediate’ between basal thunnosaurians and ophthalmosaurids), and extends up to the distal edge of the propodial through a shallow ridge confluent with the dorsal edge of the anterior accessory facet. The ventral trochanter forms a prominent, long, and sheet-like axial ridge bordered by concave areas. Unusually, these trochanters do not merge with the capitulum, as noted by Seeley [50]. Distally, the propodial has three concave facets: a small anterior accessory facet, and two large squared facets for radius/tibia and ulna/fibula. The radial/tibial facet faces distally and the ulnar/fibular facet faces posterodistally.

### Diversity curves

**Figure 17:**
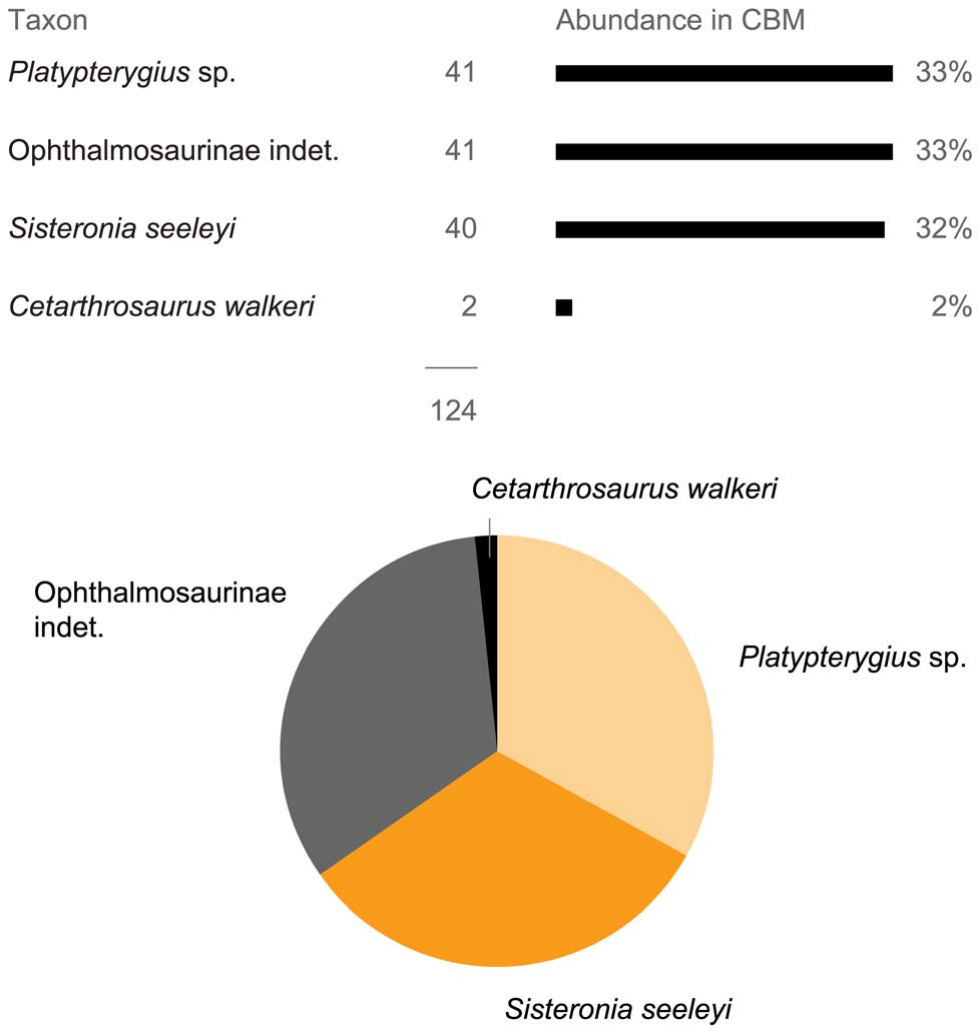
Stage-level taxonomic diversity of Cretaceous ichthyosaurs compared to previous assessments; the number of genera has dramatically increased since year 2002. The position of each stage on the X-axis is proportional to its duration. The grey line represents the generic diversity as of 2002. See Text S7 for the dataset.

The taxonomic diversity of Cretaceous ichthyosaurs is now significantly higher than hypothesized a few years ago (Figure 17). The Berriasian diversity has been increased because of the recognition of Late Jurassic ichthyosaurs in this stage: *Caypullisaurus bonapartei* [108], [109], *Aegirosaurus* sp. (as a Lazarus taxon [1]) and cf. *Ophthalmosaurus* [20]. Despite the description of new fossils from the Valanginian and Hauterivian from western Europe, these stages are still inadequately known, and constitute a ‘diversity low point’ for the Cretaceous. Indeed, the number of specimens known from this interval is extremely low: RGHP LA 1 is the first diagnostic ichthyosaur reported from the Valanginian [1], and only of handful of ichthyosaur specimens are known from the Hauterivian [19], [20], [110].

**Figure 18:**
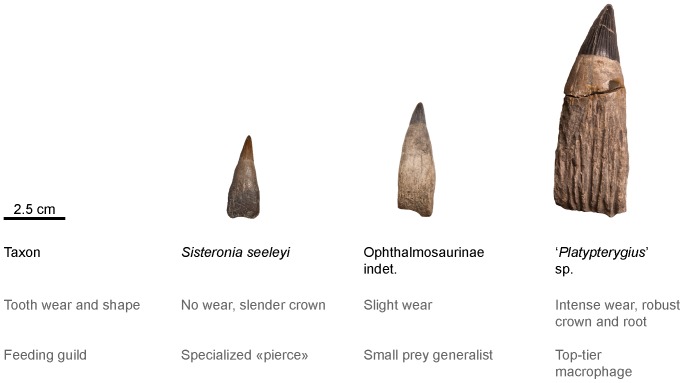
Evolution of ichthyosaur taxonomic richness during the Jurassic and Cretaceous. The Aptian and Albian are split in two and three substages, respectively. The generic curve (black) considers *Platypterygius* as a single taxon. See Text S7 for the dataset.

Diversity explodes during the Barremian, with the recognition of several platypterygiine ophthalmosaurids such as *Sveltonectes insolitus* and *Simbirskiasaurus birjukovi* from western Russia [21], [111], ‘*P.*’ *sachicarum* [112] and ‘*P.*’ *hauthali* [113], [114] from South America, in addition to *Malawania anachronus* from Iraq [19]. The diversity diminishes during the Aptian (Figures 17, 18), probably because of sampling and taxonomic biases, because only a handful of diagnostic Aptian ichthyosaurs have been recovered worldwide [95], [99]. Then, the diversity becomes very high during the Albian. The generic curve remains rather constant because ‘*Platypterygius*’ was considered as a single entity in this curve; if recent advances regarding the polyphyly and status of *Platypterygius* [18], [22], [111] are taken into account, it is even possible that the generic taxonomic richness will equal the specific one and reach a value of 10 during the Albian, as suggested by yet unpublished analyses [71]. Splitting the Aptian and the Albian (Figure 18) does not change the picture, but indicates that a high diversity (eight species) is restricted to the late Albian. Comparable parvipelvian ichthyosaur diversity has only been reported in the early Toarcian Lagerstätten of western Europe, where five genera and as many as eleven species have been reported [69], [105], [115]–[119], and the Tithonian strata of South America, Germany, England, and Russia, containing seven species and four genera [72], [103]–[105], [107], [120]–[128]. The ichthyosaur diversity then severely drops during the Cenomanian and reaches zero by Turonian times.

The taxonomic diversity of Cretaceous ichthyosaurs is now equivalent to or greater than that of their Jurassic ancestors, both at the generic and specific levels, contrary to previous assumptions [4], [129], [130]. Indeed, the diversity frequently reaches four to five genera and seven to eight species whenever fossil-rich sediments occurring in distinct basins are found, such as the Hettangian–Sinemurian [69], [131]–[135] of western Europe, the Tithonian of England (top of the Kimmeridge Clay Formation; [136], [137], Germany [72], [100], [138], [139] and South America [107], [108], [140], and several periods during the Albian [22]. The extremely abundant material from the Toarcian (possibly several thousands of specimens [141]) certainly biases the record. A ‘safer’ interpretation of these fluctuating curves is to consider the diversity of ophthalmosaurids was possibly rather constant from their initial Middle Jurassic radiation onwards and only dropped severely at the beginning of the Late Cretaceous.

## Discussion

### The Albian ichthyosaurs from western Europe

In his Catalogue, Seeley [50] named four new species from the Cambridge Greensand Member: *Ichthyosaurus walkeri*, *Ichthyosaurus doughtyi*, *Ichthyosaurus bonneyi*, and *Ichthyosaurus platymerus*. He did not figure the specimens, nor did he designate holotypes, and only formally described a cast of the holotype specimen of *I. walkeri*. Only *I. walkeri* was subsequently re-described, figured, and made the type species of a new genus, *Cetarthrosaurus* [106], a rare decision at that time. Lydekker [98] and McGowan & Motani [69] considered Seeley's three other species as invalid, being nomina nuda. However, specimens CAMSM B58044, CAMSM B58057, CAMSM B58062 are clearly marked as holotype specimens of *I. doughtyi*, *I. platymerus*, and *I. bonneyi*, respectively; this permits comparison of these specimens with other material and assessment of their validity. Lydekker [98] named *Ophthalmosaurus cantabrigiensis* on the basis of a left humerus (NHMUK 43989) from the Cambridge Greensand Member. McGowan & Motani [69] considered this humerus as indicative of the presence of *Brachypterygius* (*B. cantabrigiensis*) in the Cambridge Greensand Member, because they interpreted the large median facet as a facet for the intermedium. Yet, they also noted the presence of basioccipitals and humeri referable to *Ophthalmosaurus* in the same member.

Our analysis indicates the presence of three common and distinct taxa represented by numerous diagnostic bones (Table 1): ‘*Platypterygius*’ sp., *Sisteronia seeleyi*, Ophthalmosaurinae indet., and an additional but rare taxon: *Cetarthrosaurus walkeri*. Appendicular bones such as humeri and femora, which appear to be more interspecifically variable within ichthyosaurs, even suggest a higher diversity, and probably reflect the specific diversity. *Ichthyosaurus doughtyi* is an indeterminate ophthalmosaurid, *Ichthyosaurus bonneyi* and *Ichthyosaurus platymerus* are not diagnostic and can be referred to as ‘*Platypterygius*’ sp., and *Brachypterygius cantabrigiensis* is an indeterminate ophthalmosaurine ophthalmosaurid, which supports Lydekker's [98] opinion, given the state of knowledge at his time. Therefore, there is no solid evidence for the presence of *Brachypterygius* in the Cretaceous of Europe. The previous stratigraphic range of *Brachypterygius* (Kimmeridgian–Albian) was one of the main reason why Ensom et al. [142] associated a fragmentary skeleton from the Berriasian of England to this genus; our data suggest this referral is not substantiated.

*Platypterygius hercynicus* in coeval deposits from northwestern France [18] should be added to the assemblage described above. Many isolated bones from the Cambridge Greensand Member also closely resemble ‘*P.*’ *hercynicus* but cannot be attributed to this species unambiguously. The humerus morphotype 4 morphotype, exhibiting four distal facets has only been reported in ‘*P.*’ *hercynicus* [74], [76] and two *Platypterygius* sp. specimens from North America distinct from ‘*P.*’ *americanus* [16], [77]. Some basioccipitals (with a raised floor of foramen magnum), teeth, and femora (with three distal facets including one probably for the astragalus) are also identical to that of ‘*P.*’ *hercynicus* (see [74], [76]).

Additionally, the two identified humeral morphotypes here referred to ‘*Platypterygius*’ sp. indicate that another large platypterygiine roamed western Europe; this second taxon may correspond to the poorly known species ‘*P.*’ *campylodon*, but the type material of this taxon does not contain postcranial remains (V.F., pers. obs. on CAMSM material), preventing a thorough comparison. However, it should be noted that recent works have highlighted intrageneric or even intraspecific variability in the formation of the distal facets in humeri [13], [16] or the fact that the ossification of the humerus may be unrelated to the presence of extrazeugopodial elements. The humerus of *Sveltonectes insolitus* possesses two distal facets, but the forefin also possessed a moon-shaped anterior accessory element that contacted the humerus without imprinting it [21]. This suggests that the number of distal facets (especially the absence/presence of minute anterior and posterior accessory facets) may not be a reliable criterion for assessing taxonomic diversity.

Intuitively, femora would also have a taxonomic signal masked by intraspecific variability and by the degree of perichondral ossification. Within known ophthalmosaurids, femora tend to have a wide diversity of forms, even if relatively few femora are known: each taxon possesses its own morphotype, summarized in Table 3. It is therefore impossible in the current state of our knowledge to have an idea of the variability of these features. Yet, femora from the Cambridge Greensand Member still augment this diversity of femoral morphologies of ophthalmosaurids, by having five different morphotypes (Table 4; Text S6). This disparity may therefore indicate that more than four ichthyosaur taxa co-habited the ecosystem of the Cambridge Greensand Member, as suggested by humerus evidence.

**Table 3:** Overview of the morphological disparity in ophthalmosaurid femora.

Taxon	Facets	Including one for	Capitulum shape	Trochanters
‘*P. campylodon*’ (Kiprijanoff material)	2	/	Triangular	High
*O. icenicus*	2	/	Triangular	Small
*Maiaspondylus lindoei*	3	Astragalus	?	?
‘*P.*’ *hercynicus*	3	Astragalus	Oblong	Medium
‘*P.*’ *australis*	3	AAE	Rounded	Medium
‘*P.*’ *americanus*	3	PAE	Triangular	High
*Sveltonectes insolitus*	2	/	Triangular	High

Abbreviations: AAE: anterior accessory epipodial element; PAE: posterior accessory epipodial element. References: ‘*P. campylodon*’: Kiprijanoff [91]; *O. icenicus*: Andrews [80]; *M. lindoei*: Druckenmiller & Maxwell [22]; ‘*P.*’ *hercynicus*: Kolb & Sander [76]; ‘*P.*’ *australis*: Zammit et al. [13]; ‘*P.*’ *americanus*: Maxwell & Kear [14]; *S. insolitus* [21].

**Table 4:** Femoral morphotypes recognized in the Cambridge Greensand member.

Morphotype (# of specimens)	Facets	Including one for:	Capitulum shape	Trochanters
FM1 (8)	3	AAE	Triangular	Medium
FM2 (4)	2	/	Triangular	Medium
FM3 (1)	2	/	Rounded	High
FM4 (1)	3	Astragalus?	Rounded	High
FM5 (*C. walkeri*) (2)	3	AAE	Round, not connected to trochanters	High and lamellar

Abbreviations: AAE: anterior accessory epipodial element; PAE: posterior accessory epipodial element.

**Figure 19:**
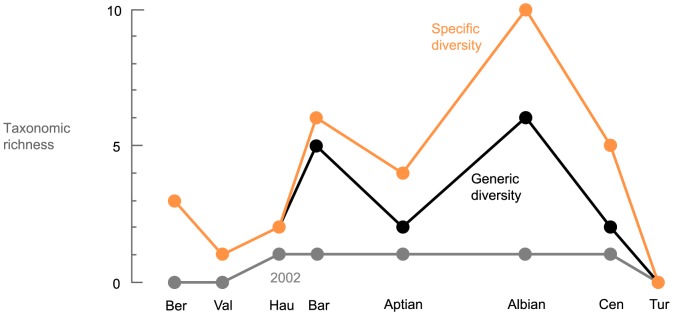
Relative abundance of the taxa recognized in the Cambridge Greensand Member. Platypterygiine taxa are colored in orange, ophthalmosaurine taxa in grey, and insertae sedis in white.

In terms of relative abundances (Figure 19), ‘*Platypterygius*’ sp. represents only 33% (41 specimens) of the assemblage. Ophthalmosaurinae accounts for 33% of the assemblage (41 specimens as well), *Sisteronia seeleyi* represents 32% (40 specimens) of the assemblage, and *C. walkeri* completes the picture with a relative abundance of 2% (2 specimens). These proportions contradict the popular belief of monogeneric (*Platypterygius*) ichthyosaur assemblages in the Cretaceous (e.g. [4], [5]). Moreover, the count of ‘*Platypterygius*’ sp. is probably overestimated relatively to other taxa because this taxon (and possibly *C. walkeri*) is the only one to which one femoral morphotype has been tied, increasing the number of referable specimens, whereas the femora of Cretaceous ophthalmosaurines and *Sisteronia seeleyi* are yet unknown. Similarly, the count for *C. walkeri* may be underestimated, because only two propodials are referable to this poorly known taxon. It is even possible that the femora of *C. walkeri* belong to one of the other ichthyosaur taxa recognized here, but this could only be proven with articulated material. On the subfamilial level, however, Platypterygiinae dominates the assemblage with a relative abundance of 65% versus 33% for Ophthalmosaurinae.

The ichthyosaur assemblage of the Cambridge Greensand Member, containing four co-occurring genera, is the most diversified assemblage ever reported in a single sedimentary body of Cretaceous age. The persistence of ‘*P.*’ *hercynicus* in the latest Albian of northwestern France and possibly Cambridge area and the co-occurrence of large ‘*Platypterygius*’ sp. and *Sisteronia seeleyi* in the Vocontian Basin suggest that western Europe was a diversity hot-spot for Albian–earliest Cenomanian ichthyosaurs, a few million years prior to their final extinction, at the Cenomanian–Turonian boundary [3].

Preliminary assessment of the marine reptile assemblage of a new latest Albian–earliest Cenomanian locality in western Russia (V.F., pers. obs. on SSU material) suggests a similar diversity in this deposit as well, with the presence of at least three ichthyosaur taxa, including platypterygiines and ophthalmosaurines, and with distinct tooth morphologies. Articulated material is needed for a better understanding of these forms, but this suggests a high diversity in ichthyosaurs of the Albian–Cenomanian boundary in western Russia as well. This situation appears similar to the Lower Albian of Canada, where three to four taxa have recently been recognized [15], [16], [22], [23] and markedly contrasts with the monospecific ichthyosaur assemblages in the Albian of Australia [12], [13] and U.S.A. [22], despite the fact that a large number of specimens have been discovered in numerous localities, at least in Australia. Therefore, whereas the taxonomic richness of late Albian ichthyosaurs now reaches eight species (Figure 18); and probably as many genera if *Platypterygius* is split according to recent revisions [18], [111]), this diversity shows a strong geographical variability and was not uniformly high worldwide.

Beta diversity is more difficult to assess, as most Cretaceous ichthyosaur localities have yielded a handful of specimens, at best. Even if the Albian record is generally better than that of the rest of the Cretaceous, the Albian ichthyosaur localities are not contemporaneous. Nevertheless, Albian ichthyosaurs appear to have their biogeographical ranges limited to a regional scale; indeed, not a single species is shared between the Australia, North American, Canadian and European provinces, suggesting a high beta diversity. At a smaller geographic scale, ichthyosaur assemblages appear similar, as suggested by the French and eastern England localities described above. It is nevertheless possible that the apparent endemism between the major Albian ichthyosaur provinces is due to poor sampling. Indeed, late Albian ichthyosaurs of western Europe have similarities with the early Albian Canadian assemblage: ‘*P.*’ *hercynicus* is northwestern France and similar ‘*Platypterygius*’ sp. remains in the Cambridge Greensand Member resemble the large but poorly preserved *Platypterygius* sp. described by Maxwell & Caldwell [16], having a similar humerus and forefin. Furthermore, at least one isolated ilium from the Gault Formation (NHMUK unnumbered) matches that of *Athabascasaurus bitumineus* from the early Albian of Canada [22], [23], being markedly recurved posterodorsally. A better knowledge of the teeth and basicranium of the Canadian taxa could help to assess their presence or absence in Eurasian ecosystems.

### Ichthyosaur-dominated ecosystems in the late Early Cretaceous of Europe

Tooth size and shape varies greatly among ichthyosaur taxa in the Cambridge Greensand Member. ‘*Platypterygius*’ sp. possesses the largest and most robust teeth: the conical crown is robust, and the numerous apicobasal ridges texturing the crown, the acellular cementum ring and the root likely reinforced the resistance of the teeth under dorsoventral stress, as in corrugated materials. *Sisteronia seeleyi* possesses the smallest and most gracile teeth: the crown is pointed and slender, the tooth lacks conspicuous apicobasal ridges basally to the crown, and the root is slender and markedly compressed transversely. Ophthalmosaurinae indet. falls in between these extremes.

**Figure 20:**
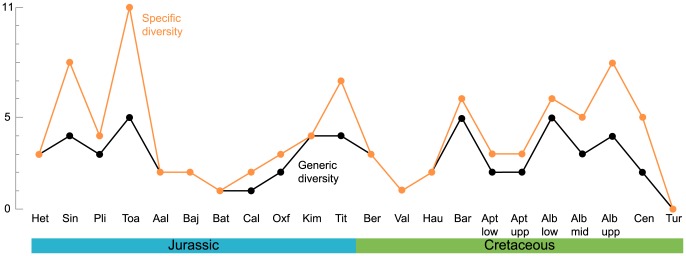
Teeth from the upper part of the Gault Formation and Cambridge Greensand Member (from left to right: CAMSM TN1779 partim; NHMUK R16 partim; NHMUK 47235), illustrating the three feeding guilds colonized by ichthyosaurs in this ecosystem.

Wear patterns are similarly contrasted between ‘*Platypterygius*’ sp. and *Sisteronia seeleyi*. ‘*Platypterygius*’ sp. teeth are by far the most worn: the majority of isolated (possibly shed) teeth fall within the most severe category of wear (apex broken and polished), and articulated specimens show a large proportion of functional teeth belonging to this wear category as well (e.g. CAMSM TN283; RGHP PR 1). By contrast, nearly all *Sisteronia seeleyi* teeth are only slightly polished or still have pristine enamel texture on the apex. Articulated rostra of *Sisteronia seeleyi* are currently not available, preventing statistical wear analysis on functional teeth. Similarly, very few ophthalmosaurine teeth occur in the Cambridge Greensand Member, preventing any evaluation of their wear with confidence. Yet, preserved ophthalmosaurine tooth apices belonging to all categories of wear are found within this small assemblage. This situation, where the most robust teeth are also the most worn and vice-versa, suggests contrasted diet for these taxa. These shape and wear differences also match size differences (Figure 20).

Accordingly, we propose that these ichthyosaurs colonized distinct feeding guilds: ‘*Platypterygius*’ sp. probably belongs to a guild of top-tier predators, possibly feeding on tetrapods (among other prey), given the robust tooth shape and the intense tooth wear with frequent apical tooth breakage and enamel spalling, as already suggested for ‘*P.*’ *australis* [10]. These features are regarded as indicative of such diet in marine crocodyliforms too [143]. Ophthalmosaurines are considered as opportunistic generalists, because their tooth shape and wear closely resembles those in *Aegirosaurus* and adult *O. icenicus*, considered generalists [1], [99]. The delicate, slender and unworn teeth of *Sisteronia seeleyi* suggest that it belongs to a ‘pierce’-oriented guild, feeding on soft and small prey such as small fishes and neocoleoid cephalopods, according to Massare's criteria [25], [28] (Figure 20). These ichthyosaurs therefore occupied up to three feedings guilds within the single ecosystem of the upper Gault Formation/Cambridge Greensand Member, despite the presence of a diversified plesiosaur assemblage including the gigantic pliosaur *Polyptychodon interruptus* [50], [68], [144]. The presence of ichthyosaurs at several levels of the trophic chain of one ecosystem has not been previously reported from assemblages dating to after the Early Jurassic, when ichthyosaurs dominated the ecosystems of the European archipelago [24], [25], [119], [145] together with several plesiosaur taxa, including large rhomaleosaurids [146]–[151]. The fact that ichthyosaurs from the late Albian–early Cenomanian deposits of Europe and possibly Russia, like their Early Jurassic ancestors, colonized multiple ecological niches despite the presence of numerous other marine reptile taxa shows that the ‘last’ ichthyosaurs were still a diversified and important component of the marine ecosystems up to a few millions years prior to their extinction, at least in Europe and Russia.

## Conclusions

The thorough analysis of the diversity of the rich ichthyosaur assemblages of middle Albian–earliest Cenomanian of England and southern France yields the following results:

1. We recognize four taxa as valid: ‘*Platypterygius*’ sp., *Sisteronia seeleyi* gen. et sp. nov., Ophthalmosaurinae indet., and *Cetarthrosaurus walkeri*. We consider *Ichthyosaurus doughtyi*, *Ichthyosaurus bonneyi*, *Ichthyosaurus platymerus*, *Ichthyosaurus angustidens* and *Brachypterygius cantabrigiensis* as invalid; there is no solid evidence for the presence of *Brachypterygius* in the Cretaceous.
2. Ichthyosaurs occupied several feeding guilds within the mid-Cretaceous ecosystems of western Europe: ‘*Platypterygius*’ sp. likely occupied apex predator along with the large pliosauroid *Polyptychodon interruptus*, *Sisteronia seeleyi* occupied a ‘pierce’-oriented guild, and ophthalmosaurine ophthalmosaurids probably occupied a ‘generalist/opportunist’ guild.
3. These high taxonomic richnesses and strong ecological presences occur a few million years prior to the final extinction of ichthyosaurs. This indicates that the ‘last’ ichthyosaurs were diversified and were still a major component of marine ecosystems, contradicting previous views of ichthyosaur evolutionary history. The alpha diversity of ichthyosaur is, however, highly variable between provinces. This new data provides a whole new context to analyze the extinction of ichthyosaurs.

## Supporting Information

Text S1 **Gault Formation specimens studied here and their assignation, 19 specimens.**
(DOC)

Text S2 **Upper Greensand Formation specimens studied here and their assignation.**
(DOC)

Text S3 **Cambridge Greensand Member specimens studied here and their assignation.**
(DOC)

Text S4 **Marnes Bleues Formation specimens studied here and their assignation.**
(DOC)

Text S5 **Determination key for isolated elements from the Cambridge Greensand Member.**
(DOC)

Text S6 **Description of indeterminate femoral morphotypes.**
(DOC)

Text S7 **Taxa recognized as valid for each stage of the Hettangian–Turonian interval.**
(DOC)

## References

[1] Fischer V, Clément A, Guiomar M, Godefroit P (2011) The first definite record of a Valanginian ichthyosaur and its implication for the evolution of post-Liassic Ichthyosauria. Cretaceous Research 32: 155–163.

[2] McGowan C (1972) The systematics of Cretaceous ichthyosaurs with particuliar reference to the material from North America. Contributions to Geology 11: 9–29.

[3] Bardet N (1992) Stratigraphic evidence for the extinction of the ichthyosaurs. Terra Nova 4: 649–656.

[4] Sander PM (2000) Ichthyosauria: their diversity, distribution, and phylogeny. Paläontologische Zeitschrift 74: 1–35.

[5] Lingham-Soliar T (2003) Extinction of ichthyosaurs: a catastrophic or evolutionary paradigm? Neues Jahrbuch für Geologie und Paläontologie, Abhandlungen 228: 421–452.

[6] Wade M (1984) *Platypterygius australis*, an Australian Cretaceous ichthyosaur. Lethaia 17: 99–113.

[7] Wade M (1990) A review of the Australian Cretaceous longipinnate ichthyosaur *Platypterygius* (Ichthyosauria, Ichthyopterygia). Memoirs of the Queensland Museum 28: 115–137.

[8] Kear BP (2002) Dental caries in an Early Cretaceous ichthyosaur. Alcheringa 25: 387–390.

[9] Kear BP (2003) Cretaceous marine reptiles of Australia: a review of taxonomy and distribution. Cretaceous Research 24: 277–303.

[10] Kear BP, Boles WE, Smith ET (2003) Unusual gut contents in a Cretaceous ichthyosaur. Proceedings of the Royal Society of London B Biological Sciences 270: S206–S208. 10.1098/rsbl.2003.0050 PMC1809966 14667384

[11] Kear BP (2005) Cranial morphology of *Platypterygius longmani* Wade, 1990 (Reptilia: Ichthyosauria) from the Lower Cretaceous of Australia. Zoological Journal of the Linnean Society 145: 583–622.

[12] Zammit M (2010) A review of Australasian ichthyosaurs. Alcheringa 34: 281–292.

[13] Zammit M, Norris RM, Kear BP (2010) The Australian Cretaceous ichthyosaur *Platypterygius australis*: a description and review of postcranial remains. Journal of Vertebrate Paleontology 30: 1726–1735.

[14] Maxwell EE, Kear BP (2010) Postcranial anatomy of *Platypterygius americanus* (Reptilia: Ichthyosauria) from the Cretaceous of Wyoming. Journal of Vertebrate Paleontology 30: 1059–1068.

[15] Maxwell EE, Caldwell MW (2006) A new genus of ichthyosaur from the Lower Cretaceous of Western Canada. Palaeontology 49: 1043–1052.

[16] Maxwell EE, Caldwell MW (2006) Evidence for a second species of the ichthyosaur *Platypterygius* in North America: a new record from the Loon River Formation (Lower Cretaceous) of northwestern Canada. Canadian Journal of Earth Sciences 43: 1291–1295.

[17] Zammit M (2012) Cretaceous ichthyosaurs: dwindling diversity, or the Empire strikes back? Geosciences 2: 11–24.

[18] Fischer V (2012) New data on the ichthyosaur *Platypterygius hercynicus* and its implications for the validity of the genus. Acta Palaeontologica Polonica 57: 123–134.

[19] Fischer V, Appleby RM, Naish D, Liston J, Riding JB, et al (2013) A basal thunnosaurian from Iraq reveals disparate phylogenetic origins for Cretaceous ichthyosaurs. Biology Letters 9: 20130021. 23676653 10.1098/rsbl.2013.0021 PMC3730615

[20] Fischer V, Maisch MW, Naish D, Liston J, Kosma R, et al (2012) New ophthalmosaurids from the Early Cretaceous of Europe demonstrate extensive ichthyosaur survival across the Jurassic–Cretaceous boundary. PLoS ONE 7: e29234. 22235274 10.1371/journal.pone.0029234 PMC3250416

[21] Fischer V, Masure E, Arkhangelsky MS, Godefroit P (2011) A new Barremian (Early Cretaceous) ichthyosaur from western Russia. Journal of Vertebrate Paleontology 31: 1010–1025.

[22] Druckenmiller PS, Maxwell EE (2010) A new Lower Cretaceous (lower Albian) ichthyosaur genus from the Clearwater Formation, Alberta, Canada. Canadian Journal of Earth Sciences 47: 1037–1053.

[23] Maxwell EE, Druckenmiller PS (2011) A small ichthyosaur from the Clearwater Formation (Alberta, Canada) and a discussion of the taxonomic utility of the pectoral girdle. Paläontologische Zeitschrift 85: 457–463.

[24] Godefroit P (1996) Biodiversité des reptiles marins du Jurassique inférieur belgo-luxembourgeois. Bulletin de la Société belge de Géologie 104: 67–76.

[25] Massare JA (1987) Tooth morphology and prey preference of Mesozoic marine reptiles. Journal of Vertebrate Paleontology 7: 121–137.

[26] Kear BP, Zammit M (2013) *In utero* foetal remains of the Cretaceous ichthyosaurian *Platypterygius*: ontogenetic implications for character state efficacy. Geological Magazine In Press.

[27] Zammit M (2011) The Australian Cretaceous ichthyosaur *Platypterygius australis*: understanding its taxonomy, morphology, and palaeobiology: University of Adelaide, South Australia. 106 p.

[28] Massare JA (1997) Faunas, behavior, and evolution. In: Callaway JM, Nicholls EL, editors. Ancient Marine Reptiles. San Diego: Academic Press. pp. 401–421.

[29] Schubert BW, Ungar PS (2005) Wear facets and enamel spalling in tyrannosaurid dinosaurs. Acta Palaeontologica Polonica 50: 93–99.

[30] Thewissen JGM, Sensor JD, Clementz MT, Bajpari S (2011) Evolution of dental wear and diet during the origin of whales. Palaeobiology 37: 655–669.

[31] Ross MR (2009) Charting the Late Cretaceous seas: mosasaurs richness and morphological diversification. Journal of Vertebrate Paleontology 292: 409–416.

[32] Owen HG (2012) The Gault Group (Early Cretaceous, Albian), in East Kent, S.E. England; its lithology and ammonite biozonation. Proceedings of the Geologists' Association 123: 742–765.

[33] Juignet P (1974) La transgression crétacée sur la bordure orientale du Massif armoricain. Aptien, Albien, Cénomanien de Normandie et du Maine. Le stratotype du Cénomanien: Université de Caen. 806 p.

[34] Travassac F (2004) Stratigraphie, sédimentologie et géochimie d'une série d'âge barrémien supérieur à albien pro parte du bassin vocontien (SE France) : implications paléoenvironnmentales. Marseille: Ecole doctorale Sciences de l'Environnement d'Aix-Marseille. 54 p.

[35] Scott RW (2009) Uppermost Albian biostrigraphy and chronostratigraphy. Notebooks on Geology 2009/03: 1–16.

[36] Amédro F (2008) Support for a Vraconnian Stage between the Albien sensu stricto and the Cenomanien (Cretacous System). Notebooks on Geology Memoir 2008/02: 83.

[37] Amédro F, Robaszynski F (2008) Zonation by ammonites and foraminifers of the Vraconnian-Turonian interval: A comparison of the Boreal and Tethyan domains (NW Europe/Central Tunisia). Notebooks on Geology, Letter 2008/02: 5.

[38] Lehmann J, Heldt M, Bachmann M, Hedi Negra ME (2009) Aptian (Lower Cretaceous) biostratigraphy and cephalopods from north central Tunisia. Cretaceous Research 30: 895–910.

[39] Kuhnt W, Moullade M (2007) The Gargasian (Middle Aptian) of La Marcouline section at Cassis-La Bédoule (SE France): Stable isotope record and orbital cyclicity. Notebooks on Geology 2007/02: 1–9.

[40] Ogg J, Ogg G, Gradstein FM (2008) A concise geologic timescale. Cambridge.

[41] Gradstein FM, Ogg JG, Schmitz M, Ogg G (2012) The Geologic Time Scale 2012. Oxford, Great Britain: Elsevier Science & Technology. 1176 p.

[42] Benson RBJ, Butler RJ (2011) Uncovering the diversification history of marine tetrapods: ecology influences the effect of geological sampling biases. In: McGowan AJ, Smith AB, editors. Comparing the geological and fossil records: implications for biodiversity studies. London: Geological Society, Special Publications. pp. 191–208.

[43] Butler RJ, Barrett PM, Nowbath S, Upchurch P (2009) Estimating the effects of sampling biases on pterosaur diversity patterns: implications for hypotheses of bird/pterosaur competitive replacement. Palaeobiology 35: 432–446.

[44] Butler RJ, Brusatte SL, Andres B, Benson RB (2011) How do geological sampling biases affect studies of morphological evolution in deep time? A case study of pterosaur (Reptilia: Archosauria) disparity. Evolution 66: 147–162. 22220871 10.1111/j.1558-5646.2011.01415.x

[45] Mannion PD, Upchurch P (2011) A re-evaluation of the ‘mid-Cretaceous sauropod hiatus’ and the impact of uneven sampling of the fossil record on patterns of regional dinosaur extinction. Palaeogeography Palaeoclimatology Palaeoecology 299: 529–540.

[46] Mannion PD, Upchurch P, Carrano MT, Barrett PM (2011) Testing the effect of the rock record on diversity: a multidisciplinary approach to elucidating the generic richness of sauropodomorph dinosaurs through time. Biological Reviews 86: 157–181. 20412186 10.1111/j.1469-185X.2010.00139.x

[47] Hopson PM, Wilkinson IP, Wood MA (2008) A stratigraphical framework for the Lower Cretaceous of England. British Geological Survey Research Reports RR/08/03: 1–87.

[48] Sauvage HE (1882) Recherches sur les reptiles trouvées dans le Gault de l'Est du bassin de Paris. Mémoires de la Société géologique de France, 3e série 2: 21–24.

[49] Breton G (2011) Deux nouvelles espèces de crustacés décapodes de l'Albien du Bassin de Paris. Geodiversitas 33: 279–284.

[50] Seeley HG (1869) Index of the fossil remains of Aves, Ornithosauria and Reptilia, from the Secondary System of Strata Aranged in the Woodward Museum of the Univeristy of Cambridge; Deighton BC, editor. Cambridge.

[51] Martill DM, Unwin DM (2012) The world's largest toothed pterosaur, NHMUK R481, an incomplete rostrum of *Coloborhynchus capito* (Seeley, 1870) from the Cambridge Greensand of England. Cretaceous Research 34: 1–9.

[52] Hopson PM (2005) A stratigraphical framework for the Upper Cretaceous Chalk of England and Scotland with statements on the Chalk of Northern Ireland and the UK Offshore Sector. British Geological Survey Research Reports RR/05/01: 1–102.

[53] Cookson IC, Hughes NF (1964) Microplankton from the Cambridge Greensand (mid-Cretaceous). Palaeontology 7: 37–59.

[54] Barrett PM, Evans SE (2002) A reassessment of the Early Cretaceous reptile ‘*Patricosaurus merocratus*’ Seeley from the Cambridge Greensand, Cambridgeshire, UK. Cretaceous Research 23: 231–240.

[55] Seeley HG (1876) On an associated series of cervical and dorsal vertebræ of *Polyptychodon*, from the Cambridge Upper Greensand, in the Woodwardian Museum of the University of Cambridge. Quarterly Journal of the Geological Society 32: 433–436.

[56] Unwin DM (2001) An overview of the pterosaur assemblage from the Cambridge Greensand (Cretaceous) of Eastern England. Mitteilungen aus dem Museum für Naturkunde in Berlin, Geowissenschaftliche Reihe 4: 189–221.

[57] Robaszynski F, Amedro F, Gonzalez-Donoso JM, Linares D (2008) The Albian (Vraconnian)-Cenomanian boundary at the western Tethyan margins (central Tunisia and southeastern France). Bulletin de la Société géologique de France 179: 245–266.

[58] Woods MA, Wilkinson GK, Booth KA, Farrant AR, Hopson PM, et al (2008) A reappraisal of the stratigraphy and depositional development of the Upper Greensand (Late Albian) of the Devizes district, southern England. Proceedings of the Geologists' Association 119: 229–244.

[59] Bréhéret J-G (1997) L'Aptien et l'Albien de la Fosse vocontienne (des bordures au bassin) : Evolution de la sédimentation et enseignements sur les événements anoxiques. Société géologique du Nord 25: 614.

[60] Wilpshaar M, Leereveld H, Visscher H (1997) Early Cretaceous sedimentary and tectonic development of the Dauphinois Basin (SE France). Cretaceous Research 18: 457–468.

[61] Kennedy WJ, Gale AS, Bown PR, Caron M, Davey RJ, et al (2000) Integrated stratigraphy across the Aptian-Albian boundary in the Marnes Bleues, at the Col de Pré-Guittard, Arnayon (Drôme), and at Tartonne (Alpes-de-Haute-Provence), France: a candidate Global Boundary Stratotype Section and Boundary Point for the base of the Albian Stage. Cretaceous Research 21: 591–720.

[62] Accarie H, Beaudoin B, Dejax J, Friès G, Michard J-G, et al (1995) Découverte d'un dinosaure théropode nouveau (*Genusaurus sisteronis* n. g., n. sp.) dans l'Albien marin de Sisteron (Alpes de Haute-Provence, France) et extension au Crétacé inférieur de la lignée cératosaurienne. Comptes Rendus de l'Académie des Sciences de Paris 320: 327–334.

[63] Herrle JO, Mutterlose J (2003) Calcareous nannofossils from the Aptian-Lower Albian of southeast France: palaeoecological and biostratigraphic implications. Cretaceous Research 24: 1–22.

[64] Haccard D, Beaudoin B, Gigot P, Jorda M (1989) La Javie. In: BRGM, editor. Carte Géologique de France à 1/50 000.

[65] Blainville HMDde (1835) Description de quelques espèces de reptiles de la Californie, précédée de l'analyse d'un système général d'érpetologie et d'amphibiologie. Nouvelles annales du Muséum d'Histoire naturelle, Paris 4: 233–296.

[66] Baur G (1887) On the morphology and origin of the Ichthyopterygia. American Naturalist 21: 837–840.

[67] Arkhangelsky MS (2001) The historical sequence of Jurassic and Cretaceous ichthyosaurs. Paleontological Journal 35: 521–524.

[68] Lydekker R (1889) Catalogue of the fossil Reptilia and Amphibia in British Museum (Natural History). Part II. containing the orders Ichthyopterygia and Sauropterygia. London: Printed by Orders of the Trustees of the British Museum, London.

[69] McGowan C, Motani R (2003) Part 8. Ichthyopterygia; Sues H-D, editor. München: Verlag Dr. Friedrich Pfeil. 175 p.

[70] Johnson R (1977) Size independent criteria for estimating relative age and the relationship among growth parameters in a group of fossil reptiles (Reptilia: Ichthyosauria). Canadian Journal of Earth Sciences 14: 1916–1924.

[71] Fischer V (2013) Origin, biodiversity and extinction of Cretaceous ichthyosaurs. Liège, Belgium: Université de Liège. 576 p.

[72] Bardet N, Fernández M (2000) A new ichthyosaur from the Upper Jurassic lithographic limestones of Bavaria. Journal of Paleontology 74: 503–511.

[73] Kirton AM (1983) A review of British Upper Jurassic ichthyosaurs. Newcastle upon Tyne: University of Newcastle upon Tyne. 239 p.

[74] Kuhn O (1946) Ein skelett von *Ichthyosaurus hercynicus* n. sp. aus dem Aptien von Gitter. Berichte der Naturforschenden Gesellschaft Bamberg 29: 69–82.

[75] Appleby RM (1961) On the cranial morphology of ichthyosaurs. Proceedings of the Zoological Society of London 137: 333–370.

[76] Kolb C, Sander PM (2009) Redescription of the ichthyosaur *Platypterygius hercynicus* (Kuhn 1946) from the Lower Cretaceous of Salzgitter (Lower Saxony, Germany). Palaeontographica Abteilung A (Paläozoologie, Stratigraphie) 288: 151–192.

[77] Adams TL, Fiorillo A (2011) *Platypterygius* Huene, 1922 (Ichthyosauria, Ophthalmosauridae) from the Late Cretaceous of Texas, USA. Palaeontologia Electronica 14: 19A.

[78] Gasparini Z (1988) *Ophthalmosaurus monocharactus* Appleby (Reptilia, Ichthyopterygia), en las calizas litograpficas tithonianas del area Los Catutos, Nequén, Argentina. Ameghiniana 25: 3–16.

[79] McGowan C (1973) The cranial morphology of the Lower Liassic latipinnate ichthyosaurs of England. Bulletin of the British Museum (Natural History) Geology 24: 1–109.

[80] Andrews CW (1910) A descriptive catalogue of the Marine Reptiles of the Oxford Clay, part I. London: British Museum of Natural History. 205 p.

[81] Maxwell EE (2010) Generic reassignment of an ichthyosaur from the Queen Elizabeth Islands, Northwest Territories, Canada. Journal of Vertebrate Paleontology 30: 403–415.

[82] Maxwell EE, Caldwell MW, Lamoureux DO (2011) Tooth histology in the Cretaceous ichthyosaur *Platypterygius australis*, and its significance for the conservation and divergence of mineralized tooth tissues in amniotes. Journal of Morphology 272: 129–135. 21210486 10.1002/jmor.10898

[83] Appleby RM (1956) The osteology and taxonomy of the fossil reptile *Ophthalmosaurus* . Proceedings of the Zoological Society of London 126: 403–447.

[84] Nace RL (1939) A new ichthyosaur from the Upper Cretaceous Mowry Formation of Wyoming. American Journal of Science 237: 673–686.

[85] Araújo R, Smith AS, Liston J (2008) The Alfred Leeds fossil vertebrate Collection of the National Museum of Ireland–Natural History. Irish Journal of Earth Sciences 26: 17–32.

[86] Huene Fv (1922) Die Ichthyosaurier des Lias und ihre Zusammenhänge. Berlin: Verlag von Gebrüder Borntraeger. 114 p.

[87] Delair JB (1960) The Mesozoic reptiles of Dorset. Proceedings of the Dorset Natural History and Arhcaeological Society 81: 59–85.

[88] Carter J (1846) On the occurence of a new species of *Ichthyosaurus* in the Chalk. London Geological Journal 1.

[89] Carter J (1846) Notice of the jaws of an *Ichthyosaurus* from the chalk in the neighbourhood of Cambridge. Reports of the British Association for the Advancement of Science 1845: 60.

[90] Bardet N (1989) Un crâne d'Ichthyopterygia dans le Cénomanien du Boulonnais. Mémoires de la Société académique du Boulonnais 6: 31.

[91] Kiprijanoff W (1881) Studien über die fossilen Reptilien Russlands. Theil 1, Gattung *Ichthyosaurus* König aus dem severischen Sandstein oder Osteolith der Kreide-Gruppe. Mémoires de l'Académie impériale des Sciences de St-Pétersbourg, VIIe série 28: 1–103.

[92] Bardet N (1990) Dental cross-section in Cretaceous Ichthyopterygia: systematic implications. Geobios 23: 169–172.

[93] Sirotti A, Papazzoni C (2002) On the Cretaceous ichthyosaur remains from the Northern Apennines (Italy). Bollettino della Societa Paleontologica Italiana 41: 237–248.

[94] Blain H-A, Pennetier G, Pennetier E (2003) Présence du genre *Platypterygius* (Ichthyosauria, Reptilia) dans le Cénomanien inférieur de Villers-sur-Mer (Normandie, France. Echos des falaises 7: 35–50.

[95] Broili F (1907) Ein neuer *Ichthyosaurus* aus der norddeutschen Kreide. Palaeontographica 54: 139–162.

[96] Nace RL (1941) A new ichthyosaur from the Late Cretaceous of northeastern Wyoming. American Journal of Science 239: 908–914.

[97] Fernández MS, Maxwell EE (2012) The genus *Arthropterygius* Maxwell (Ichthyosauria: Ophthalmosauridae) in the Late Jurassic of the Neuquén Basin, Argentina. Geobios 45: 535–540.

[98] Lydekker R (1888) Note on the classification of the Ichthyopterygia with a notice of two new species. Geological Magazine third series 5: 309–314.

[99] Fischer V, Arkhangelsky MS, Uspensky GN, Stenshin IM, Godefroit P (In Press) A new Lower Cretaceous ichthyosaur from Russia reveals skull shape conservatism within Ophthalmosaurinae. Geological Magazine In Press.

[100] Scheyer TM, Moser M (2011) Survival of the thinnest: rediscovery of Bauer's (1898) ichthyosaur tooth sections from Upper Jurassic lithographic limestone quarries, south Germany. Swiss Journal of Geoscience 104: S147–S157.

[101] McGowan C (1997) The taxonomic status of the Late Jurassic ichthyosaur *Grendelius mordax*: a preliminary report. Journal of Vertebrate Paleontology 17: 428–430.

[102] Pardo-Perez J, Frey E, Stinnesbeck W, Fernández M, Rivas L, et al (2012) An ichthyosaurian forefin from the Lower Cretaceous Zapata Formation of southern Chile: implications for morphological variability within *Platypterygius* . Palaeobiodiversity and Palaeoenvironment 92: 287–294.

[103] Arkhangelsky MS (1997) On a new genus of ichthyosaurs from the Lower Volgian substage of the Saratov, Volga Region. Paleontological Journal 31: 87–90.

[104] Efimov VM (1999) Ichthyosaurs of a new genus *Yasykovia* from the Upper Jurassic strata of European Russia. Paleontological Journal 33: 92–100.

[105] Maisch MW, Matzke AT (2000) The Ichthyosauria. Stuttgarter Beiträge zur Naturkunde Serie B (Geologie und Paläontologie) 298: 1–159.

[106] Seeley HG (1873) On *Cetarthrosaurus walkeri* (Seeley), an ichthyosaurian from the Cambridge Upper Greensand. Quarterly Journal of the Geological Society 29: 505–507.

[107] Fernández M (1997) A new ichthyosaur from the Tithonian (Late Jurassic) of the Neuquén Basin (Argentina). Journal of Paleontology 71: 479–484.

[108] Fernández M (2007) Redescription and phylogenetic position of *Caypullisaurus* (Ichthyosauria: Ophthalmosauridae). Journal of Paleontology 81: 368–375.

[109] Fernández M (2007) Chapter 11. Ichthyosauria. In: Gasparini Z, Salgado L, Coria RA, editors. Patagonian Mesozoic Reptiles. Bloomington and Indianapolis, Indiana: Indiana University Press. pp. 271–291.

[110] Efimov VM (1997) A new genus of ichthyosaurs from the Late Cretaceous of the Ulyanovsk Volga region. Paleontological Journal 31: 422–426.

[111] Fischer V, Arkhangelsky MS, Stenshin IM, Uspensky GN, Godefroit P (In Review) The Russian Cretaceous ichthyosaurs *Simbirskiasaurus birjukovi* and *Pervushovisaurus bannovkensis* . Journal of Vertebrate Paleontology

[112] Paramo ME (1997) *Platypterygius sachicarum* (Reptilia, Ichthyosauria) nueva especie del Cretácio de Colombia. Revista Ingeominas 6: 1–12.

[113] Fernández M, Aguirre-Urreta MB (2005) Revision of *Platypterygius hauthali* von Huene, 1927 (Ichthyosauria, Ophthalmosauridae) from the Early Cretaceous of Patagonia, Argentina. Journal of Vertebrate Paleontology 25: 583–587.

[114] Huene Fv (1927) Beitrag zur Kenntnis mariner mesozoicher Wirbeltiere in Argentinien. Centralblatt für Mineralogie, Geologie und Paläntologie, B 1927: 22–29.

[115] Maisch MW (2008) Revision der Gattung *Stenopterygius* Jaekel, 1904 emend. von Huene, 1922 (Reptilia: Ichthyosauria) aus dem unteren Jura Westeuropas. Palaeodiversity 1: 227–271.

[116] Maisch MW (2001) Neue Exemplare der seltenen Ichthyosauriergattung *Suevoleviathan* Maisch 1998 aus dem Unteren Jura von Südwestdeutschland. Geologica et Palaeontologica 35: 145–160.

[117] Maisch MW (1998) A new ichthyosaur genus from the Posidonia Shale (Lower Toarcian, Jurassic) of Holzmaden, SW-Germany with comments on the phylogeny of post-Triassic ichthyosaurs. Neues Jahrbuch für Geologie und Paläontologie, Abhandlungen 209: 47–78.

[118] Maxwell EE (2012) New metrics to differentiate species of *Stenopterygius* (Reptilia: Ichthyosauria) from the Lower Jurassic of southwestern Germany. Journal of Paleontology 86: 105–115.

[119] Martin JE, Fischer V, Vincent P, Suan G (2012) A longirostrine *Temnodontosaurus* (Ichthyosauria) with comments on Early Jurassic ichthyosaur niche partitioning and disparity. Palaeontology 55: 995–1005.

[120] Gasparini Z, De La Fuente M, Fernández M (1995) Sea reptiles from the lithographic limestones of the Neuquén Basin, Argentina. II international symposium on lithographic limestones. Lleida - Cuenca (Spain): Ediciones de la Universidad Autónoma de Madrid. pp. 81–84.

[121] Spalletti L, Gasparini Z, Veiga G, Schwarz E, Fernández M, et al (1999) Facies anóxicas, procesos deposicionales y herpetofauna de la rampa marina titoniano-berriasiana en la Cuenca Neuquina (Yesera del Tromen), Neuquén, Argentina. Revista Geológica de Chile 1: 109–123.

[122] Buchy MC, Lopez Oliva JG (2009) Occurrence of a second ichthyosaur genus (Reptilia; Ichthyosauria) in the Late Jurassic Gulf of Mexico. Boletin de la Sociedad Geologica Mexicana 61: 233–238.

[123] Buchy M-C (2010) First record of *Ophthalmosaurus* (Reptilia: Ichthyosauria) from the Tithonian (Upper Jurassic) of Mexico. Journal of Paleontology 84: 149–155.

[124] Arkhangelsky MS (1998) On the ichthyosaurian fossil from the Volgian stage of the Saratov Region. Paleontological Journal 32: 192–196.

[125] Arkhangelsky MS (2000) On the ichthyosaur *Otschevia* from the Volgian Stage of the Volga Region. Paleontological Journal 34: 549–552.

[126] Arkhangelsky MS (2001) On a new ichthyosaur of the genus *Otschevia* from the Volgian Stage of the Volga Region near Ulyanovsk. Paleontological Journal 35: 629–634.

[127] Efimov VM (1998) An ichthyosaur, *Otschevia pseudoscythica* gen. et sp. nov. from the Upper Jurassic strata of the Ulyanovsk Region (Volga Region). Paleontological Journal 32: 82–86.

[128] Efimov VM (1999) A new family of ichthyosaurs, the Undorosauridae fam. nov. from the Volgian stage of the European part of Russia. Paleontological Journal 33: 174–181.

[129] Bardet N (1994) Extinction events among Mesozoic marine reptiles. Historical Biology 7: 313–324.

[130] Bardet N (1995) Evolution et extinction des reptiles marins au cours du Mésozoïque. Palaeovertebrata 24: 177–283.

[131] McGowan C (1974) A revision of the longipinnate ichthyosaurs of the Lower Jurassic of England, with description of the new species (Reptilia, Ichthyosauria). Life Science Contributions, Royal Ontario Museum 97: 1–37.

[132] McGowan C (1974) A revision of the latipinnate ichthyosaurs of the Lower Jurassic of England (Reptilia, Ichthyosauria). Life Science Contributions, Royal Ontario Museum 100: 1–30.

[133] McGowan C (1989) *Leptopterygius tenuirostris* and other long-snouted ichthyosaurs from the English Lower Lias. Palaeontology 32: 409–427.

[134] McGowan C (1996) Giant ichthyosaurs of the Early Jurassic. Canadian Journal of Earth Sciences 33: 1011–1021.

[135] Godefroit P (1993) Les grands ichthyosaures sinémuriens d'Arlon. Bulletin de l'Institut Royal des Sciences Naturelles de Belgique Sciences de la Terre 63: 25–71.

[136] Delair JB (1972) Some recent discoveries of Kimmeridgian reptiles at Swindon. Wiltshire Archaeological and Natural History Magazine 67: 12–15.

[137] Taylor MA, Benton MJ (1986) Reptiles from the Upper Kimmeridge Clay (Kimmeridgian, Upper Jurassic) of the vicinity of Egmont Bight, Dorset. Proceedings of the Dorset Natural History and Archaeological Society 107: 121–125.

[138] Billon-Bruyat J-P, Lécuyer C, Martineau F, Mazin J-M (2005) Oxygen isotope compositions of Late Jurassic vertebrate remains from lithographic limestones of western Europe: implications for the ecology of fish, turtles, and crocodilians. Palaeogeography Palaeoclimatology Palaeoecology 216: 359–375.

[139] Buffetaut E (1994) Tetrapods from the Late Jurassic and Early Cretceous lithographic limestones of Europe: a comparative review. Geobios, mémoire spécial 16: 259–265.

[140] Gasparini Z, Fernández M (2005) Jurassic marine reptiles of the Neuquén Basin: records, faunas and their palaeobiogeographic significance. In: Veiga GD, Spalletti LA, Howell JA, Schwarz E, editors. The Neuquén Basin, Argentina: A case study in sequence stratigraphy and basin dynamics. London: Geological Society, special Publications. pp. 279–294.

[141] McGowan C (1991) Dinosaurs, Spitfires, and Sea Dragons: Harvard University Press. 365 p.

[142] Ensom PC, Clements RG, Feist-Burkhardt S, Milner AR, Chitolie J, et al (2009) The age and identity of an ichthyosaur reputedly from the Purbeck Limestone Group, Lower Cretaceous, Dorset, southern England. Cretaceous Research 30: 699–709.

[143] Young MT, Brusatte SL, de Andrade MB, Desojo JB, Beatty BL, et al (2012) The cranial osteology and feeding ecology of the metriorhynchid crocodylomorph genera *Dakosaurus* and *Plesiosuchus* from the Late Jurassic of Europe. PLoS ONE 7: e44985. 23028723 10.1371/journal.pone.0044985 PMC3445579

[144] Owen R (1851–64) A monograph on the fossil Reptilia of the Cretaceous formations. London: The Palæntological Society.

[145] Fischer V, Guiomar M, Godefroit P (2011) New data on the palaeobiogeography of Early Jurassic marine reptiles: the Toarcian ichthyosaur fauna of the Vocontian Basin (SE France). Neues Jahrbuch für Geologie und Paläontologie 261: 111–127.

[146] Benson RB, Ketchum HF, Noè LF, Gómez-Pérez M (2011) New information on Hauffiosaurus (Reptilia, Plesiosauria) based on a new species from the Alumn Shale member (Lower Toarcian: Lower Jurassic) of Yorkshire, UK. Palaeontology 54: 547–571.

[147] Benson RB, Evans M, Druckenmiller PS (2012) High diversity, low disparity and small body size in plesiosaurs (Reptilia, Sauropterygia) from the Triassic–Jurassic boundary. PLoS ONE 7: e31838. 22438869 10.1371/journal.pone.0031838 PMC3306369

[148] Vincent P, Benson RBJ (2012) *Anningasaura*, a basal plesiosaurian (Reptilia, Plesiosauria) from the Lower Jurassic of Lyme Regis, United Kingdom. Journal of Vertebrate Paleontology 32: 1049–1063.

[149] Vincent P, Bardet N, Mattioli E (2013) A new pliosaurid from the Pliensbachian (Early Jurassic) of Normandy (Northern France). Acta Palaeontologica Polonica 58: 471–485.

[150] Vincent P (2008) Les Plesiosauria (Reptilia, Sauropterygia) du Jurassique inférieur : systématique, anatomie, phylogénie et paléoécologie. Paris: Museum national d'Histoire naturelle de Paris. 577 p.

[151] Smith AS (2007) Anatomy and Systematics of the Rhomaleosauridae (Sauropterygia: Plesiosauria): University College Dublin. 278 p.

