# Correction: High Diversity in Cretaceous Ichthyosaurs from Europe Prior to Their Extinction

**DOI:** 10.1371/annotation/3b639689-59a3-4f4a-9ea0-11e9be043382

**Published:** 2014-01-28

**Authors:** Valentin Fischer, Nathalie Bardet, Myette Guiomar, Pascal Godefroit

The images for Figures 17, 18, 19, and 20 were incorrectly switched. The image that appears as Figure 17 should be Figure 19, the image that appears as Figure 18 should be Figure 20, the image that appears as Figure 19 should be Figure 17, and the image that appears as Figure 20 should be Figure 18.

