# Supplementary material for: High Diversity in Cretaceous Ichthyosaurs from Europe Prior to Their Extinction
Source: PLoS One. 2014 Jan 21;9(1):e84709. doi: 10.1371/journal.pone.0084709 (PMC3897400; doi:10.1371/journal.pone.0084709)
Supplement: Text S1 — Gault Formation specimens studied here and their assignation, 19 specimens. (DOC) [file pone.0084709.s001.doc]

**Text S**1. Gault Formation specimens studied here and their assignation, 19 specimens.

| **Specimen** | **Material** | **Assignation** | **Locality** |
| --- | --- | --- | --- |
| **NHMUK unnumbered** | Rostrum | Ichthyosauria indet. | ? |
| **NHMUK 36385** | Mandible elements and phalanges | Aff. *Platypterygius* | Folkestone |
| **NHMUK 36318** | TM1 tooth | ‘*Platypterygius*’ sp*.* | Folkestone |
| **NHMUK 36384** | TM1 tooth | ‘*Platypterygius*’ sp*.* | Folkestone |
| **NHMUK 39256** | Atlas-axis | Ichthyosauria indet. | ? |
| **NHMUK 40095** | TM1 tooth | ‘*Platypterygius*’ sp*.* | ? |
| **NHMUK 47232** | TM2 tooth | *Sisteronia seeleyi* | ? |
| **NHMUK 47235** | Jaw + 12 TM1 teeth | ‘*Platypterygius*’ sp | Folkestone |
| **NHMUK 47271** | TM1 tooth | ‘*Platypterygius*’ sp*.* | ? |
| **NHMUK 47274** | Illium | Ichthyosauria indet. | ? |
| **NHMUK 47275** | Atlas-axis | Ichthyosauria indet. | ? |
| **NHMUK R16 partim** | 7 associated TM2 teeth | *Sisteronia seeleyi* | Folkestone |
| **NHMUK R16 partim** | TM1 tooth | ‘*Platypterygius*’ sp*.* | Folkestone |
| **NHMUK R16 partim** | TM3 tooth | Ophthalmosaurinae indet. | Folkestone |
| **NHMUK R17 partim** | TM2 tooth | *Sisteronia seeleyi* | ? |
| **NHMUK R17 partim** | TM3 tooth | Ophthalmosaurinae indet. | ? |
| **NHMUK R2890 partim** | Quadrate | Ichthyosauria indet. | ? |
| **NHMUK R2890 partim** | Quadrate | Ichthyosauria indet. | ? |
| **NHMUK R2890 partim** | Opisthotic | *Sisteronia seeleyi* | ? |
| **NHMUK R2890 partim** | TM1 tooth | ‘*Platypterygius*’ sp*.* | ? |
