# Supplementary material for: High Diversity in Cretaceous Ichthyosaurs from Europe Prior to Their Extinction
Source: PLoS One. 2014 Jan 21;9(1):e84709. doi: 10.1371/journal.pone.0084709 (PMC3897400; doi:10.1371/journal.pone.0084709)
Supplement: Text S2 — Upper Greensand Formation specimens studied here and their assignation. (DOC) [file pone.0084709.s002.doc]

**Text S2. Upper Greensand Formation specimens studied here and their assignation.**

| **Specimen** | **Material** | **Assignation** | **Locality** |
| --- | --- | --- | --- |
| NHMUK 28110 partim | Tooth (TM1) | ‘*Platypterygius*’ sp. | Cambridge |
| NHMUK 30253 partim | 21 teeth (TM1) | ‘*Platypterygius*’ sp. | Cambridge |
| NHMUK 30253 partim | Tooth (possible TM2) | ? *Sisteronia seeleyi* | Cambridge |
| NHMUK 30254 partim | Tooth (TM1) | ‘*Platypterygius*’ sp. | Cambridge |
| NHMUK 30254 partim | Tooth (TM1) | ‘*Platypterygius*’ sp. | Cambridge |
| NHMUK 30254 partim | Tooth (TM1) | ‘*Platypterygius*’ sp. | Cambridge |
| NHMUK 30254 partim | Tooth (TM1) | ‘*Platypterygius*’ sp. | Cambridge |
| NHMUK 30254 partim | Tooth (TM1) | ‘*Platypterygius*’ sp. | Cambridge |
| NHMUK 30254 partim | Tooth (TM1) | ‘*Platypterygius*’ sp. | Cambridge |
| NHMUK 32406 partim | Tooth (TM1) | ‘*Platypterygius*’ sp. | Kilmerton, Somerset |
| NHMUK 32406 partim | Tooth (TM1) | ‘*Platypterygius*’ sp. | Kilmerton, Somerset |
| NHMUK 35254 | Numerous teeth (TM1) | ‘*Platypterygius*’ sp. | Cambridge |
| NHMUK 33242 | Tooth (TM1) | ‘*Platypterygius*’ sp. | Cambridge |
| NHMUK 46381 | Tooth (TM1) | ‘*Platypterygius*’ sp. | Warminster |
| NHMUK 47269 partim | Tooth (TM1) | ‘*Platypterygius*’ sp. | Folkestone |
| NHMUK 47269 partim | Tooth (TM1) | ‘*Platypterygius*’ sp. | Folkestone |
| NHMUK 47269 partim | Tooth (TM1) | ‘*Platypterygius*’ sp. | Folkestone |
