# Supplementary material for: High Diversity in Cretaceous Ichthyosaurs from Europe Prior to Their Extinction
Source: PLoS One. 2014 Jan 21;9(1):e84709. doi: 10.1371/journal.pone.0084709 (PMC3897400; doi:10.1371/journal.pone.0084709)
Supplement: Text S3 — Cambridge Greensand Member specimens studied here and their assignation. (DOC) [file pone.0084709.s003.doc]

**Text S**3. Cambridge Greensand Member specimens studied here and their assignation.

| **Specimen** | **Material** | **Assignation** | **Locality** |
| --- | --- | --- | --- |
| **CAMSM B20659** | Rostrum | ‘*Platypterygius*’ sp*.* | Cambridge |
| **CAMSM B42404** | Centrum | ‘*Platypterygius*’ sp. | Harston |
| **CAMSM B42405** | Centrum | ‘*Platypterygius*’ sp. | Harston |
| **CAMSM B42406** | Centrum | ‘*Platypterygius*’ sp. | Harston |
| **CAMSM B42407** | Centrum | ‘*Platypterygius*’ sp. | Harston |
| **CAMSM B42408** | Centrum | ‘*Platypterygius*’ sp. | Harston |
| **CAMSM B42409** | Centrum | ‘*Platypterygius*’ sp. | Harston |
| **CAMSM B42410** | Centrum | ‘*Platypterygius*’ sp. | Harston |
| **CAMSM B42411** | Centrum | ‘*Platypterygius*’ sp. | Harston |
| **CAMSM B42412** | Centrum | ‘*Platypterygius*’ sp. | Harston |
| **CAMSM B42413** | Centrum | ‘*Platypterygius*’ sp. | Harston |
| **CAMSM B42414** | Centrum | ‘*Platypterygius*’ sp. | Harston |
| **CAMSM B42415** | Centrum | ‘*Platypterygius*’ sp. | Harston |
| **CAMSM B42416** | Centrum | ‘*Platypterygius*’ sp. | Harston |
| **CAMSM B42417** | Centrum | ‘*Platypterygius*’ sp. | Harston |
| **CAMSM B42418** | Centrum | ‘*Platypterygius*’ sp. | Harston |
| **CAMSM B42420** | Basioccipital (BM1) | ‘*Platypterygius*’ sp. | Harston |
| **CAMSM B57907** | Humerus (HM1) | ‘*Platypterygius*’ sp. | Cambridge |
| **CAMSM B57908** | Opisthotic | *Sisteronia seeleyi* | Cambridge |
| **CAMSM B57939** | Basioccipital (BM1) | ‘*Platypterygius*’ sp. | Cambridge |
| **CAMSM B57940** | Basioccipital (BM1) | ‘*Platypterygius*’ sp. | Cambridge |
| **CAMSM B57941** | Basioccipital (BM2) | *Sisteronia seeleyi* | Cambridge |
| **CAMSM B57942** | Basioccipital (BM3) | Ophthalmosaurinae indet. | Cambridge |
| **CAMSM B57943** | Basioccipital (BM2) | *Sisteronia seeleyi* | Cambridge |
| **CAMSM B57944** | Basioccipital (BM1) | ‘*Platypterygius*’ sp. | Cambridge |
| **CAMSM B57945** | Basioccipital (BM2) | *Sisteronia seeleyi* | Cambridge |
| **CAMSM B57946** | Basioccipital (BM2) | *Sisteronia seeleyi* | Cambridge |
| **CAMSM B57947** | Basioccipital (BM2) | *Sisteronia seeleyi* | Cambridge |
| **CAMSM B57948** | Basioccipital (BM2) | *Sisteronia seeleyi* | Cambridge |
| **CAMSM B57949** | Basioccipital (BM3) | Ophthalmosaurinae indet. | Cambridge |
| **CAMSM B57950** | Basioccipital (BM2) | *Sisteronia seeleyi* | Cambridge |
| **CAMSM B57951** | Basioccipital (BM2) | *Sisteronia seeleyi* | Cambridge |
| **CAMSM B57952** | Basioccipital (BM3) | Ophthalmosaurinae indet. | Cambridge |
| **CAMSM B57954** | Basioccipital (BM2) | *Sisteronia seeleyi* | Cambridge |
| **CAMSM B57955** | Basioccipital (BM3) | Ophthalmosaurinae indet. | Cambridge |
| **CAMSM B57956** | Basioccipital (BM2) | *Sisteronia seeleyi* | Cambridge |
| **CAMSM B57959_60*** | Basioccipital (BM1), Atlas-axis | ‘*Platypterygius*’ sp. | Cambridge |
| **CAMSM B57961** | Basioccipital (BM3) | Ophthalmosaurinae indet. | Cambridge |
| **CAMSM B57962** | Basisphenoid | Ichthyosauria indet. | Cambridge |
| **CAMSM B57963** | Basisphenoid | Ophthalmosauridae indet. | Cambridge |
| **CAMSM B57964** | Basisphenoid | Ophthalmosauridae indet. | Cambridge |
| **CAMSM B57965** | Basisphenoid | Ophthalmosauridae indet. | Cambridge |
| **CAMSM B57966** | Basisphenoid | Ophthalmosauridae indet. | Cambridge |
| **CAMSM B57967** | Basisphenoid | Ophthalmosauridae indet. | Cambridge |
| **CAMSM B57987** | HM1 humerus | ‘*Platypterygius*’ sp. | Cambridge |
| **CAMSM B57988** | Left quadrate | Ophthalmosauridae indet. | Cambridge |
| **CAMSM B57989** | Left quadrate | Ophthalmosauridae indet. | Cambridge |
| **CAMSM B57990** | Left quadrate | Ophthalmosauridae indet. | Cambridge |
| **CAMSM B57991** | Left quadrate | Ophthalmosauridae indet. | Cambridge |
| **CAMSM B57995** | Left exoccipital | Ophthalmosauridae indet. | Cambridge |
| **CAMSM B57996** | Tooth | ‘*Platypterygius*’ sp. | Cambridge |
| **CAMSM B57997** | Tooth | ‘*Platypterygius*’ sp. | Cambridge |
| **CAMSM B57998** | Tooth | ‘*Platypterygius*’ sp. | Cambridge |
| **CAMSM B57999** | Tooth | ‘*Platypterygius*’ sp. | Cambridge |
| **CAMSM B58000** | Tooth | ‘*Platypterygius*’ sp. | Cambridge |
| **CAMSM B58001** | Tooth | ‘*Platypterygius*’ sp. | Cambridge |
| **CAMSM B58002** | Tooth | ‘*Platypterygius*’ sp. | Cambridge |
| **CAMSM B58003** | Tooth | ‘*Platypterygius*’ sp. | Cambridge |
| **CAMSM B58004** | Tooth | ‘*Platypterygius*’ sp. | Cambridge |
| **CAMSM B58005** | Tooth | ‘*Platypterygius*’ sp. | Cambridge |
| **CAMSM B58006** | Tooth | ‘*Platypterygius*’ sp. | Cambridge |
| **CAMSM B58007** | Tooth | ‘*Platypterygius*’ sp. | Cambridge |
| **CAMSM B58008** | Tooth | ‘*Platypterygius*’ sp. | Cambridge |
| **CAMSM B58009** | Tooth | ‘*Platypterygius*’ sp. | Cambridge |
| **CAMSM B58010** | Tooth | ‘*Platypterygius*’ sp. | Cambridge |
| **CAMSM B58011** | Tooth | ‘*Platypterygius*’ sp. | Cambridge |
| **CAMSM B58012** | Tooth | ‘*Platypterygius*’ sp. | Cambridge |
| **CAMSM B58013** | Tooth | ‘*Platypterygius*’ sp. | Cambridge |
| **CAMSM B58014** | Tooth | ‘*Platypterygius*’ sp. | Cambridge |
| **CAMSM B58015** | Tooth | ‘*Platypterygius*’ sp. | Cambridge |
| **CAMSM B58016** | Tooth | ‘*Platypterygius*’ sp. | Cambridge |
| **CAMSM B58017** | Tooth | ‘*Platypterygius*’ sp. | Cambridge |
| **CAMSM B58018** | Tooth | ‘*Platypterygius*’ sp. | Cambridge |
| **CAMSM B58019** | Tooth | ‘*Platypterygius*’ sp. | Cambridge |
| **CAMSM B58020** | Tooth | ‘*Platypterygius*’ sp. | Cambridge |
| **CAMSM B58021** | Tooth | ‘*Platypterygius*’ sp. | Cambridge |
| **CAMSM B58022** | Tooth | ‘*Platypterygius*’ sp. | Cambridge |
| **CAMSM B58023** | Tooth | ‘*Platypterygius*’ sp. | Cambridge |
| **CAMSM B58024** | Tooth | ‘*Platypterygius*’ sp. | Cambridge |
| **CAMSM B58025** | Tooth | ‘*Platypterygius*’ sp. | Cambridge |
| **CAMSM B58026** | Tooth | ‘*Platypterygius*’ sp. | Cambridge |
| **CAMSM B58027** | Tooth | ‘*Platypterygius*’ sp. | Cambridge |
| **CAMSM B58030** | Premaxilla with 13 teeth | ‘*Platypterygius*’ sp. | Cambridge |
| **CAMSM B58040** | Illium | Ichthyosauria indet. | Cambridge |
| **CAMSM B58042** | Humerus (HM4) | Ophthalmosaurinae indet. | Cambridge |
| **CAMSM B58043** | Humerus (HM5) | ‘*Platypterygius*’ sp. | Cambridge |
| **CAMSM B58044** | Humerus | Ophthalmosauridae indet. | Cambridge |
| **CAMSM B58045** | Humerus (HM4) | Ophthalmosaurinae indet. | Cambridge |
| **CAMSM B58046** | Humerus | Ophthalmosauridae indet. | Cambridge |
| **CAMSM B58047** | Humerus | Ophthalmosauridae indet. | Cambridge |
| **CAMSM B58048** | Humerus (HM5) | ‘*Platypterygius*’ sp. | Cambridge |
| **CAMSM B58050** | Humerus (HM4) | Ophthalmosaurinae indet. | Cambridge |
| **CAMSM B58051** | Humerus | Ophthalmosauridae indet. | Cambridge |
| **CAMSM B58052** | Humerus | Ophthalmosauridae indet. | Cambridge |
| **CAMSM B58053** | Humerus (HM4) | Ophthalmosaurinae indet. | Cambridge |
| **CAMSM B58054** | Humerus | Ophthalmosauridae indet. | Cambridge |
| **CAMSM B58055** | Humerus (HM4) | Ophthalmosaurinae indet. | Cambridge |
| **CAMSM B58056** | Humerus (HM1) | ‘*Platypterygius*’ sp. | Cambridge |
| **CAMSM B58057** | Humerus (HM1) | ‘*Platypterygius*’ sp. | Cambridge |
| **CAMSM B58058** | Femur (FM1) | ‘*Platypterygius*’ sp. | Cambridge |
| **CAMSM B58059** | Femur (FM3) | Ophthalmosauridae indet. | Cambridge |
| **CAMSM B58060** | Femur (FM1) | ‘*Platypterygius*’ sp. | Cambridge |
| **CAMSM B58061** | Femur (FM2) | Ophthalmosauridae indet. | Cambridge |
| **CAMSM B58062** | Femur (FM1) | ‘*Platypterygius*’ sp. | Cambridge |
| **CAMSM B58063** | Femur (FM1) | ‘*Platypterygius*’ sp. | Cambridge |
| **CAMSM B58064** | Femur (FM1) | ‘*Platypterygius*’ sp. | Cambridge |
| **CAMSM B58065** | Femur (FM4) | Ophthalmosauridae indet. | Cambridge |
| **CAMSM B58066** | Femur (FM2) | Ophthalmosauridae indet. | Cambridge |
| **CAMSM B58067** | Femur (FM2) | Ophthalmosauridae indet. | Cambridge |
| **CAMSM B58068** | Femur (FM2) | Ophthalmosauridae indet. | Cambridge |
| **CAMSM B58069** | Propodial (FM5) | *Cetarthrosaurus walkeri* | Cambridge (N.E.) |
| **CAMSM B58070** | Coracoid | Ophthalmosauridae indet. | Cambridge |
| **CAMSM B58071** | Coracoid | Ophthalmosauridae indet. | Cambridge |
| **CAMSM B58072** | Coracoid | Ophthalmosauridae indet. | Cambridge |
| **CAMSM B58073** | Coracoid | Ophthalmosauridae indet. | Cambridge |
| **CAMSM B58074** | Stapes | Ophthalmosaurinae indet. | Cambridge |
| **CAMSM B58075** | Stapes | Ophthalmosaurinae indet. | Cambridge |
| **CAMSM B58076** | Stapes | Ichthyosauria indet. | Cambridge |
| **CAMSM B58077** | Opisthotic | *Sisteronia seeleyi* | Cambridge |
| **CAMSM B58078** | Opisthotic | *Sisteronia seeleyi* | Cambridge |
| **CAMSM B58079** | Stapes | Ophthalmosaurinae indet. | Cambridge |
| **CAMSM B58227** | Postflexural centrum | Ichthyosauria indet. | Cambridge |
| **CAMSM B58228** | Postflexural centrum | Ichthyosauria indet. | Cambridge |
| **CAMSM B58229** | Postflexural centrum | Ichthyosauria indet. | Cambridge |
| **CAMSM B58230** | Postflexural centrum | Ichthyosauria indet. | Cambridge |
| **CAMSM B58231** | Postflexural centrum | Ichthyosauria indet. | Cambridge |
| **CAMSM B58232** | Postflexural centrum | Ichthyosauria indet. | Cambridge |
| **CAMSM B58233** | Postflexural centrum | Ichthyosauria indet. | Cambridge |
| **CAMSM B58234** | Postflexural centrum | Ichthyosauria indet. | Cambridge |
| **CAMSM B58235** | Postflexural centrum | Ichthyosauria indet. | Cambridge |
| **CAMSM B58236** | Postflexural centrum | Ichthyosauria indet. | Cambridge |
| **CAMSM B58237** | Postflexural centrum | Ichthyosauria indet. | Cambridge |
| **CAMSM B58238** | Postflexural centrum | Ichthyosauria indet. | Cambridge |
| **CAMSM B58239** | Postflexural centrum | Ichthyosauria indet. | Cambridge |
| **CAMSM B58240** | Postflexural centrum | Ichthyosauria indet. | Cambridge |
| **CAMSM B58241** | Postflexural centrum | Ichthyosauria indet. | Cambridge |
| **CAMSM B58242** | Postflexural centrum | Ichthyosauria indet. | Cambridge |
| **CAMSM B58243** | Postflexural centrum | Ichthyosauria indet. | Cambridge |
| **CAMSM B58244** | Postflexural centrum | Ichthyosauria indet. | Cambridge |
| **CAMSM B58245** | Postflexural centrum | Ichthyosauria indet. | Cambridge |
| **CAMSM B58246** | Postflexural centrum | Ichthyosauria indet. | Cambridge |
| **CAMSM B58247** | Postflexural centrum | Ichthyosauria indet. | Cambridge |
| **CAMSM B58248** | Postflexural centrum | Ichthyosauria indet. | Cambridge |
| **CAMSM B58249** | Postflexural centrum | Ichthyosauria indet. | Cambridge |
| **CAMSM B58250_56*** | Incomplete skeleton | ‘*Platypterygius*’ sp. | Cambridge |
| **CAMSM B58257_67*** | Incomplete skeleton | *Sisteronia seeleyi* | Cambridge |
| **CAMSM B58294** | Intermedium | Ichthyosauria indet. | Cambridge |
| **CAMSM B58295** | Intermedium | Ichthyosauria indet. | Cambridge |
| **CAMSM B58296** | Radius or ulna | Ophthalmosauridae indet. | Cambridge |
| **CAMSM B58297** | Radius or ulna | Ophthalmosauridae indet. | Cambridge |
| **CAMSM B58298** | Radius, intermedium or ulna | Ophthalmosauridae indet. | Cambridge |
| **CAMSM B58299** | Radius or ulna | Ophthalmosauridae indet. | Cambridge |
| **CAMSM B58300** | Radius or ulna | Ophthalmosauridae indet. | Cambridge |
| **CAMSM B58301** | Zeugopodial element | Ichthyosauria indet. | Cambridge |
| **CAMSM B58302** | Zeugopodial element | Ichthyosauria indet. | Cambridge |
| **CAMSM B58303** | Radius or ulna | Ophthalmosauridae indet. | Cambridge |
| **CAMSM B58304** | Radius or ulna | Ophthalmosauridae indet. | Cambridge |
| **CAMSM B58305** | Tooth (TM1) | ‘*Platypterygius*’ sp. | Cambridge |
| **CAMSM B58306** | Tooth (TM1) | ‘*Platypterygius*’ sp. | Cambridge |
| **CAMSM B58307** | Tooth (TM1) | ‘*Platypterygius*’ sp. | Cambridge |
| **CAMSM B58308** | Tooth (TM1) | ‘*Platypterygius*’ sp. | Cambridge |
| **CAMSM B58309** | Tooth (TM1) | ‘*Platypterygius*’ sp. | Cambridge |
| **CAMSM B58310** | Tooth (TM1) | ‘*Platypterygius*’ sp. | Cambridge |
| **CAMSM B58311** | Tooth (TM1) | ‘*Platypterygius*’ sp. | Cambridge |
| **CAMSM B58312** | Tooth (TM1) | ‘*Platypterygius*’ sp. | Cambridge |
| **CAMSM B58313** | Tooth (TM1) | ‘*Platypterygius*’ sp. | Cambridge |
| **CAMSM B58314** | Basioccipital (BM2) | *Sisteronia seeleyi* | Cambridge |
| **CAMSM B58315** | Atlas-axis | Ichthyosauria indet. | Cambridge |
| **CAMSM B58316** | Cervical centrum | Ichthyosauria indet. | Cambridge |
| **CAMSM B58317** | Cervical centrum | Ichthyosauria indet. | Cambridge |
| **CAMSM B58318** | Cervical centrum | Ichthyosauria indet. | Cambridge |
| **CAMSM B58319** | Cervical centrum | Ichthyosauria indet. | Cambridge |
| **CAMSM B58320** | Cervical centrum | Ichthyosauria indet. | Cambridge |
| **CAMSM B58321** | Cervical centrum | Ichthyosauria indet. | Cambridge |
| **CAMSM B58322** | Cervical centrum | Ichthyosauria indet. | Cambridge |
| **CAMSM B58323** | Cervical centrum | Ichthyosauria indet. | Cambridge |
| **CAMSM B58324** | Cervical centrum | Ichthyosauria indet. | Cambridge |
| **CAMSM B58325** | Dorsal centrum | Ichthyosauria indet. | Cambridge |
| **CAMSM B58326** | Dorsal centrum | Ichthyosauria indet. | Cambridge |
| **CAMSM B58327** | Dorsal centrum | Ichthyosauria indet. | Cambridge |
| **CAMSM B58328** | Dorsal centrum | Ichthyosauria indet. | Cambridge |
| **CAMSM B58329** | Dorsal centrum | Ichthyosauria indet. | Cambridge |
| **CAMSM B58330** | Caudal centrum | Ichthyosauria indet. | Cambridge |
| **CAMSM B58331** | Caudal centrum | Ichthyosauria indet. | Cambridge |
| **CAMSM B58332** | Caudal centrum | Ichthyosauria indet. | Cambridge |
| **CAMSM B58333** | Caudal centrum | Ichthyosauria indet. | Cambridge |
| **CAMSM B58334** | Caudal centrum | Ichthyosauria indet. | Cambridge |
| **CAMSM B58335** | Caudal centrum | Ichthyosauria indet. | Cambridge |
| **CAMSM B58336** | Caudal centrum | Ichthyosauria indet. | Cambridge |
| **CAMSM B58337** | Atlas-axis | Ichthyosauria indet. | Cambridge |
| **CAMSM B58338** | Cervical centrum | Ichthyosauria indet. | Cambridge |
| **CAMSM B58339** | Cervical centrum | Ichthyosauria indet. | Cambridge |
| **CAMSM B58340** | Cervical centrum | Ichthyosauria indet. | Cambridge |
| **CAMSM B58341** | Cervical centrum | Ichthyosauria indet. | Cambridge |
| **CAMSM B58342** | Cervical centrum | Ichthyosauria indet. | Cambridge |
| **CAMSM B58343** | Dorsal centrum | Ichthyosauria indet. | Cambridge |
| **CAMSM B58344** | Dorsal centrum | Ichthyosauria indet. | Cambridge |
| **CAMSM B58345** | Dorsal centrum | Ichthyosauria indet. | Cambridge |
| **CAMSM B58346** | Dorsal centrum | Ichthyosauria indet. | Cambridge |
| **CAMSM B58347** | Dorsal centrum | Ichthyosauria indet. | Cambridge |
| **CAMSM B58348** | Dorsal centrum | Ichthyosauria indet. | Cambridge |
| **CAMSM B58349** | Dorsal centrum | Ichthyosauria indet. | Cambridge |
| **CAMSM B58350** | Dorsal centrum | Ichthyosauria indet. | Cambridge |
| **CAMSM B58351** | Dorsal centrum | Ichthyosauria indet. | Cambridge |
| **CAMSM B58352** | Dorsal centrum | Ichthyosauria indet. | Cambridge |
| **CAMSM B58353** | Dorsal centrum | Ichthyosauria indet. | Cambridge |
| **CAMSM B58354** | Caudal centrum | Ichthyosauria indet. | Cambridge |
| **CAMSM B58355** | Dorsal centrum | Ichthyosauria indet. | Cambridge |
| **CAMSM B58356** | Caudal centrum | Ichthyosauria indet. | Cambridge |
| **CAMSM B58357** | Caudal centrum | Ichthyosauria indet. | Cambridge |
| **CAMSM B58358** | Caudal centrum | Ichthyosauria indet. | Cambridge |
| **CAMSM B58359** | Caudal centrum | Ichthyosauria indet. | Cambridge |
| **CAMSM B58360** | Caudal centrum | Ichthyosauria indet. | Cambridge |
| **CAMSM B58361** | Femur (FM1) | ‘*Platypterygius*’ sp. | Cambridge |
| **CAMSM B58374** | Coracoid | Ichthyosauria indet. | Cambridge |
| **CAMSM B58379** | Tooth (TM1) | ‘*Platypterygius*’ sp. | Cambridge |
| **CAMSM B58380** | Tooth (TM1) | ‘*Platypterygius*’ sp. | Cambridge |
| **CAMSM B58381** | Tooth (TM1) | ‘*Platypterygius*’ sp. | Cambridge |
| **CAMSM B58382** | Tooth (TM1) | ‘*Platypterygius*’ sp. | Cambridge |
| **CAMSM B58383** | Tooth (TM1) | ‘*Platypterygius*’ sp. | Cambridge |
| **CAMSM B58384** | Tooth (TM1) | ‘*Platypterygius*’ sp. | Cambridge |
| **CAMSM B58385** | Tooth (TM1) | ‘*Platypterygius*’ sp. | Cambridge |
| **CAMSM B58386** | Tooth (TM1) | ‘*Platypterygius*’ sp. | Cambridge |
| **CAMSM B58387** | Tooth (TM1) | ‘*Platypterygius*’ sp. | Cambridge |
| **CAMSM B58388** | Tooth (TM1) | ‘*Platypterygius*’ sp. | Cambridge |
| **CAMSM B58389** | Tooth | Ichthyosauria indet. | Cambridge |
| **CAMSM B58390** | Tooth | Ichthyosauria indet. | Cambridge |
| **CAMSM B58391** | Tooth (TM2) | *Sisteronia seeleyi* | Cambridge |
| **CAMSM B58392** | Tooth | Ichthyosauria indet. | Cambridge |
| **CAMSM B58393** | Tooth | Ichthyosauria indet. | Cambridge |
| **CAMSM B58394** | Tooth (TM2) | *Sisteronia seeleyi* | Cambridge |
| **CAMSM B58395** | Tooth (TM1) | ‘*Platypterygius*’ sp. | Cambridge |
| **CAMSM B58396** | Tooth (TM1) | ‘*Platypterygius*’ sp. | Cambridge |
| **CAMSM B58397** | Tooth (TM1) | ‘*Platypterygius*’ sp. | Cambridge |
| **CAMSM B58398** | Tooth (TM1) | ‘*Platypterygius*’ sp. | Cambridge |
| **CAMSM B58399** | Tooth (TM1) | ‘*Platypterygius*’ sp. | Cambridge |
| **CAMSM B58400** | Tooth (TM1) | ‘*Platypterygius*’ sp. | Cambridge |
| **CAMSM B76728** | Tooth (TM1) | ‘*Platypterygius*’ sp. | Madingley |
| **CAMSM B76729** | Tooth (TM1) | ‘*Platypterygius*’ sp. | Madingley |
| **CAMSM B76730** | Tooth (TM1) | ‘*Platypterygius*’ sp. | Madingley |
| **CAMSM B76731** | Tooth (TM1) | ‘*Platypterygius*’ sp. | Madingley |
| **CAMSM B76732** | Tooth (TM1) | ‘*Platypterygius*’ sp. | Madingley |
| **CAMSM B76733** | Tooth (TM1) | ‘*Platypterygius*’ sp. | Madingley |
| **CAMSM B76734** | Tooth (TM1) | ‘*Platypterygius*’ sp. | Madingley |
| **CAMSM B76735** | Tooth (TM1) | ‘*Platypterygius*’ sp. | Madingley |
| **CAMSM B76736** | Tooth (TM1) | ‘*Platypterygius*’ sp. | Madingley |
| **CAMSM B76737** | Tooth (TM1) | ‘*Platypterygius*’ sp. | Madingley |
| **CAMSM B76738** | Tooth (TM1) | ‘*Platypterygius*’ sp. | Madingley |
| **CAMSM B76739** | Tooth (TM1) | ‘*Platypterygius*’ sp. | Madingley |
| **CAMSM B76740** | Tooth (TM1) | ‘*Platypterygius*’ sp. | Madingley |
| **CAMSM B76741** | Tooth (TM1) | ‘*Platypterygius*’ sp. | Madingley |
| **CAMSM B76742** | Tooth (TM1) | ‘*Platypterygius*’ sp. | Madingley |
| **CAMSM B76743** | Tooth (TM1) | ‘*Platypterygius*’ sp. | Madingley |
| **CAMSM B76744** | Tooth (TM1) | ‘*Platypterygius*’ sp. | Madingley |
| **CAMSM B76745** | Tooth (TM1) | ‘*Platypterygius*’ sp. | Madingley |
| **CAMSM B97401 partim** | Complete manus + epipodium | ‘*Platypterygius*’ sp. | Cambridge |
| **CAMSM B97401 partim** | Humerus (HM1) | ‘*Platypterygius*’ sp. | Cambridge |
| **CAMSM TN1716 partim** | Teeth | *Sisteronia seeleyi* and *‘Platypterygius’ sp.* | Cambridgeshire |
| **CAMSM TN1722 partim** | Basisphenoid | Ichthyosauria indet. | Cambridgeshire |
| **CAMSM TN1722 partim** | Basisphenoid | Ichthyosauria indet. | Cambridgeshire |
| **CAMSM TN1727 partim** | Basioccipital (BM2) | *Sisteronia seeleyi* | Cambridgeshire |
| **CAMSM TN1727 partim** | Basisphenoid | Ichthyosauria indet. | Cambridgeshire |
| **CAMSM TN1727 partim** | Humerus (HM4) | Ophthalmosaurinae indet. | Cambridgeshire |
| **CAMSM TN1729 partim** | Basioccipital (BM1) | ‘*Platypterygius*’ sp. | Cambridgeshire |
| **CAMSM TN1734 partim** | Stapes | Ophthalmosauridae indet. | Cambridgeshire |
| **CAMSM TN1734 partim** | Humerus (HM5) | ‘*Platypterygius*’ sp. | Cambridgeshire |
| **CAMSM TN1735 partim** | Teeth (TM1) | ‘*Platypterygius*’ sp. | Cambridgeshire |
| **CAMSM TN1735 partim** | Basioccipital (BM2) | *Sisteronia seeleyi* | Cambridgeshire |
| **CAMSM TN1735 partim** | Basioccipital (BM2) | *Sisteronia seeleyi* | Cambridgeshire |
| **CAMSM TN1735 partim** | Basioccipital (BM2) | *Sisteronia seeleyi* | Cambridgeshire |
| **CAMSM TN1735 partim** | Basioccipital (BM2) | *Sisteronia seeleyi* | Cambridgeshire |
| **CAMSM TN1735 partim** | Basioccipital (BM2) | *Sisteronia seeleyi* | Cambridgeshire |
| **CAMSM TN1735 partim** | Basioccipital (BM2) | *Sisteronia seeleyi* | Cambridgeshire |
| **CAMSM TN1735 partim** | Basioccipital (BM3) | Ophthalmosaurinae indet. | Cambridgeshire |
| **CAMSM TN1735 partim** | Basioccipital (BM3) | Ophthalmosaurinae indet. | Cambridgeshire |
| **CAMSM TN1735 partim** | Basioccipital (BM3) | Ophthalmosaurinae indet. | Cambridgeshire |
| **CAMSM TN1735 partim** | Basioccipital (BM3) | Ophthalmosaurinae indet. | Cambridgeshire |
| **CAMSM TN1735 partim** | Basioccipital (BM3) | Ophthalmosaurinae indet. | Cambridgeshire |
| **CAMSM TN1735 partim** | Basioccipital (BM3) | Ophthalmosaurinae indet. | Cambridgeshire |
| **CAMSM TN1739 partim** | Stapes | Ophthalmosauridae indet. | Cambridgeshire |
| **CAMSM TN1739 partim** | Basioccipital (BM2) | *Sisteronia seeleyi* | Cambridgeshire |
| **CAMSM TN1739 partim** | Centra | Ichthyosauria indet. | Cambridgeshire |
| **CAMSM TN1748 partim** | Femur (FM1) | ‘*Platypterygius*’ sp. | Cambridgeshire |
| **CAMSM TN1751 partim** | Basisphenoid | Ichthyosauria indet. | Cambridgeshire |
| **CAMSM TN1751 partim** | Humerus (HM1) | ‘*Platypterygius*’ sp. | Cambridgeshire |
| **CAMSM TN1751 partim** | Basioccipital (BM2) | *Sisteronia seeleyi* | Cambridgeshire |
| **CAMSM TN1751 partim** | Basioccipital (BM2) | *Sisteronia seeleyi* | Cambridgeshire |
| **CAMSM TN1751 partim** | Basioccipital (BM2) | *Sisteronia seeleyi* | Cambridgeshire |
| **CAMSM TN1751 partim** | Basioccipital (BM2) | *Sisteronia seeleyi* | Cambridgeshire |
| **CAMSM TN1751 partim** | Basioccipital (BM2) | *Sisteronia seeleyi* | Cambridgeshire |
| **CAMSM TN1751 partim** | Basioccipital (BM2) | *Sisteronia seeleyi* | Cambridgeshire |
| **CAMSM TN1751 partim** | Basioccipital (BM3) | Ophthalmosaurinae indet. | Cambridgeshire |
| **CAMSM TN1751 partim** | Basioccipital (BM3) | Ophthalmosaurinae indet. | Cambridgeshire |
| **CAMSM TN1751 partim** | Basioccipital (BM3) | Ophthalmosaurinae indet. | Cambridgeshire |
| **CAMSM TN1751 partim** | Basioccipital (BM3) | Ophthalmosaurinae indet. | Cambridgeshire |
| **CAMSM TN1753 partim** | Basisphenoid | Ichthyosauria indet. | Cambridgeshire |
| **CAMSM TN1753 partim** | Opisthotic | *Sisteronia seeleyi* | Cambridgeshire |
| **CAMSM TN1753 partim** | Humerus (HM1) | ‘*Platypterygius*’ sp. | Cambridgeshire |
| **CAMSM TN1753 partim** | Humerus (HM5) | ‘*Platypterygius*’ sp. | Cambridgeshire |
| **CAMSM TN1753 partim** | Basioccipital (BM2) | *Sisteronia seeleyi* | Cambridgeshire |
| **CAMSM TN1753 partim** | Basioccipital (BM3) | Ophthalmosaurinae indet. | Cambridgeshire |
| **CAMSM TN1753 partim** | Basioccipital (BM3) | Ophthalmosaurinae indet. | Cambridgeshire |
| **CAMSM TN1754 partim** | Basioccipital (BM1) | ‘*Platypterygius*’ sp. | Cambridgeshire |
| **CAMSM TN1755 partim** | Basioccipital (BM1) | ‘*Platypterygius*’ sp. | Cambridgeshire |
| **CAMSM TN1755 partim** | Basioccipital (BM1) | ‘*Platypterygius*’ sp. | Cambridgeshire |
| **CAMSM TN1755 partim** | Basioccipital (BM3) | Ophthalmosaurinae indet. | Cambridgeshire |
| **CAMSM TN1755 partim** | Basisphenoid | Ichthyosauria indet. | Cambridgeshire |
| **CAMSM TN1755 partim** | Basisphenoid | Ichthyosauria indet. | Cambridgeshire |
| **CAMSM TN1755 partim** | Basisphenoid | Ichthyosauria indet. | Cambridgeshire |
| **CAMSM TN1755 partim** | Humerus (HM2) | *Sisteronia seeleyi* | Cambridgeshire |
| **CAMSM TN1755 partim** | Humerus (HM4) | Ophthalmosaurinae indet. | Cambridgeshire |
| **CAMSM TN1755 partim** | Humerus (HM4) | Ophthalmosaurinae indet. | Cambridgeshire |
| **CAMSM TN1756 partim** | Quadrate | Ichthyosauria indet. | Cambridgeshire |
| **CAMSM TN1756 partim** | Basisphenoid | Ichthyosauria indet. | Cambridgeshire |
| **CAMSM TN1756 partim** | Basisphenoid | Ichthyosauria indet. | Cambridgeshire |
| **CAMSM TN1756 partim** | Humerus | Ophthalmosauridae indet. | Cambridgeshire |
| **CAMSM TN1756 partim** | Humerus | Ophthalmosauridae indet. | Cambridgeshire |
| **CAMSM TN1756 partim** | Centra | Ichthyosauria indet. | Cambridgeshire |
| **CAMSM TN1757 partim** | Exoccipital | Ichthyosauria indet. | Cambridgeshire |
| **CAMSM TN1757 partim** | Stapes | Ophthalmosaurinae indet. | Cambridgeshire |
| **CAMSM TN1757 partim** | Humerus (HM2) | *Sisteronia seeleyi* | Cambridgeshire |
| **CAMSM TN1757 partim** | Humerus (HM5) | ‘*Platypterygius*’ sp. | Cambridgeshire |
| **CAMSM TN1757 partim** | Femur (FM1) | ‘*Platypterygius*’ sp. | Cambridgeshire |
| **CAMSM TN1757 partim** | Femur (FM1) | ‘*Platypterygius*’ sp. | Cambridgeshire |
| **CAMSM TN1758 partim** | Quadrate | Ichthyosauria indet. | Cambridgeshire |
| **CAMSM TN1758 partim** | Humerus | Ophthalmosauridae indet. | Cambridgeshire |
| **CAMSM TN1778 partim** | Teeth (≈300) | *Sisteronia seeleyi* and *‘Platypterygius’ sp.* | Cambridgeshire |
| **CAMSM TN1779 partim** | Teeth | *Sisteronia seeleyi* and *‘Platypterygius’ sp.* | Cambridgeshire |
| **CAMSM TN1779 partim** | Basisphenoid | Ichthyosauria indet. | Cambridgeshire |
| **CAMSM TN282** | Rostrum | ‘*Platypterygius*’ sp. | Cambridgeshire |
| **CAMSM TN283** | Rostrum | ‘*Platypterygius*’ sp. | Cambridgeshire |
| **CAMSM X50161** | Basioccipital (BM1) | ‘*Platypterygius*’ sp. | Cambridgeshire |
| **CAMSM X50168** | Basioccipital (BM1) | ‘*Platypterygius*’ sp. | Cambridgeshire |
| **CAMSM X50169** | Basioccipital (BM1) | ‘*Platypterygius*’ sp. | Bottisham Lode, east Cambridgeshire |
| **CAMSM X50170** | Propodial (FM5) | Cetarthrosaurus walkeri | Cambridgeshire |
| **GLAHM V.1463** | Basioccipital (BM3) | Ophthalmosaurinae indet. | New Market road pits |
| **GLAHM V.1535/1** | Stapes | Ophthalmosaurinae indet. | Cambridgeshire |
| **IRSNB GS1** | Quadrate | Ichthyosauria indet. | Cambridge |
| **IRSNB GS2** | Quadrate | Ichthyosauria indet. | Cambridge |
| **IRSNB GS3** | Humerus (HM4) | Ophthalmosaurinae indet. | Cambridge |
| **IRSNB GS4** | Quadrate | Ichthyosauria indet. | Cambridge |
| **IRSNB GS5** | Scapula | Baracromia indet. | Cambridge |
| **IRSNB GS6** | Quadrate | Ichthyosauria indet. | Cambridge |
| **IRSNB GS7** | Articular | Ichthyosauria indet. | Cambridge |
| **IRSNB GS8** | Quadrate | Ichthyosauria indet. | Cambridge |
| **IRSNB GS9** | Basisphenoid | Ichthyosauria indet. | Cambridge |
| **IRSNB GS10** | Opisthotic | *Sisteronia seeleyi* | Cambridge |
| **IRSNB GS11** | Quadrate | Ichthyosauria indet. | Cambridge |
| **IRSNB GS12** | Femur | Ichthyosauria indet. | Cambridge |
| **IRSNB GS13** | Exoccipital | Ichthyosauria indet. | Cambridge |
| **IRSNB GS14** | Interclavicle | Ichthyosauria indet. | Cambridge |
| **IRSNB GS15** | Atlas-axis | Ichthyosauria indet. | Cambridge |
| **IRSNB GS16** | Cervical centrum | Ichthyosauria indet. | Cambridge |
| **IRSNB GS17** | Caudal centrum | Ichthyosauria indet. | Cambridge |
| **IRSNB GS18** | Quadrate | Ichthyosauria indet. | Cambridge |
| **IRSNB GS19** | Humerus | Ophthalmosauridae indet. | Cambridge |
| **IRSNB GS20** | Humerus | Ophthalmosauridae indet. | Cambridge |
| **IRSNB GS21** | Tooth (TM1) | ‘*Platypterygius*’ sp. | Cambridge |
| **IRSNB GS22** | Tooth (TM1) | ‘*Platypterygius*’ sp. | Cambridge |
| **IRSNB GS23** | Tooth (TM2) | *Sisteronia seeleyi* | Cambridge |
| **IRSNB GS24** | Tooth (TM2) | *Sisteronia seeleyi*. | Cambridge |
| **IRSNB GS25** | Tooth (TM1) | ‘*Platypterygius*’ sp. | Cambridge |
| **IRSNB GS26** | Tooth (TM1) | ‘*Platypterygius*’ sp. | Cambridge |
| **IRSNB GS27** | Tooth (TM1) | ‘*Platypterygius*’ sp. | Cambridge |
| **IRSNB GS28** | Tooth (TM1) | ‘*Platypterygius*’ sp. | Cambridge |
| **IRSNB GS29** | Tooth juvenile | Ichthyosauria indet. | Cambridge |
| **IRSNB GS30** | Tooth juvenile | Ichthyosauria indet. | Cambridge |
| **IRSNB GS31** | Tooth juvenile | Ichthyosauria indet. | Cambridge |
| **IRSNB GS32** | Tooth (TM1) | ‘*Platypterygius*’ sp. | Cambridge |
| **IRSNB GS33** | Tooth (TM1) | ‘*Platypterygius*’ sp. | Cambridge |
| **IRSNB GS34** | Tooth (TM1) | ‘*Platypterygius*’ sp. | Cambridge |
| **IRSNB GS35** | Tooth (TM1) | ‘*Platypterygius*’ sp. | Cambridge |
| **IRSNB GS36** | Tooth (TM1) | ‘*Platypterygius*’ sp. | Cambridge |
| **IRSNB GS37** | Tooth (TM1) | ‘*Platypterygius*’ sp. | Cambridge |
| **IRSNB GS38** | Tooth (TM1) | ‘*Platypterygius*’ sp. | Cambridge |
| **IRSNB GS39** | Tooth (TM1) | ‘*Platypterygius*’ sp. | Cambridge |
| **IRSNB GS40** | Tooth (TM1) | ‘*Platypterygius*’ sp. | Cambridge |
| **IRSNB GS41** | Tooth (TM1) | ‘*Platypterygius*’ sp. | Cambridge |
| **IRSNB GS42** | Tooth (TM1) | ‘*Platypterygius*’ sp. | Cambridge |
| **IRSNB GS43** | Tooth (TM1) | ‘*Platypterygius*’ sp. | Cambridge |
| **IRSNB GS44** | Tooth (TM1) | ‘*Platypterygius*’ sp. | Cambridge |
| **IRSNB GS45** | Tooth (TM1) | ‘*Platypterygius*’ sp. aff. campylodon | Cambridge |
| **IRSNB GS46** | Tooth (TM1) | ‘*Platypterygius*’ sp. aff. campylodon | Cambridge |
| **IRSNB GS47** | Tooth (TM1) | ‘*Platypterygius*’ sp. | Cambridge |
| **IRSNB GS48** | Tooth (TM1) | ‘*Platypterygius*’ sp. | Cambridge |
| **IRSNB GS49** | Tooth (TM1) | ‘*Platypterygius*’ sp. | Cambridge |
| **IRSNB GS50** | Tooth (TM1) | ‘*Platypterygius*’ sp. | Cambridge |
| **IRSNB GS51** | Tooth (TM1) | Ichthyosauria indet. | Cambridge |
| **IRSNB GS52** | Tooth (TM1) | Ichthyosauria indet. | Cambridge |
| **IRSNB GS53** | Tooth (TM1) | ‘*Platypterygius*’ sp. aff. campylodon | Cambridge |
| **IRSNB GS54** | Basioccipital (BM2) | *Sisteronia seeleyi* | Cambridge |
| **IRSNB GS55** | Tooth (TM2) | *Sisteronia seeleyi* | Cambridge |
| **IRSNB GS56** | Tooth (TM2) | *Sisteronia seeleyi* | Cambridge |
| **IRSNB GS57** | Tooth (TM2) | *Sisteronia seeleyi* | Cambridge |
| **IRSNB GS58** | Tooth (TM2) | *Sisteronia seeleyi* | Cambridge |
| **IRSNB GS59** | Basioccipital (BM3) | ? Ophthalmosaurinae indet. | Cambridge |
| **IRSNB GS60** | Epipodial element | Ophthalmosaurinae indet. | Cambridge |
| **IRSNB GS61** | Basioccipital (BM2) | *Sisteronia seeleyi* | Cambridge |
| **IRSNB GS62** | Tooth (TM1) | ‘*Platypterygius*’ sp. aff. *campylodon* | Cambridge |
| **LEICT G65.1991** | Humerus (HM4) | Ophthalmosaurinae indet. | Cambridge |
| **LEICT G107.1991** | Basioccipital (BM2) | *Sisteronia seeleyi* | Cambridge |
| **NHMUK 35254 partim** | Numerous isolated teeth (TM1) | ‘*Platypterygius*’ sp. | Cambridge |
| **NHMUK 35254 partim** | Teeth (TM2) | *Sisteronia seeleyi* | Cambridgeshire |
| **NHMUK 35272 partim** | Basisphenoid | Ichthyosauria indet. | Cambridgeshire |
| **NHMUK 35272 partim** | Quadrate | Ichthyosauria indet. | Cambridgeshire |
| **NHMUK 35272 partim** | Quadrate | Ichthyosauria indet. | Cambridgeshire |
| **NHMUK 35272 partim** | Femur (FM2) | Ichthyosauria indet. | Cambridgeshire |
| **NHMUK 35272 partim** | Femur (FM2) | Ichthyosauria indet. | Cambridgeshire |
| **NHMUK 35301** | Basioccipital (BM3) | Ophthalmosaurinae indet. | Cambridgeshire |
| **NHMUK 35302** | Atlas-axis | Ichthyosauria indet. | Cambridgeshire |
| **NHMUK 35310** | Femur (FM1) | ‘*Platypterygius*’ sp. | Cambridgeshire |
| **NHMUK 35321** | Associated paddle elements | ‘*Platypterygius*’ sp. | Cambridgeshire |
| **NHMUK 35323 partim** | Basioccipital (BM1) | ‘*Platypterygius*’ sp. | Cambridgeshire |
| **NHMUK 35323 partim** | Articular | Ichthyosauria indet. | Cambridgeshire |
| **NHMUK 35348** | Humerus (HM4) | Ophthalmosaurinae indet. | Cambridgeshire |
| **NHMUK 35390** | Basisphenoid | Ichthyosauria indet. | Cambridgeshire |
| **NHMUK 35432** | Tooth (TM1) | ‘*Platypterygius*’ sp. | Cambridge |
| **NHMUK 35433** | Tooth (TM1) | ‘*Platypterygius*’ sp. | Cambridge |
| **NHMUK 35434** | Tooth (TM1) | ‘*Platypterygius*’ sp. | Cambridge |
| **NHMUK 40358** | Tooth (TM1) | ‘*Platypterygius*’ sp. | Cambridge |
| **NHMUK 40558** | Femur | Ichthyosauria indet. | Cambridgeshire |
| **NHMUK 41896** | Tooth (TM1) | ‘*Platypterygius*’ sp. | Trupington |
| **NHMUK 43989** | Humerus (HM4) | Ophthalmosaurinae indet. | Cambridgeshire |
| **NHMUK 44159** | Basioccipital (BM2) | *Sisteronia seeleyi* | Cambridgeshire |
| **NHMUK 44159a** | Basioccipital (BM2) | *Sisteronia seeleyi* | Cambridgeshire |
| **NHMUK 47265** | Numerous isolated teeth (TM1) | ‘*Platypterygius*’ sp. | ? |
| **NHMUK 47268 partim** | Tooth (TM3) | Ophthalmosaurinae indet. | Cambridgeshire |
| **NHMUK 47268 partim** | Tooth (TM3) | Ophthalmosaurinae indet. | Cambridgeshire |
| **NHMUK 47268 partim** | Tooth (TM3) | Ophthalmosaurinae indet. | Cambridgeshire |
| **NHMUK 47268 partim** | Tooth (TM3) | Ophthalmosaurinae indet. | Cambridgeshire |
| **NHMUK 47268 partim** | Tooth (TM3) | Ophthalmosaurinae indet. | Cambridgeshire |
| **NHMUK 47268 partim** | Tooth (TM3) | Ophthalmosaurinae indet. | Cambridgeshire |
| **NHMUK R625** | Tooth (TM1) | ‘*Platypterygius*’ sp. | Cambridge |
| **NHMUK R1133b** | Tooth (TM1) | ‘*Platypterygius*’ sp. | Cambridge |
| **NHMUK R1923 partim** | Tooth (TM2) | *Sisteronia seeleyi* | Cambridgeshire |
| **NHMUK R2336 partim** | Tooth (TM1) | ‘*Platypterygius*’ sp. | Cambridge |
| **NHMUK R2336 partim** | Tooth (TM1) | ‘*Platypterygius*’ sp. | Cambridge |
| **NHMUK R2337 partim** | Quadrate | Ichthyosauria indet. | Cambridgeshire |
| **NHMUK R2337 partim** | Quadrate | Ichthyosauria indet. | Cambridgeshire |
| **NHMUK R2337 partim** | Quadrate | Ichthyosauria indet. | Cambridgeshire |
| **NHMUK R2337 partim** | Quadrate | Ichthyosauria indet. | Cambridgeshire |
| **NHMUK R2341** | Basisphenoid | Ophthalmosaurinae indet. | Cambridgeshire |
| **NHMUK R2342 partim** | Humerus (HM5) | ‘*Platypterygius*’ sp. | Cambridgeshire |
| **NHMUK R2342 partim** | Humerus (HM5) | ‘*Platypterygius*’ sp. | Cambridgeshire |
| **NHMUK R2342 partim** | Femur (FM1) | ‘*Platypterygius*’ sp. | Cambridgeshire |
| **NHMUK R2343 partim** | Humerus (HM4) | Ophthalmosaurinae indet. | Cambridgeshire |
| **NHMUK R2343 partim** | Humerus (HM4) | Ophthalmosaurinae indet. | Cambridgeshire |
| **NHMUK R2343 partim** | Humerus (HM4) | Ophthalmosaurinae indet. | Cambridgeshire |
| **NHMUK R2344** | Femur (FM4) | Ophthalmosaurinae indet. | Cambridgeshire |
| **NHMUK R2348** | Opisthotic | Ichthyosauria indet. | Cambridgeshire |
| **NHMUK R2352** | Illium | Ophthalmosaurinae indet. | Cambridgeshire |
| **NHMUK R4512** | Jugal | Ichthyosauria indet. | Cambridgeshire |
| **NHMUK R4513** | Humerus (HM4) | Ophthalmosaurinae indet. | Cambridge |
| **NHMUK R4513** | Humerus (HM4) | Ophthalmosaurinae indet. | Cambridge |
| **NHMUK R4519** | Quadrate | Ichthyosauria indet. | Cambridgeshire |
