# Supplementary material for: High Diversity in Cretaceous Ichthyosaurs from Europe Prior to Their Extinction
Source: PLoS One. 2014 Jan 21;9(1):e84709. doi: 10.1371/journal.pone.0084709 (PMC3897400; doi:10.1371/journal.pone.0084709)
Supplement: Text S4 — Marnes Bleues Formation specimens studied here and their assignation. (DOC) [file pone.0084709.s004.doc]

**Text S4. Marnes Bleues Formation specimens studied here and their assignation.**

| **Specimen** | **Material** | **Assignation** | **Locality** |
| --- | --- | --- | --- |
| CM 33679 | Incomplete ribs | Ichthyosauria indet. | Saint-Dizier-en-Diois |
| CM 47527 | Incomplete snout | Ichthyosauria indet. | Saint-Dizier-en-Diois |
| RGHP SI 1 | Basioccipital, centra, tooth | ‘*Platypterygius*’ sp*.* | Sisteron |
| RGHP SI 2 | Incomplete skull | *Sisteronia seeleyi* | Sisteron |
| RGHP SI 3 | Rib | Ichthyosauria indet. | Sisteron |
| RGHP PR 1 | Incomplete skeleton | ‘*Platypterygius*’ sp*.* | Prads |
