# Supplementary material for: High Diversity in Cretaceous Ichthyosaurs from Europe Prior to Their Extinction
Source: PLoS One. 2014 Jan 21;9(1):e84709. doi: 10.1371/journal.pone.0084709 (PMC3897400; doi:10.1371/journal.pone.0084709)
Supplement: Text S5 — Determination key for isolated elements from the Cambridge Greensand Member. (DOC) [file pone.0084709.s005.doc]

**Text S5. Determination key for isolated elements from the Cambridge Greensand Member**

This review indicates the presence of four distinct taxa in the Cambridge Greensand Member. With the exception of *Cetarthrosaurus walkeri*, for which only two propodials are known, all taxa recognized here can be differentiated using basioccipital, opisthotics or humeri alone; this renders our method potentially useful in other ‘middle Cretaceous’ bone-bed-like localities. This section provides a quick key to determinate isolated to ichthyosaur bones in the Cambridge Greensand member and potentially other Albian–Cenomanian western European deposit. As mentioned above, some of the diagnostic features seem to appear and strengthen with ontogeny. Therefore, some of these criteria may not lead to identification when applied on juvenile specimen. Similar caveats are needed when determining teeth, because of a slight heterodonty along the tooth row. The most diagnostic teeth are the largest, found in the middle part of the rostrum. Anterior-most teeth are usually slender in all forms and posterior-most teeth are usually bulkier in all forms, and both have rounder root cross-section.

**Basioccipital**

1. Presence of an extracondylar area laterally and ventrally in posterior view. Yes: BM3, Ophthalmosaurinae indet.; no, go to 2.
2. Presence of prominent opisthotic facets and a raised constriction in the anterior half of the floor of foramen magnum. Yes: BM2, *Sisteronia seeleyi*; no, round basioccipital with no prominent parts: BM1, ‘*Platypterygius*’ sp.

**Tooth**

1. Root with rounded cross-section. Yes: TM3, Ophthalmosaurinae indet.; no, quadrangular cross-section: go to 2.
2. Robust tooth with markedly visible acellular cementum ring and squared root-section. Yes: TM1, ‘*Platypterygius*’ sp.; no, slender tooth and rectangular root cross-section: TM2, *Sisteronia seeleyi*.

**Humerus**

1. Elongated propodial with perfectly rounded capitulum and sheet-like trochanters. Yes: FM5, *Cetarthrosaurus walkeri*; no, stout humerus with rectangular capitulum in proximal view and thick trochanters: go to 2.
2. Humerus with posterior accessory facet. Yes: go to 3; no: go to 4.
3. Posterior accessory facet (not ulnar facet) is relatively large and markedly deflected posterolaterally. Yes: HM4, ‘*Platypterygius*’ sp.; no, HM2, Sisteronia seeleyi.
4. Radius and ulnar facet parallel to the sagittal plane. Yes: HM1, ‘Platypterygius’ sp.; no, ulnar facet markedly deflected posterolaterally: HM3, Ophthalmosaurinae indet.

**Femur**

1. Elongated propodial with perfectly rounded capitulum and sheet-like trochanters. Yes: *Cetarthrosaurus walkeri*; no, go to 2.
2. Two distal facets. Yes: go to 3; no: go to 4.
3. Distal end of the femur is thin compared to the shaft. Yes: FM3, Ichthyosauria indet.; no, the distal end is robust: FM2, Ichthyosauria indet.
4. Anterior facet is small and for an anterior accessory epipodial element. Yes: FM1, ‘*Platypterygius*’ sp.; no, this facet is for tibia and an astragalus facet is present: FM4, Ophthalmosauridae indet.
