# Supplementary material for: High Diversity in Cretaceous Ichthyosaurs from Europe Prior to Their Extinction
Source: PLoS One. 2014 Jan 21;9(1):e84709. doi: 10.1371/journal.pone.0084709 (PMC3897400; doi:10.1371/journal.pone.0084709)
Supplement: Text S6 — Description of indeterminate femoral morphotypes. (DOC) [file pone.0084709.s006.doc]

**Text S6. Description of indeterminate femoral morphotypes.**

**Material:** CAMSM B58061 (FM2 femur); CAMSM B58066 (FM2 femur); CAMSM B58067 (FM2 femur); CAMSM B58068 (FM2 femur); NHMUK 35272 (partim, 2 FM2 femora); CAMSM B58059 (FM3 femur); CAMSM B58065 (FM4 femur); NHMUK (R2344 partim, FM4 femur).

**Femur** (morphotypes 2, 3, 4 [FM2, FM3, FM4]). Femora grouped in the FM2 morphotype are medium sized, stout, with a triangular capitulum in proximal view and thickened distal end (proximal height / distal height ratio = 1.73, CAMSM B58066). The long-axis of the capitulum (the line joining both trochanters) does not form a right angle with the long axis of the distal end: the thick ventral crest lies closer to the anterior border of the femora and the oblique dorsal trochanter is set in a median position, as it is in ophthalmosaurid humeri. The femur possesses two distal facets: a semi-circular tibial facet and much larger and deeply concave fibular facet.

Femora grouped in the FM3 morphotype have a rounded to oval capitulum and enormous trochanters compared to the thin posterior end (proximal height / distal height ratio ratio = 3.05, CAMSM B58059). The posterior surface is not edgy and markedly rounded and the capitulum is therefore not triangular in proximal view. The anterior surface is a wide and flat triangle. The dorsal trochanter is plate-like, unlike in FM2, and both trochanters extend beyond mid-shaft. Distally, the femur possesses two oval distal facets for tibia and fibula. The fibular facet is slightly longer than the tibial one.

Femora grouped in the FM3 morphotype are similar to FM3 in having a slightly rounded capitulum from which the two large trochanters depart from, giving a rather oval morphology, as in *P. hercynicus* . The dorsal trochanter is not markedly plate-like and the femur possesses three distal facets of equal size. The middle one is set in a median position and pushing the two others outwardly, which face anterodistally and posterodistally. This morphology is typical in taxa where the intermedium/astragalus touches the humerus/femur (e.g. ).

References

1. Kolb C, Sander PM (2009) Redescription of the ichthyosaur *Platypterygius hercynicus* (Kuhn 1946) from the Lower Cretaceous of Salzgitter (Lower Saxony, Germany). Palaeontographica Abteilung A (Paläozoologie, Stratigraphie) 288: 151–192.

2. Maxwell EE, Caldwell MW (2006) A new genus of ichthyosaur from the Lower Cretaceous of Western Canada. Palaeontology 49: 1043–1052.
