# Supplementary material for: High Diversity in Cretaceous Ichthyosaurs from Europe Prior to Their Extinction
Source: PLoS One. 2014 Jan 21;9(1):e84709. doi: 10.1371/journal.pone.0084709 (PMC3897400; doi:10.1371/journal.pone.0084709)
Supplement: Text S7 — Taxa recognized as valid for each stage of the Hettangian–Turonian interval. (DOC) [file pone.0084709.s007.doc]

**Text S7. Taxa recognized as valid for each stage of the Hettangian–Turonian interval.**

| **Stage** | **Species** | **Genera** | **1** | **2** | **3** | **4** | **5** | **6** | **7** | **8** | **9** | **10** | **11** |
| --- | --- | --- | --- | --- | --- | --- | --- | --- | --- | --- | --- | --- | --- |
| **Het** | 3 | 3 | *T. platyodon* | *Lep. tenuirostris* | *I. communis* |  |  |  |  |  |  |  |  |
| **Sin** | 8 | 4 | *T. platyodon* | *Lep. tenuirostris* | *I. communis* | *T. eurycephalus* | *Lep. solei* | *Ex. costini* | *I. breviceps* | *I. conybeari* |  |  |  |
| **Pli** | 4 | 3 | *T. nuertengensis* | *Lep. tenuirostris* | *I. communis* | *Lep. moorei* |  |  |  |  |  |  |  |
| **Toa** | 11 | 5 | *Su. integer* | *Su. disinteger* | *St. Quadriscissus* | *St. Triscissus* | *St. uniter* | *H. typicus* | *T. acutirostris* | *T. crassimanus* | *T. trigonodon* | *T. azerguensis* | *Eu. longirostris* |
| **Aal** | 2 | 2 | *St. Aalensis* | Ophthalmosauridae indet | |  |  |  |  |  |  |  |  |
| **Baj** | 2 | 2 | *Mo. perialus* | *Ch. cayi* |  |  |  |  |  |  |  |  |  |
| **Bat** | 1 | 1 | Ichthyosauria indet. | |  |  |  |  |  |  |  |  |  |
| **Cal** | 2 | 1 | *O. icenicus* | *O. natans* |  |  |  |  |  |  |  |  |  |
| **Oxf** | 3 | 2 | *O. icenicus* | *O. natans* | *Ar. chrisorum* |  |  |  |  |  |  |  |  |
| **Kim** | 4 | 4 | *O. icenicus* | *N. enthekiodon* | *Ar. chrisorum* | *B. extremus* |  |  |  |  |  |  |  |
| **Tit** | 8 | 5 | *O. sp* | *Ae. leptospondylus* | *Ca. bonapartei* | *B. sp.* | *"O." gorodischensis* | *"O." yasykovi* | *"O." monocharactus* | *Ar. chrisorum* |  |  |  |
| **Ber** | 3 | 3 | *O. sp* | *Ae. sp.* | *Ca. bonapartei* |  |  |  |  |  |  |  |  |
| **Val** | 1 | 1 | *Ae. sp.* |  |  |  |  |  |  |  |  |  |  |
| **Hau** | 2 | 2 | *Mal. anachronus* | *Ac. densus* |  |  |  |  |  |  |  |  |  |
| **Bar** | 6 | 5 | *Sv. Insolitus* | Ophthalmosaurinae | *P. hauthali* | *Mal. anachronus* | *P. sachicarum* | *Sim. birjukovi* |  |  |  |  |  |
| **L Apt** | 3 | 2 | *P. sachicarum* | *P. platydactylus* | *Len. Stellans* |  |  |  |  |  |  |  |  |
| **U Apt** | 3 | 2 | Ophthalmosaurinae | *P. sachicarum* | *P. hercynicus* |  |  |  |  |  |  |  |  |
| **E Alb** | 6 | 5 | *At. bitumineus* | Ophthalmosaurinae | *Sis. seeleyi* | *P. campylodon* | *P. hercynicus* | *Mai. lindoei* |  |  |  |  |  |
| **M Alb** | 5 | 3 | *P. australis* | Ophthalmosaurinae | *Sis. seeleyi* | *P. campylodon* | *P. hercynicus* |  |  |  |  |  |  |
| **U Alb** | 8 | 4 | *P. australis* | Ophthalmosaurinae | *Sis. seeleyi* | *P. campylodon* | *P. hercynicus* | *P. americanus* | *P. ochevi* | *Ce. walkeri* |  |  |  |
| **Cen** | 5 | 2 | *P. americanus* | *P. bannovkensis* | *Sis. seeleyi* | *P. campylodon* | *P. ochevi* |  |  |  |  |  |  |
| **Tur** | 0 | 0 |  |  |  |  |  |  |  |  |  |  |  |

*Platypterygius* was considered as a single genus in the generic diversity curve. Abbreviations: Ac.: *Acamptonectes*; Ae.: *Aegirosaurus*; Ar.: *Arthropterygius*; At.: *Athabascasaurus*; B.: *Brachypterygius*; Ca.: *Caypullisaurus*; Ce.: *Cetarthrosaurus*; Ch.: *Chacaicosaurus*; Eu.: *Eurhinosaurus*; Ex.: *Excalibosaurus*; H: *Hauffiopteryx*; I.: *Ichthyosaurus*; Len.: *Leninia*; Lep.: *Leptonectes*; Mai.: *Maiaspondylus*; Mal.: *Malawania*; Mo.: *Mollesaurus*; N.: *Nannopterygius*; O.: *Ophthalmosaurus*; P.: *Platypterygius*; Sim.: *Simbirskiasaurus*; Sis.: Sisteronia; St.: *Stenopterygius*; Su.: *Suevoleviathan*; Sv.: *Sveltonectes*; T.: *Temnodontosaurus*.
